# Predictive modeling of gene expression regulation

**DOI:** 10.1186/s12859-021-04481-1

**Published:** 2021-11-27

**Authors:** Chiara Regondi, Maddalena Fratelli, Giovanna Damia, Federica Guffanti, Monica Ganzinelli, Matteo Matteucci, Marco Masseroli

**Affiliations:** 1 Dipartimento di Elettronica, Informazione e Bioingegneria, Politecnico di Milano, 20133 Milan, Italy; 2 Pharmacogenomics Unit, Istituto di Ricerche Farmacologiche Mario Negri, IRCCS, 20156 Milan, Italy; 3 Laboratory of Molecular Pharmacology, Istituto di Ricerche Farmacologiche Mario Negri, IRCCS, 20156 Milan, Italy; 4 Department of Medical Oncology, Fondazione IRCCS Istituto Nazionale dei Tumori, 20133 Milan, Italy

**Keywords:** Gene expression regulation, Regulatory network, Cancer, Predictive modeling, Machine learning

## Abstract

**Background:**

In-depth analysis of regulation networks of genes aberrantly expressed in cancer is essential for better understanding tumors and identifying key genes that could be therapeutically targeted.

**Results:**

We developed a quantitative analysis approach to investigate the main biological relationships among different regulatory elements and target genes; we applied it to Ovarian Serous Cystadenocarcinoma and 177 target genes belonging to three main pathways (DNA REPAIR, STEM CELLS and GLUCOSE METABOLISM) relevant for this tumor. Combining data from ENCODE and TCGA datasets, we built a predictive linear model for the regulation of each target gene, assessing the relationships between its expression, promoter methylation, expression of genes in the same or in the other pathways and of putative transcription factors. We proved the reliability and significance of our approach in a similar tumor type (basal-like Breast cancer) and using a different existing algorithm (ARACNe), and we obtained experimental confirmations on potentially interesting results.

**Conclusions:**

The analysis of the proposed models allowed disclosing the relations between a gene and its related biological processes, the interconnections between the different gene sets, and the evaluation of the relevant regulatory elements at single gene level. This led to the identification of already known regulators and/or gene correlations and to unveil a set of still unknown and potentially interesting biological relationships for their pharmacological and clinical use.

**Supplementary Information:**

The online version contains supplementary material available at 10.1186/s12859-021-04481-1.

## Background

Gene expression defines with good accuracy the activity and behavior of a gene inside a cell; its alteration may have different consequences on cells, including promoting the development of specific pathologies such as cancer [1]. In addition, not all genes are expressed at the same time in the same cells of a tissue and their expression vary quantitatively. Therefore, the study of the regulation of gene expression is fundamental for the understanding of the molecular traits in health and disease, and for the identification of therapeutic targets.

Due to the existence of multiple heterogeneous factors differently impacting gene expression, the regulation process is extremely complex and the understanding of how different elements regulate tumor gene expression is still limited. Efforts have been made in this direction with different computational approaches. For example, Poos et al. [2] created a software package for the R computational environment [3] to predict regulators of a gene of interest, starting from gene expression profiles of samples under study and known regulator binding information. Jiang et al*.* [4] focused their attention on the role of transcription factors in driving gene expression programs within a subset of 18 cancer types, and implemented a method to predict their oncogenic role, starting from an input gene expression file provided by the user or enabling to query a specific regulator or cancer. Recent efforts have been made in studying Gene Regulatory Networks (GRNs) using multiple different machine learning methods [5], including *information theory*, *Boolean networks*, *differential equations*, *Bayesian networks* [6], *neural networks*, and other network architectures. Many information theory-based methods have been proposed, due to their low computational cost, ability of discovering large GRNs from low expression data (such as, typically, those of transcription factor genes), and easy interpretability; they typically use scores such as mutual information and conditional mutual information to identify gene interactions, as in [7]). Boolean networks allow easy capturing of the dynamic behavior of GRNs by representing genes with Boolean variables, discretizing their expression level into binary values through clustering and thresholding, and using Boolean functions to reconstruct the network directed graph; yet, their main limitation is in the discretization step and the difficulty in dealing with noisy data. However, they are easy to interpret and have been proven useful in many cases, as in [8]. Conversely, ordinary differential equations use continuous variables, allowing more accurate dynamic modelling of gene regulation, and differential equations to represent gene expression changes as a function of the expression of other genes. Their disadvantage is the computational complexity, which prevents them from handling large GRN modelling despite often using only linear models or just specific types of non-linear functions, while regulatory processes have often complex non-linear dynamics. Yet, they have provided considerable results in GRN inference, as in [9]. Bayesian networks make use of the Bayes theorem of probability, combining probability and graph theory to model the properties of GRNs, whose graph is inferred from a set of conditional dependencies. Their main advantage is the flexibility, since they can combine different types of data and prior knowledge for reliable GRN inference, as in [10]. Neural networks include two main approaches: Artificial Neural Networks and Recurrent Neural Networks [5]; the latter ones also involve fuzzy logic and enable modelling non-linear and dynamic interactions among genes [11]. Tong et al. [12], employed the former ones to infer gene–gene interactions for biomarker discovery in childhood sarcomas. All these methods progressively emerged as promising and powerful approaches to investigate cell function control and provide clearer insights and understanding of cellular systems. In fact, GRN is a comprehensive map of living cell components reflecting the influence of genetic and epigenetic factors; it provides a great support to the study of complex diseases, pathway analysis and disease gene identification [13]. Integrating and analyzing different heterogeneous gene information can potentially provide a deep overview on complex biological systems and processes [5]; additionally, it can highlight the existing relationships between biological molecules, in order to explore the function of these individual molecules and the organization of the components of living cells.

With the aim to increase knowledge, we further explore this path by assessing the role of both transcription factors and promoter methylation. In this manuscript, we propose a novel computational technique to model gene expression regulation according to a predictive linear approach to possibly identify correlations between target genes and their putative regulators. The model allows building a large and explicative gene expression network that displays the main biological relationships between a gene and its regulators, including both already known and novel possible associations. These latter ones may provide insights on still unknown effects of the analyzed regulatory factors on gene activity and on other putative interesting biological connections. With respect to the state-of-the-art, the main innovative aspect of the approach we propose is the novel feature selection it embeds: rather than evaluating all considered candidate regulatory features of a target gene at once, we progressively enlarge the set of such features by considering different feature subsets at a time; at each step only the most relevant features selected in the previous step are preserved and re-evaluated together with the new considered features. This both reduces the computational complexity and allows disentangling the contribution of the different feature subsets, while the re-evaluation allows not incrementing the number of relevant features eventually selected. Thus, by considering feature subsets with different biological meaning, our approach allows unravelling both the most relevant features in the subsets and the relevant biological aspects overall for the problem under study. This enables the scientist to better understand the investigated biological system, through an approach that can be more adequate to analytically answer hypothesis-driven questions than other methods currently available.

Our quantitative analysis approach takes as input a set of user-defined target genes relevant for a phenotype (e.g., a tumor) of interest and investigates their regulatory systems, with the objective of identifying the most relevant features explaining the regulation of each target gene. For each of such genes, the aim is quantifying the effect of the expression of genes in the same gene set (or in other relevant gene sets, if considered), the impact of its promoter methylation [14] and of the expression of transcription factors binding its promoters [15]. The proposed approach is based on the expression of heterogeneous regulatory elements and it is very specific for retrieving the best-predicting sets of regulators, although possibly leaving out potential regulators with a lower predicting power. We specifically applied our approach to Ovarian Serous Cystadenocarcinoma, which is one of the main causes of death in women with gynecologic neoplasia [16]. The approach allowed us to identify and confirm already known gene correlations or regulators (e.g., hypermethylation of the *BRCA1* gene) and to unveil a set of still unknown and potentially interesting biological relationships for further experimental research. The strong aspects of our approach, besides the relevance of its results, are its simplicity and wide applicability.

## Methods

### Data sources, extraction and preparation

Using the GenoMetric Query Language (GMQL) system [17, 18] we extract and combine several heterogeneous data from three main public data repositories, i.e., genomic annotations from the Encyclopedia of Genes and gene variants (GENCODE) [19], transcription factor data from the Encyclopedia of DNA Elements (ENCODE) [20], and expression and methylation data from The Cancer Genome Atlas (TCGA) [21], which reports data from 33 tumor types.

Genomic localizations of human genes and their transcription start sites (TSSs) in assembly GRCh38 are extracted from GENCODE; gene promoter regions are derived around each gene TSS as an interval of 2000 bases upstream and 1000 bases downstream the TSS. Genomic annotation version 22, released in 2015, is used to ensure consistency with TCGA data, as TCGA adopted it for processing the considered expression and methylation data. This version annotates 60,483 genes (182,115 TSSs), including 19,650 protein coding genes (with their 71,839 TSSs).

**Fig. 1:**
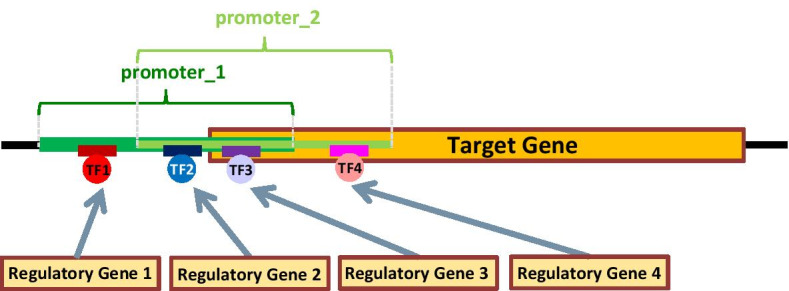
Transcription factor data extracted from ENCODE for each target gene

Candidate regulatory genes encoding transcription factors binding in promoter regions of tumor target genes are deduced from ENCODE as sketched in Fig. 1, using GRCh38 *narrow (point-source) conservative idr thresholded peaks* regions data obtained via ChIP-seq experiments; all promoter regions of a target gene, and all transcription factors binding in any of them, are considered.

**Fig. 2:**
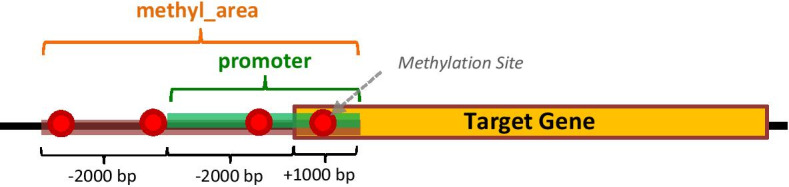
Methylation data extracted from TCGA for each target gene

Gene expression and methylation data related to the tumor under analysis are taken from TCGA, focusing on primary or recurrent tumor samples that were not previously subjected to neo-adjuvant treatment, excluding both metastatic tumor and normal samples. For each target gene, a single promoter methylation value is computed as the mean of the *beta_values* of all the probed methylation sites located within an extended promoter region (*methyl_area*, 4000 bases upstream and 1000 bases downstream a TSS) of the gene (Fig. 2), as broader areas than promoter regions may be involved in regulation by methylation [22]. Indeed, the methylation machinery is associated with several epigenetic mechanisms, like histone modifications, such that the regulation is afforded by complex protein interactions in addition to the binding of transcription factors [23]; for this reason, broader areas than promoter regions may be involved in regulation by methylation. Moreover, there is evidence, for instance in colon cancer, that most methylation alterations occur not in promoters, and also not in CpG islands, but in sequences up to 2 kb distant [22].

**Fig. 3:**
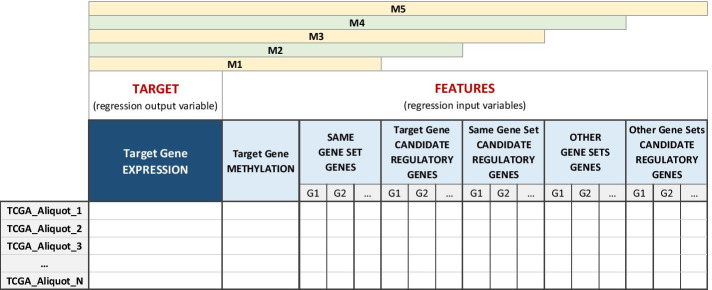
Structure of data matrices used for the analysis process of each target gene

To automatize and make computationally efficient the subsequent analysis of all these data, we structured them in a set of five incremental data matrices (M1–M5) for each target gene (Fig. 3), which are sequentially analyzed as described in “Data analysis” section. Given one or multiple sets of target genes of interest, these matrices are built as follows, with each matrix row regarding a single biological sample (identified by its *TCGA_Aliquot* ID):

- *Matrix M1* contains the expression of the target gene, its promoter methylation and the expression of the genes belonging to the same gene set as the target gene;
- *Matrix M2* adds the expression of all the candidate regulatory genes of the target gene to matrix M1, avoiding repetitions;
- *Matrix M3* adds the expression of the candidate regulatory genes of all the genes in the target gene set to matrix M2, avoiding repetitions;
- *Matrix M4* adds the expression of the genes belonging to the other, if they exist, gene sets of interest with respect to the considered target gene to matrix M3, avoiding repetitions;
- *Matrix M5* adds the expression of the candidate regulatory genes of all the genes belonging to the other, if they exist, gene sets of interest to matrix M4, avoiding repetitions.

This allows tracking each gene regulation system step-by-step, based on the different types of biomolecular features involved. The matrices considered more relevant and used for further analyses are M2, M3, M5.

### Specific application data

We applied our computational approach to investigate the regulatory system of 177 target genes belonging to 3 biological pathways (DNA REPAIR, STEM CELLS, GLUCOSE METABOLISM—Additional file 1: Section S0.1) relevant for the Ovarian Serous Cystadenocarcinoma (OV) [24–27], using all 372 TCGA OV patient samples with both RNA sequencing gene expression and methylation data publicly available (Additional file 1: Section S0.2).

Given the limited number of OV samples, to validate our OV results we used TCGA data of Breast Invasive Carcinoma (BRCA) samples of *basal-like* subtype according to the PAM50 molecular subtype classification; this BRCA intrinsic subtype had proved to be molecularly similar to Ovarian carcinoma [28]. All 122 TCGA basal-like BRCA samples with both gene expression and methylation data publicly available were used (Additional file 1: Section S0.3). As for ChIP-seq data, since ENCODE includes only a few OV experiment samples, we used those for MCF-7 (Breast cancer) and K562 (human immortalized myelogenous leukemia) cell lines, which are the ones in ENCODE with the highest number of ChIP-seq experiment samples (with 86 and 281 transcription factors (TFs), respectively, for a total of 308 distinct TFs); then, we evaluated the real activity of such possible candidate transcription factor encoding regulatory genes for the specific OV or BRCA patient through their expression value, which is available for each patient sample in the considered TCGA data. In so doing, we could indeed both consider an ample set of possible candidate direct regulatory genes and evaluate only those of them that are actually expressed in the specific patient sample considered; this is a relevant aspect of our designed approach.

### Data analysis

The aim of our defined multistep data analysis algorithm, where each different feature matrix is a different step in the analysis, is two-fold: quantifying the influence on the expression of target genes of each type of the many regulatory factors considered, while keeping a computationally scalable data analysis procedure. The main idea behind the novel approach we propose is progressively broadening the set of potential regulatory features considered for each target gene, at each step retaining only the most relevant ones from the previous step and re-evaluating them together with the new considered features. Also thanks to such feature re-evaluation shrewdness, this does not increase the small number of representative regulatory features finally selected for each target gene, which remain very limited with respect to all the considered ones. Our approach overcomes the intrinsic limitation of a purely greedy selection procedure and, at the same time, keeps the computational load under control, as a brute force approach would require an exponential number of models to be evaluated. The features identified as correlated with the expression of the target gene are the ones candidate as its regulatory factors, provided with a proxy of their estimated quantitative effect given by gene expression. For each target gene, given some sets of increasing candidate regulatory features, the data analysis proceeds in multiple steps, one for each of such feature sets and composed of two subsequent phases: a feature selection and a linear regression, as described below (details in Additional file 1: Section S1.1 and Section S1.2).

For each target gene, the defined data matrices M1–M5 incrementally include the different types of candidate regulatory features considered for the gene. Thus, they can be sequentially evaluated by our general data analysis algorithm to define the influence of each single type of features on the expression of the gene. Matrices M2, M3 and M5 are the most relevant ones; indeed, M2 groups the most likely candidate regulators of the target gene (promoter methylation, transcription factors and same-pathway genes), M3 includes all genes in the same set of interest of the target gene and their direct candidate regulators, while M5 adds the genes in the other considered sets of interest and their direct candidate regulators to M3. Thus, for a better biological interpretation, we progressively apply our algorithm on the M2, M3 and M5 matrices.

#### Feature selection

Each performed feature selection uses an incremental forward feature selection approach with re-evaluation of features selected in the previous analysis step, if any. In each of the multiple data analysis steps, the selection considers an additional new group of features along with only the features selected as most relevant in the previous step (if any), which are retained and re-evaluated. Then, it analyzes the linear regression performance of growing subsets of all such features, starting from a single feature and progressively adding all the others, one at a time based on the one that provides the best regression performance at that time; finally, it returns the feature subset with the best cross-validation performance. For better generalization, a fivefold cross-validation process is used: the set of available data samples is randomly split into five, possibly equal, groups of samples, each used as *test set* in one of five different feature selection processes using the remaining groups as *training set* (the same partition is used for processing all feature sets of all target genes considered). Lastly, only the features extracted in all five feature selections are selected as most relevant for the current analysis step of the target gene under analysis.

This strategy contributes to provide a scalable process, always keeping a limited number of considered features at each step of the data analysis, thus allowing to fit regression models on the reduced set of relevant variables [29]. The feature selection procedure is detailed in Additional file 1: Section S1.1, where it is shown in Additional file 1: Figures S1.2(a) and S1.2(b).

#### Linear regression

In each regression analysis, performed for each of the feature sets of different types selected for each target gene, the regulation system of the target gene is analyzed; the role of its possible regulatory features is identified and each feature impact on the target gene expression is quantified.

To allow subsequent comparisons of results not only within but also across regression models, Z-score normalization is applied across samples to obtain value distributions with *mean value* = 0 and *variance* = 1 for all variables. Then, for each target gene a linear regression model fitting is performed on each set of selected features using the ordinary least squares (OLS) method. From each modeling, only the features in the evaluated set that result in having a regression coefficient very unlikely null, i.e., whose 95% confidence interval does not include the 0 value, are extracted as significant; their regression coefficient quantifies the effect of the feature on the regulation of the target gene expression. Furthermore, the used *coefficient of determination* (*R*^2^ score) measures the quality of the regression fit, by comparing the residual sum of squares of the regression performed by the defined model against the regression of the model without any background knowledge, i.e., the null model [29]; for an unbiased measure, the *Adjusted R*^2^ is used, which adjust the *R*^2^ score for the number of features used in the model fitting into consideration on the basis of the sample size and the number of estimated coefficients. Processing details are in Additional file 1: Section S1.2.

**Fig. 4:**
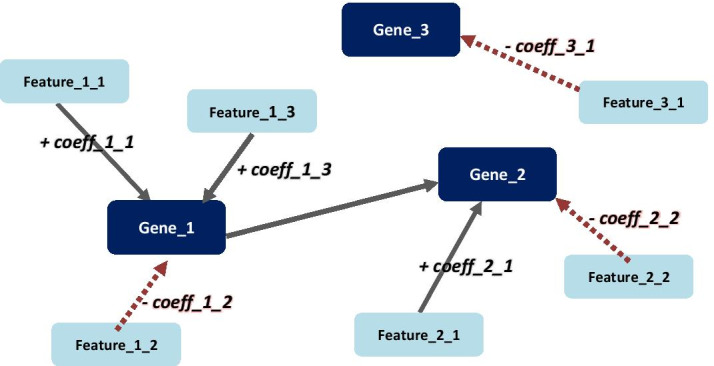
Network example visualizing linear regression results. Solid (black) and dashed (red) links indicate positive and negative effects of candidate regulatory features on target genes

Expression regulation networks are then inferred from the results of the linear regressions, per gene set and regression model, i.e., feature set type evaluated. The network nodes represent the target genes and their significant putative regulatory features; each target gene is connected to its features by directed incoming edges (from the feature to the target genes that the feature regulates), labelled with the calculated linear regression coefficient, quantifying the positive or negative effect the feature has in the target gene expression regulation system. Figure 4 displays an example network showing what we expect from the results of our algorithm.

### Comparison with ARACNe

To evaluate the validity of our approach, we compared it with a widely used method for computing expression correlations among sets of genes and for computationally inferring mutual functional relationships: ARACNe [7, 30]; we chose it for comparison since, as our proposal, it is able to extract highly interpretable knowledge from data, using an information theoretic approach. Furthermore and more importantly, to our knowledge ARACNe is the only information theory based method implementation that lets specify a priori information for the network inference (i.e., *Hub Genes*, the network nodes, and *Transcription Factors*, the candidate regulators), as our approach does; this makes appropriate the comparison of our approach with ARACNe only.

ARACNe builds gene expression networks by iteratively considering triples of genes and removing the weakest of the three relationships at each iteration. It does so according to a Mutual Information (MI) value, which defines the relationship strength, and to an arbitrary threshold (I0) on the MI, set a priori by the user. This threshold allows defining the dimension of the generated network: the higher I0, the smaller the network and the number of displayed relationships (i.e., only the strongest ones).

We used ARACNe for evaluating the associations and correlations found during the data analysis, comparing the expression networks computed with our regression models with the corresponding networks generated by the ARACNe algorithm, and identifying the common gene features (i.e., regulatory genes) and their association ranking. For a proper comparison with the M3 and M5 models of our approach, we applied the ARACNe algorithm separately to each individual biological pathway considered (DNA REPAIR, STEM CELLS and GLUCOSE METABOLISM): After merging all M3 or M5 data matrices of each target gene in the pathway (removing all duplicate genes in case present), we used each of the two merged input data matrix (without any feature pre-selection) as *Table Data* on which to run the ARACNe algorithm, setting the target genes of the pathway as *Hub Genes* (i.e., network nodes), the candidate regulatory genes of the pathway as *Transcription Factors*, and the considered TCGA data sample IDs as *Data Attributes*. Thus, also in ARACNe we used the same data and pre-defined set of genes derived from the considered pathways as a priori information, providing both the target genes and the candidate transcription factors as in our approach.

### Implementation

We implemented our new general approach in a comprehensive and generalized Python library, called *genereg* (https://pypi.org/project/genereg/), which can be used to investigate relationships in any type of tumor for which TCGA data is available. The library also implements alternative feature selection procedures that can be applied during the data analysis process, as an alternative to our incremental forward feature selection with features re-evaluation, as described in Additional file 1: Section S4.1.

## Results

In this Section, we report the results obtained for each considered pathway gene set by applying our approach on OV data (focusing on matrices M3 and M5); we also show the outcomes of the comparison with the results obtained for BRCA data and from the ARACNe alternative computational method (details are in Additional file 1: Sections S1.3, S2 and S3). Finally, we show the experimental validation results of some of our findings, and the comparison results with alternative feature selection strategies.

In addition to the predictive models, in our GitHub repository (https://github.com/DEIB-GECO/genereg, path *OV Cancer Results / Data Compendium for Researchers.zip*) we supply a compendium of data useful for researchers interested in studying the three pathways here considered, including: (1) transcripts, methylation and expression values in TCGA Ovarian and basal-like Breast cancers; (2) correlations between methylation and gene expression (a scatterplot for each gene of interest); and (3) the list of putative transcription factors from ENCODE for each target gene.

### Overall results on ovarian cancer pathways

The adopted incremental approach aims at identifying the features, among the considered ones, that are more relevantly correlated with the regulation of a target gene expression, rather than fully explaining the gene regulatory system. We accepted values as low as 0.6 for the Adjusted R^2^ of our regression models to indicate a good linear model fit, with the value of the regression coefficient of the features identified as relevant that quantifies the effect of the feature on the target gene expression regulation.

Typically, model accuracy increases by adding new features; our multistep analysis, increasingly adding features, leads to progressively broadening the set of regulation hypotheses, until reaching a set of features that allow an accurate prediction of the gene expression model. However, some genes may better fit in their first (M2) or second (M3) model, rather than in the last one (M5), showing that their regulation system mainly depends on the activity of genes in the same pathway or of their candidate regulatory genes. Final results are visualized as a set of networks, defined by grouping target genes according to their function-specific classification. All the networks are reported in Additional file 1: Section S3, while in the following we discuss some specific findings.

#### DNA REPAIR pathway

**Fig. 5:**
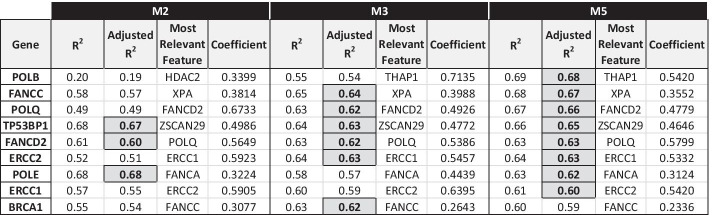
DNA REPAIR genes with M3/M5 model Adjusted R^2^ score > 0.6

The genes involved in the DNA REPAIR pathway that overall show the best linear fit in the regression models are 9: *BRCA1*, *ERCC1*, *ERCC2*, *FANCC*, *FANCD2*, *POLB*, *POLE*, *POLQ* and *TP53BP1* (Fig. 5).

*Model M3*: for 5 genes of the pathway, the gene promoter methylation is demonstrated as one of the relevant features involved in the regulation of their expression (i.e., in their repression, by the sign of its coefficient): *BRCA1* (coeff. =  − 0.3442), *ERCC1* (coeff. =  − 0.1347), *ERCC4* (coeff. =  − 0.1817), *ERCC5* (coeff. =  − 0.1630), *FANCF* (coeff. =  − 0.1314).

There are 4 most frequent regulators, each appearing in the regulatory models of four genes in the DNA REPAIR set:

- *POLQ* (gene of DNA REPAIR pathway);
- *FANCD2* (gene of DNA REPAIR pathway);
- *SUZ12* (candidate TF of a gene of DNA REPAIR pathway;
- *ZHX1* (candidate TF of a gene of DNA REPAIR pathway).

*Model M5*: for 4 genes of the pathway, the gene promoter methylation is selected as one of the relevant features involved in the regulation (repression) of their expression: *BRCA1* (coeff. =  − 0.3475), *ERCC4* (coeff. =  − 0.2608), *ERCC5* (coeff. =  − 0.1774), *FANCF* (coeff. =  − 0.1659).

There is one single most frequent regulator, appearing in the regulatory models of five genes in the DNA REPAIR set:

- *XRCC3* (candidate regulatory gene of STEM CELLS pathway).

This last result highlights the interrelationship between DNA REPAIR and STEM CELLS pathways and the relevant impact that genes in the latter one can have in regulating the activity of genes involved in the DNA damage repair mechanisms.

As a whole, we could not find common transcription factors regulating the expression of all (or most) genes involved in the same DNA REPAIR pathway. This is partly unexpected, but there can be several reasons for this finding. A possible explanation could be that the DNA REPAIR pathway is a multistep process involving many different proteins having roles also in other cellular processes. It may be that under specific conditions (i.e., DNA damage or other cell stress stimuli) different processes are activated. Moreover, the altered expression of a transcriptional factor is one of the possible alterations that may affect its ability to regulate its target gene. Several other modifications, such as protein levels, post-translational modifications and subcellular localization are relevant, but cannot be captured by transcriptomic data and are very difficult to assess quantitatively also in proteomic analyses.

#### STEM CELLS pathway

**Fig. 6:**
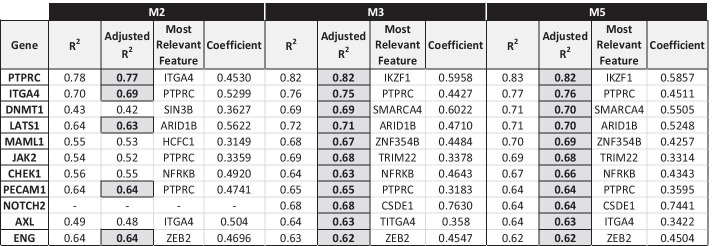
STEM CELLS genes with M3/M5 model Adjusted R^2^ score > 0.6

The genes involved in stem cells that overall show the best linear fit in the regression models are 11: *AXL*, *CHEK1*, *DNMT1*, *ENG*, *ITGA4*, *JAK2*, *LATS1*, *MAML1*, *NOTCH2*, *PECAM1* and *PTPRC* (Fig. 6).

*Model M3*: for 10 genes of the pathway, the gene promoter methylation is selected as one of the relevant features regulating their expression: *ATM* (coeff. =  − 0.1591), *CD34* (coeff. =  − 0.1733), *CHEK1* (coeff. =  − 0.0960), *CXCL8* (coeff. =  − 0.1628), *DACH1* (coeff. =  − 0.1215), *EPCAM* (coeff. =  − 0.2276), *MAML1* (coeff. =  − 0.0723), *PLAT* (coeff. =  − 0.2482), *POU5F1* (coeff. =  − 0.1984), *SAV1* (coeff. =  − 0.1725).

There is one single most frequent regulator, appearing in the regulatory models of 10 stem cells genes:

- *PAX8* (candidate TF of a gene of STEM CELLS pathway).

*Model M5*: for 9 genes of the pathway, the gene promoter methylation is selected as one of the relevant features regulating their expression, i.e., as in model M3, with the exception of gene DACH1: *ATM* (coeff. =  − 0.1591), *CD34* (coeff. =  − 0.1733), *CHEK1* (coeff. =  − 0.0960), *CXCL8* (coeff. =  − 0.1628), *EPCAM* (coeff. =  − 0.2276), *MAML1* (coeff. =  − 0.0723), *PLAT* (coeff. =  − 0.2482), *POU5F1* (coeff. =  − 0.1984), *SAV1* (coeff. =  − 0.1725).

The same most frequent regulator is present, selected as a relevant regulatory feature for the same 10 stem cells target genes as in model M3:

- *PAX8* (candidate TF of a gene of STEM CELLS pathway).

Interestingly, no reciprocal influence between STEM CELLS and DNA REPAIR pathway genes is seen, with no relevant impact of the latter ones on the former genes, whose regulation systems mainly depend on genes or candidate regulatory genes of the STEM CELLS pathway itself.

#### GLUCOSE METABOLISM pathway

**Fig. 7:**
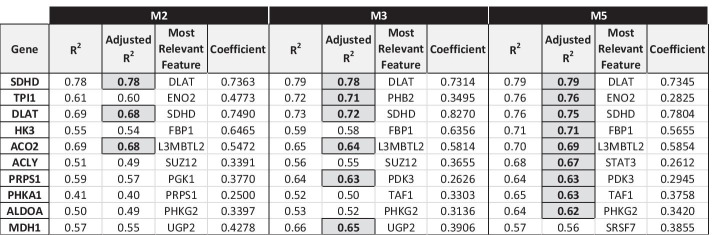
GLUCOSE METABOLISM genes with M3/M5 model Adjusted R^2^ score > 0.6

The genes involved in the glucose metabolism that overall show the best linear fit in the regression models are 10: *ACLY*, *ACO2*, *ALDOA*, *DLAT*, *HK3*, *MDH1*, *PHKA1*, *PRPS1*, *SDHD* and *TPI1* (Fig. 7).

*Model M3*: for 12 genes of the pathway, the gene promoter methylation is selected as one of the relevant features involved in the regulation of their expression: *AGL* (coeff. =  − 0.0917), *ALDOC* (coeff. =  − 0.3614), *DLD* (coeff. =  − 0.2463), *IDH3B* (coeff. =  − 0.2262), *MDH2* (coeff. =  − 0.1419), *PCK1* (coeff. =  − 0.3192), *PDK3* (coeff. =  − 0.1905), *PDK4* (coeff. =  − 0.1139), *PGM3* (coeff. =  − 0.1658), *PYGM* (coeff. =  − 0.1122), *RPE* (coeff. =  − 0.1595), *SDHA* (coeff. =  − 0.2319).

There is one single most frequent regulator, selected as a relevant regulatory feature for 8 GLUCOSE METABOLISM target genes; it is:

- ILK (candidate TF of a gene of GLUCOSE METABOLISM pathway).

*Model M5*: for 12 genes of the pathway, the gene promoter methylation is selected as one of the relevant features involved in the regulation of their expression (the same genes as in model M3, with the exception of gene PDK4 and the addition of gene TKT): *AGL* (coeff. =  − 0.0896), *ALDOC* (coeff. =  − 0.3334), *DLD* (coeff. =  − 0.2559), *IDH3B* (coeff. =  − 0.2132), *MDH2* (coeff. =  − 0.1513), *PCK1* (coeff. =  − 0.3547), *PDK3* (coeff. =  − 0.2408), *PGM3* (coeff. =  − 0.1134), *PYGM* (coeff. =  − 0.1141), *RPE* (coeff. =  − 0.1718), *SDHA* (coeff. =  − 0.2191), *TKT* (coeff. =  − 0.0819).

There is one single most frequent regulator, selected as a relevant regulatory feature for 10 GLUCOSE METABOLISM target genes:

- TSC22D4 (candidate TF of a gene of STEM CELLS pathway).

These results highlight the limited effect of the DNA REPAIR pathway in the regulation of GLUCOSE METABOLISM genes and the higher interrelationship between the GLUCOSE METABOLISM and STEM CELLS pathways, with the latter one having a key role in the regulation systems of the former one.

### Cross-application on BRCA dataset

**Fig. 8:**
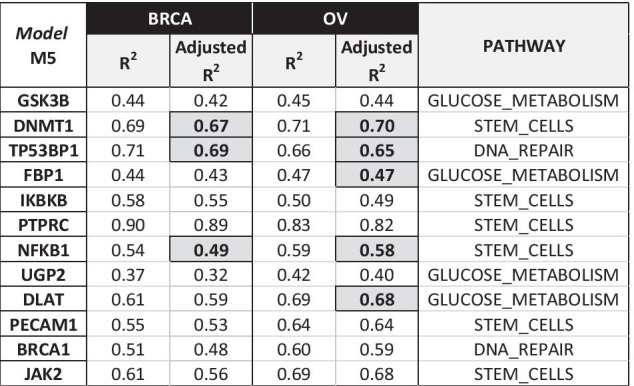
Application of OV models on BRCA data: genes with Adjusted R^2^ (OV) ≥ 0.4 and similar M5 Adjusted R^2^ (± 0.1) in the two datasets

To assess the relevance of our approach and its results, we first evaluated if the same models obtained for the Ovarian cancer data could be applied to a different set of comparable data. To this aim, we used basal-like Breast cancer subtype data, which bear significant similarities to OV data [28]. Indeed, this subtype, which corresponds to triple-negative Breast cancers has been reported to carry extensive genomic rearrangements and allelic imbalance and to share similar defects in DNA repair to serous Ovarian cancers [31]. For each target gene g, we computed the estimated value of its expression (EXPR_g_) in a BRCA sample according to the linear regression:

$${\text{EXPR}}_{{\text{g}}} = {\text{c}}_{1} {\text{v}}_{{{\text{f}}1}} + {\text{c}}_{2} {\text{v}}_{{{\text{f}}2}} + \cdots + {\text{c}}_{{\text{i}}} {\text{v}}_{{{\text{fi}}}} + \cdots + {\text{c}}_{{\text{n}}} {\text{v}}_{{{\text{fn}}}}$$

where v_fi_ are methylation or expression values in the BRCA sample of the n features f_i_ extracted as relevant, with their associated regression coefficients c_i_, in the considered OV regression model for the same gene g. Finally, we computed the regression R^2^ and Adjusted R^2^ values and compared them with the corresponding ones in the original OV models. A set of key genes (shown in Fig. 8 with their M5 model values) appeared to be similarly regulated in the two tumor types, confirming the relevance and robustness of our approach.

**Fig. 9:**
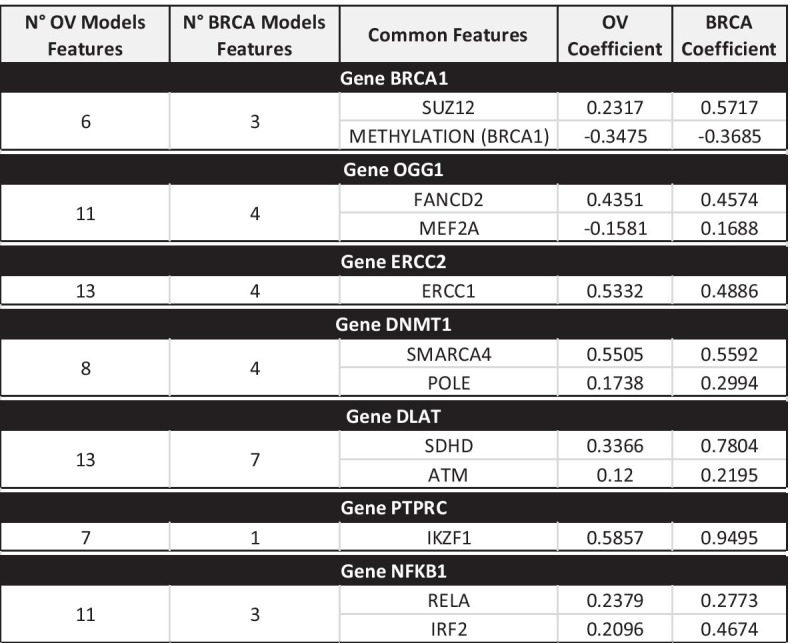
OV versus BRCA regression model comparison for M5 (for a sample set of genes)

Furthermore, we completely re-computed the regression models M2, M3 and M5 on the same BRCA dataset. Even if lower quality results were expected due to the limited number of available basal-like BRCA samples, the most relevant features in the OV M3 and M5 models were also detected in BRCA models, being genes in the DNA REPAIR pathway the most common outcomes. In addition, the regression coefficients assigned to common features in the two tumor models are interesting (Fig. 9).

In most cases, they showed similar values, suggesting that the impact of the feature on the target gene expression is similar in these tumors. These data suggest DNA-damage-related similarities of the two tumors, corroborating data from the literature [31]. Conversely, different regression coefficients for the same feature in these two tumor models might potentially be associated with tumor-specific characteristics.

### Results of comparison with ARACNe

We evaluated our results also comparing them with those by ARACNe for the OV data. Despite their different approach, the two techniques found common correlations for a considerable percentage of target genes. In fact, 30% of the whole set of relevant regulatory features identified in our regression models was confirmed by ARACNe. More relevantly, the rankings of the common features in the two techniques were very similar, with only few pairs of features swapped in their order. This very high similarity of ranking further supports our regression results, suggesting that the identified features and their relevance on the expression of the modeled genes are likely correct (as we experimentally validated for some of them). More details of this comparison are in Additional file 1: Section S2.

While ARACNe evaluates gene triplets at a time considering all possible existing correlations, our approach directly fits a linear model, analyzing a set of candidate regulators for each target gene separately and independently from each other. Thus, our approach is more precise, being able to quantify the impact of the regulatory elements of each target gene individually. This could enable understanding if the influence that a gene has on another one is reciprocal, or not. Moreover, our modeling can include heterogeneous regulatory factors, such as methylation, while ARACNe is limited to analyzing only expression correlations without the possibility to create heterogeneous models including other regulatory mechanisms.

### Experimental validation of found biological correlations in independent ovarian and breast cancer samples

Among all regulatory networks predicted by our approach, we focused on some specific biologically interesting gene correlations for which we could obtain experimental evidence.

Among the predictive models for DNA-damage genes, an interesting case is *ERCC2* (*XPD*). For this gene, *ERCC1* is the most representative feature in all three models in OV (Fig. 5) and in basal-like BRCA (Fig. 9). *ERCC1* and *ERCC2* genes belong to the nucleotide excision repair (NER) pathway involved in the repair of UV-induced DNA damage [32]. The pathway has also a key role in the repair of DNA adducts induced by cisplatin [33, 34]. We were able to confirm this correlation both in OV and in BRCA samples from our laboratory, whose origin is detailed in [27] and [35], respectively. In particular, the correlation between the expression of *ERCC1* and *ERCC2* in 42 Ovarian cancer patient-derived xenografts (PDX) [27], as measured by quantitative RT-PCR, was r = 0.71, *p*-value = 1.4 × 10^−7^. Similarly, *ERCC1* and *ERCC2* expression levels were significantly correlated (r = 0.61, *p*-value = 2 × 10^−5^, n = 75) in triple negative, but not correlated (r =  − 0.10, *p*-value = 5 × 10^−1^, n = 68) in luminal A Breast cancer patients [35].

Another interesting gene from the DNA-damage gene set is *CDK12*, whose most relevant feature in the OV M5 model is *SUZ12* (Additional file 1: Section S1.3.1). *SUZ12* expression was found decreased by 12% and 62%, in Ovarian cancer cells with a homozygous and heterozygous deletion in *CDK12*. Interestingly, this experimental observation adds a directionality in this cause-effect relationship. In fact, it demonstrates not only that *CDK12* expression levels are directly correlated with *SUZ12* levels, but also that *CDK12*, being a kinase involved in transcription [23], possibly regulates *SUZ12* expression.

Among our STEM CELLS pathway gene models, *DNMT1* (DNA Methyltransferase 1) was found to be associated with *CHEK1* (Checkpoint Kinase 1) (Additional file 1: Figure S1.5). *DNMT1* has been reported to have different cellular functions, including regulating genome integrity as an early responder to DNA double strand breaks [36, 37], while *CHEK1* is mediator of the DNA damage response pathway [38]. A similar experimental correlation was demonstrated in our OV cancer PDX models (r = 0.56, *p*-value = 3 × 10^−5^, n = 49). Instead, *RAD51*, *RB1* and *FANCA*, which are relevant features in our *CHECK1* predictive model, are only weakly (r = 0.26, *p*-value = 6.8 × 10^−2^, n = 50) or not correlated (r =  − 0.17, ns, n = 34 and r = 0.21, ns, n = 34), respectively, in the same PDX models. However, when we looked for similar correlation in a conditional *CHECK1* knock-out HCT116 carcinoma cells [39] we found that *CHECK1* downregulation associated with a reduction in *DMNT1*, *RAD51* and *FANCA* expression levels (0.47 ± 0.07, 0.49 ± 0.02 and 0.35 ± 0.05 fold change in silenced vs. control cells, respectively), supporting a positive *CHEK1* role in regulating the levels of these three genes. It is worth highlighting that, out of these three experimentally validated relationships of *CHECK1*, ARACNe predicted only the relationship between *CHEK1* and *RAD51*, but not the ones between *CHEK1* and the *DMNT1* and *FANCA* genes, which were all predicted by our approach. The other experimentally validated gene relationships (*ERCC1–ERCC2* and *SUZ12–CDK12*) were predicted by both our approach and ARACNe.

### Results of comparison with alternative feature selection methods

As far as regards the feature selection procedure, we evaluated the results of our described approach (*incremental feature selection with feature re-evaluation*) combined with *forward feature selection (FFS)*, hereafter *incremental FFS with feature re-evaluation*, and with the fivefold cross-validation process by comparing them with the results of other four alternative feature selection strategies, described in Additional file 1: Section S4.1: *incremental FFS with no feature re-evaluation*, *FFS on all features*, *incremental Lasso feature selection with feature re-evaluation*, and *Lasso feature selection on all features*. The first two strategies follow the same principle (FFS) combined with our proposed approach, but they differ in the way the features selected in previous steps are treated (without feature re-evaluation, or considering all features together); conversely, the last two methods are based on the Lasso algorithm [29] (one with re-evaluation of the features, the other one considering all features together). Also all four alternative methods considered are combined with the fivefold cross-validation process.

The results in terms of number of selected features for all considered target genes by each of the five feature selection strategies applied on the same OV data are detailed in Additional file 1: Section S4.2, together with the comparisons between the results obtained through our approach or using the alternative methods, and are summarized in Additional file 1: Subsection S4.2.6. All comparisons show that a relevant number of the features that our approach combined with FFS selects are selected also by the other methods. This consensus confirms the reliability of our approach, which in addition generally selects less features, but not in a statistically significant lower amount, than the other methods, facilitating the interpretability of the provided results. The only exception was the forward feature selection method applied on all features together, which resulted selecting statistically significantly less features than our approach combined with FFS, but the only one that did not identify the relationship between the *DNMT1* and *CHEK1* genes, despite it has been confirmed in both OV cancer PDX models and in a model of conditional *CHECK1* knock-out in HCT116 cultured ovarian carcinoma cells; all other experimentally confirmed relationships that we tested (see previous subsection) were identified by all considered methods.

Despite lab experimental evaluation is the only one that can evaluate the real correctness of the predicted gene relationships, only a limited number of relationships can be experimentally evaluated due to time and cost constraints; thus, to more comprehensively assess the goodness of the predictions provided by our and by the other considered methods, we computed the consensus of the methods on the relevant regulatory features extracted, under the assumption that the more a feature selected by a method is selected also by other methods, the more such feature may be likely correct (despite more methods may commit the same mistake on that feature). Furthermore, as additional estimation proxy for the quality of the features selected by a method as candidate gene regulators of the target gene in gene regulatory networks, we computed the performance of these features in predicting the target gene expression. (Notice however that the aim of our method is the prioritization of such genes/features, with the extraction of their relevant ones, rather than the identification of all or most of them, which typically can provide a statistically better, but less biologically meaningful, modeling and prediction of gene expression.) Since the aim of this latter evaluation is identifying the method that selects the features providing better modelling (i.e., prediction) of the expression of a target gene, we resorted to the computation of the *Bayesian Information Criterion* (BIC) for every model that each method could define for each considered target gene, evaluating the average BIC value for each method. BIC is a well-known criterion for model selection among a finite set of models [40], with the lowest BIC indicating the preferred model. Compared with other model selection criteria, such as *Akaike Information Criterion* (AIC) or *Adjusted R*^*2*^, BIC favors parsimonious models and takes into account the number of samples available for model fitting besides the number of features used in the model.

The obtained results (reported in Additional file 1: Subsection S4.2.6, together with the execution time of each method) show that the FFS applied on all features together is the method that extracts the lower total number of features (344) for the considered target genes and the one with the highest percentage (89.24%) of such features extracted also by all other 4 methods considered; however, it has the worst average BIC value and the far highest computational time (325 h). Conversely, the Lasso feature selection is by far the fastest one (not surprisingly, having been developed for this purpose) and shows the lowest average BIC value. However, with respect to the FFS method it can select relevant features (i.e., compute a model) for much less than the 177 considered target genes (only 155 vs. 171 when applied on all features together, or 163 vs. 175 with incremental feature selection with re-evaluation); furthermore, it extracts the highest number of features (410 vs. 344, or 425 vs. 383), with only less than 75% of them extracted also by all other 4 methods considered.

The evaluation results also clearly show that the incremental feature selection with re-evaluation, which we propose, combined with either the FFS or Lasso method can both increase the number of computed models (i.e., target genes for which relevant features are selected) and improve (i.e., lower) their average BIC value with respect to evaluating all features together. Furthermore, in the case of FFS it strongly lowers the overall computational time and provides a high percentage of selected features that are selected also by all other 4 methods (80.16%), and the highest percentage of the remaining features (72.37%) that are selected also by other 3 of the 4 methods considered; thanks to the feature re-evaluation, this is obtained with only a small increase of the total number of relevant features extracted (increase expected since the incremental selection can identify also features relevant within biologically specific subsets of features, which may be difficult unveiling when more feature types are considered together).

## Discussion

We developed a generalized quantitative analysis approach to investigate the main biological relationships among different regulatory elements and target genes. It provides a computational prioritization of candidate regulatory elements for modeling gene regulation in specific functional gene sets.

The sets of heterogeneous data needed for the analysis were extracted from main biological and genomic data sources. Data on transcription factors and on the expression of their encoding genes, along with expression and methylation values associated with target genes, were then arranged, for each target gene, in multiple data matrices with a fixed number of rows (i.e., the patient data samples) and a variable number of columns (i.e., the set of potential features affecting the model gene expression), gradually increasing by biological feature type according to pre-defined biologically-driven rules. Our approach used these sets of biological features as inputs to build three predictive models for each target gene. A preliminary step of feature selection was followed by the application of the linear regression algorithm for inferring most relevant features, either up-regulating or down-regulating the expression of the model gene. The progressive enlargement of the considered features by biological type is a relevant novel contribution of our designed approach, which allows quantifying the influence on the expression of target genes of each type separately of the many regulatory factors considered. This supports the scientist in better deciphering the examined biological system through an approach well suited for hypothesis-driven investigations.

The approach of incremental feature selection with re-evaluation we propose (particularly, but not only, when combined with the forward feature selection method) can provide better results than other equivalent state-of-the-art methods for the aim of prioritizing (i.e., extracting few but relevant) regulatory features in gene regulatory networks; in fact, it provides a better trade-off between (low) number of features extracted, (high) consensus with other methods on the extracted features, and (high) performance in modeling/predicting gene expression (in the absence of extensive wet lab experimental validation due to time and cost constraints). Anyway, the novel incremental feature selection with re-evaluation that we propose can straightforwardly be combined also with other feature selection methods than FFS, e.g., Lasso (available in the implementation that we provide in the GitHub repository) in case faster execution time is needed and other preference criteria can be relaxed (e.g., accepting features extracted in higher amount and with less consensus with other methods).

The models obtained by our approach do not fully explain the regulatory networks of each gene; in fact, their best Adjusted R^2^ scores are below 0.8. Indeed, a complete explanation was not expected, as several other factors are known to contribute to the regulation of gene expression in addition to the expression levels of other genes (e.g., non-coding RNAs, protein levels that may not completely correlate with mRNA levels, protein post-translational modifications and subcellular localization) [41, 42]. However, due to the wider availability and higher reliability of gene expressions as compared to the other types of data, the information provided by the models, although not complete, is very useful and relevant.

We demonstrated with good results the possibility of generalizing the obtained gene to gene relationships (i.e., the developed computational model) by applying the model obtained in the Ovarian cancer study to the study of the basal Breast cancer, a pathophysiological similar hormone-dependent tumor with an important role of the DNA damage pathway. Taking into account the underlying biological characteristics is the proper way to generalize the obtained computational results, in order to allow their adequate biological interpretation. The developed model could also be applied to all tumor types present in the TCGA dataset. However, in this case, in order to obtain more general regulatory networks and correctly identify conserved versus specific regulatory features, a higher number of gene sets should be used, including pathways ubiquitously activated in cancer, such as those regarding the regulation of cell cycle.

## Conclusions

The analysis of the proposed models allowed disclosing the relations between a gene and its related biological processes, the interconnections between the different gene sets, and the evaluation of the relevant regulatory elements at single gene level. This led to the identification of already known regulators and/or gene correlations (e.g., hypermethylation of *BRCA1* in OV tumor) and to unveil a set of still unknown and potentially interesting biological relationships (e.g., the correlation between *ERCC1* and *ERCC2*, or between *CHEK1* and *DNMT1* in OV) for their pharmacological and clinical use. Indeed, the experimental validation of the latter two correlations, the latter one predicted by our approach but not by ARACNe, supports the importance and reliability of our computational approach, which we hope will help disclosing new important druggable targets.

As a future extension of the current work, the additional testing of how well the developed models perform on a new appropriate held-out dataset, different from the one on which the created models were trained and validated but equivalent to it, could give further interesting insights. Particularly, by comparatively performing such testing using different alternative feature selection methods combined or not with our proposed incremental feature selection approach, it can provide a useful additional assessment of our approach and of the alternative methods, as well as of the selected features.

## Supplementary Information

**Additional file 1**: **S0** List of genes and patient samples considered, **S1** Additional aspects of the developed data analysis algorithm and obtained results, **S2** Further information about the performed computational evaluation using ARACNe, **S3** Gene expression networks generated, **S4** Alternative feature selection methods compared.

## Data Availability

At https://github.com/DEIB-GECO/genereg we provide both a useful compendium of data for the evaluated genes and tumors (comprising promoter methylation and putative transcription factors) and an implementation, with its source code, of the proposed approach, usable for other gene sets and tumor types, also available at https://pypi.org/project/genereg/ with full documentation.

## Abbreviations

BRCA: Breast Invasive Carcinoma
CHEK1: Checkpoint Kinase 1
DNMT1: DNA Methyltransferase 1
ENCODE: Encyclopedia of DNA Elements
GeCo: Data-Driven Genomic Computing
GENCODE: Encyclopedia of Genes and gene variants
GMQL: GenoMetric Query Language
GRN: Gene regulatory network
MI: Mutual information
NER: Nucleotide excision repair pathway
OLS: Ordinary least squares
OV: Ovarian Serous Cystadenocarcinoma
PDX: Patient-derived xenograft
TCGA: The Cancer Genome Atlas
TF: Transcription factor
TSS: Transcription start site
