# Supplementary material for: Predictive modeling of gene expression regulation
Source: BMC Bioinformatics. 2021 Nov 27;22:571. doi: 10.1186/s12859-021-04481-1 (PMC8626902; doi:10.1186/s12859-021-04481-1)
Supplement: Supplementary file 1 — Additional file 1: S0 List of genes and patient samples considered, S1 Additional aspects of the developed data analysis algorithm and obtained results, S2 Further information about the performed computational evaluation using ARACNe, S3 Gene expression networks generated, S4 Alternative feature selection methods compared. [file 12859_2021_4481_MOESM1_ESM.pdf]

Predictive modeling of gene expression regulation

Chiara Regondi, Maddalena Fratelli, Giovanna Damia, Federica Guffanti, Monica Ganzinelli, Matteo Matteucci, Marco Masseroli.

Supplementary Material

Table of Contents

S0 List of genes and patient samples considered **2**
S0.1 Target genes 2
S0.2 TCGA OV patient samples 6
S0.3 TCGA BRCA basal-like patient Samples 8

S1 Data analysis: Algorithm and results **9**
S1.1 Feature selection 9
S1.2 Linear regression 11
S1.3 Results: Additional details 12
S1.3.1 DNA_REPAIR pathway 13
S1.3.2 STEM_CELLS pathway 15
S1.3.3 GLUCOSE_METABOLISM pathway 17

S2 Computational evaluation: ARACNe **24**

S3 Gene expression networks **27**
S3.1 DNA_REPAIR pathway (M3 models) 27
S3.2 DNA_REPAIR pathway (M5 models) 29
S3.3 STEM_CELLS pathway (M3 models) 30
S3.4 STEM_CELLS pathway (M5 models) 35
S3.5 GLUCOSE_METABOLISM pathway (M3 models) 40
S3.6 GLUCOSE_METABOLISM pathway (M5 models) 43

S4 Alternative feature selection methods **46**
S4.1 Feature selection 46
S4.1.1 Incremental forward feature selection with feature re-evaluation 46
S4.1.2 Incremental forward feature selection with no feature re-evaluation 47
S4.1.3 Forward feature selection on all features 47
S4.1.4 Incremental Lasso feature selection with feature re-evaluation 48
S4.1.5 Lasso feature selection on all features 49
S4.2 Results and comparison 49
S4.2.1 Our incremental FFS method vs. FFS on all features 50
S4.2.2 Our incremental FFS method vs. incremental FFS with no feature re-evaluation 54
S4.2.3 Our incremental FFS method vs. incremental Lasso with feature re-evaluation 58
S4.2.4 Our incremental FFS method vs. Lasso on all features 62
S4.2.5 Lasso on all features vs. FFS on all features 66
S4.2.6 Overall summary comparison of the considered methods 70
S4.2.7 Common features in all 5 feature selection methods 72

S0. List of genes and patient samples considered

Here we provide the lists of the 177 target human genes from 3 biological pathways (DNA_REPAIR, STEM_CELLS, GLUCOSE_METABOLISM) and of the 372 TCGA Ovarian Serous Cystadenocarcinoma (OV) patient samples used in our computational method, as well as of the 122 TCGA Breast Invasive Carcinoma (BRCA) Basal-like patient samples used to validate our results.

S0.1 Target genes

Lists of the used 177 target human genes from the DNA_REPAIR (20 genes), STEM_CELLS (73 genes) and GLUCOSE_METABOLISM (84 genes) biological pathways.

Gene Symbol	Entrez Gene ID	Pathway	Pathway Subclass
BRCA1	672	DNA_REPAIR	Double Strand Breaks (DSB)
CDK12	51755	DNA_REPAIR	Gene CDK12 Group
ERCC1	2067	DNA_REPAIR	Nucleotide Excision Repair (NER)
ERCC2	2068	DNA_REPAIR	Nucleotide Excision Repair (NER)
ERCC4	2072	DNA_REPAIR	Nucleotide Excision Repair (NER)
ERCC5	2073	DNA_REPAIR	Nucleotide Excision Repair (NER)
FANCA	2175	DNA_REPAIR	Double Strand Breaks (DSB)
FANCC	2176	DNA_REPAIR	Double Strand Breaks (DSB)
FANCD2	2177	DNA_REPAIR	Double Strand Breaks (DSB)
FANCF	2188	DNA_REPAIR	Double Strand Breaks (DSB)
MLH1	4292	DNA_REPAIR	Gene MLH1 Group
OGG1	4968	DNA_REPAIR	Base Excision Repair (BER)
PALB2	79728	DNA_REPAIR	Double Strand Breaks (DSB)
PARP1	142	DNA_REPAIR	Base Excision Repair (BER)
POLB	5423	DNA_REPAIR	Base Excision Repair (BER)
POLE	5426	DNA_REPAIR	Unclassified
POLQ	10721	DNA_REPAIR	Double Strand Breaks (DSB)
RAD51	5888	DNA_REPAIR	Double Strand Breaks (DSB)
TP53BP1	7158	DNA_REPAIR	Unclassified
XPA	7507	DNA_REPAIR	Nucleotide Excision Repair (NER)
ABCB5	340273	STEM_CELLS	Cancer Stem Cells Markers
ABCG2	9429	STEM_CELLS	AKT & PI3 Kinase - mTOR Signaling, Cancer Therapeutic Targets
ALCAM	214	STEM_CELLS	Cancer Stem Cells Markers
ALDH1A1	216	STEM_CELLS	Loss of Stemness, Cancer Stem Cells Markers
ATM	472	STEM_CELLS	Cancer Therapeutic Targets
ATXN1	6310	STEM_CELLS	Cancer Stem Cells Markers
AXL	558	STEM_CELLS	Cell Migration & Metastasis, Cancer Therapeutic Targets
BMI1	648	STEM_CELLS	Cancer Stem Cells Markers
BMP7	655	STEM_CELLS	Self-Renewal
CD24	100133941	STEM_CELLS	Cancer Stem Cells Markers
CD34	947	STEM_CELLS	Loss of Stemness, Cancer Stem Cells Markers
CD38	952	STEM_CELLS	Cancer Stem Cells Markers
CD44	960	STEM_CELLS	Cancer Stem Cells Markers

CHEK1	1111	STEM_CELLS	Cancer Therapeutic Targets
DACH1	1602	STEM_CELLS	Loss of Stemness
DDR1	780	STEM_CELLS	Cancer Therapeutic Targets
DKK1	22943	STEM_CELLS	Cancer Therapeutic Targets
DLL1	28514	STEM_CELLS	Notch Signaling
DLL4	54567	STEM_CELLS	Notch Signaling
DNMT1	1786	STEM_CELLS	Self-Renewal
EGF	1950	STEM_CELLS	Cell Proliferation
ENG	2022	STEM_CELLS	Cancer Stem Cells Markers
EPCAM	4072	STEM_CELLS	WNT Signaling, Cancer Therapeutic Targets
ERBB2	2064	STEM_CELLS	Cell Proliferation
ETFA	2108	STEM_CELLS	Cancer Stem Cells Markers
FGFR2	2263	STEM_CELLS	Self-Renewal
FLOT2	2319	STEM_CELLS	Cancer Stem Cells Markers
FOXA2	3170	STEM_CELLS	Loss of Stemness
FOXP1	27086	STEM_CELLS	Asymmetric Division
FZD7	8324	STEM_CELLS	WNT Signaling, Cancer Therapeutic Targets
GATA3	2625	STEM_CELLS	Cancer Stem Cells Markers
GSK3B	2932	STEM_CELLS	Regulation of Glycogen Metabolism, AKT & PI3 Kinase - mTOR Signaling, Cancer Therapeutic Targets
HDAC1	3065	STEM_CELLS	Asymmetric Division
ID1	3397	STEM_CELLS	Cell Migration & Metastasis, Cancer Therapeutic Targets
IKBKB	3551	STEM_CELLS	STAT-NFkB Signaling, Cancer Therapeutic Targets
CXCL8	3576	STEM_CELLS	Cell Migration & Metastasis
ITGA2	3673	STEM_CELLS	Cancer Stem Cells Markers
ITGA4	3676	STEM_CELLS	Cancer Stem Cells Markers
ITGA6	3655	STEM_CELLS	Cancer Stem Cells Markers
ITGB1	3688	STEM_CELLS	Cancer Stem Cells Markers
JAG1	182	STEM_CELLS	Notch Signaling
JAK2	3717	STEM_CELLS	STAT-NFkB Signaling, Cancer Therapeutic Targets
KIT	3815	STEM_CELLS	Cancer Stem Cells Markers
KITLG	4254	STEM_CELLS	Cell Proliferation
KLF17	128209	STEM_CELLS	Cell Migration & Metastasis, Cancer Therapeutic Targets
KLF4	9314	STEM_CELLS	Pluripotency
LATS1	9113	STEM_CELLS	Hippo Signaling
LIN28A	79727	STEM_CELLS	Pluripotency
LIN28B	389421	STEM_CELLS	Cell Proliferation
MAML1	9794	STEM_CELLS	Notch Signaling
MERTK	10461	STEM_CELLS	Hippo Signaling
MS4A1	931	STEM_CELLS	Cancer Stem Cells Markers
MUC1	4582	STEM_CELLS	Cancer Stem Cells Markers
MYC	4609	STEM_CELLS	Pluripotency
MYCN	4613	STEM_CELLS	Asymmetric Division
NANOG	79923	STEM_CELLS	Pluripotency
NFKB1	4790	STEM_CELLS	STAT-NFkB Signaling, Cancer Therapeutic Targets
NOS2	4843	STEM_CELLS	Cell Proliferation

NOTCH1	4851	STEM_CELLS	Notch Signaling
NOTCH2	4853	STEM_CELLS	Notch Signaling
PECAM1	5175	STEM_CELLS	Loss of Stemness, Cancer Stem Cells Markers
PLAT	5327	STEM_CELLS	Cell Migration & Metastasis
PLAUR	5329	STEM_CELLS	Cell Migration & Metastasis
POU5F1	5460	STEM_CELLS	Pluripotency
PROM1	8842	STEM_CELLS	Cancer Stem Cells Markers
PTCH1	5727	STEM_CELLS	Hedgehog Signaling, Loss of Stemness, Cancer Therapeutic Targets
PTPRC	5788	STEM_CELLS	Cancer Stem Cells Markers
SAV1	60485	STEM_CELLS	Hippo Signaling
SIRT1	23411	STEM_CELLS	Asymmetric Division
SMO	6608	STEM_CELLS	Hedgehog Signaling, Cancer Therapeutic Targets
SNAI1	6615	STEM_CELLS	Cell Migration & Metastasis
SOX2	6657	STEM_CELLS	Pluripotency
STAT3	6774	STEM_CELLS	Cancer Therapeutic Targets
ACLY	47	GLUCOSE_METABOLISM	Tricarboxylic Acid (TCA) Cycle
ACO1	48	GLUCOSE_METABOLISM	Tricarboxylic Acid (TCA) Cycle
ACO2	50	GLUCOSE_METABOLISM	Tricarboxylic Acid (TCA) Cycle
AGL	178	GLUCOSE_METABOLISM	Glycogen Degradation
ALDOA	226	GLUCOSE_METABOLISM	Glycolysis
ALDOB	229	GLUCOSE_METABOLISM	Glycolysis
ALDOC	230	GLUCOSE_METABOLISM	Glycolysis
BPGM	669	GLUCOSE_METABOLISM	Glycolysis
CS	1431	GLUCOSE_METABOLISM	Tricarboxylic Acid (TCA) Cycle
DLAT	1737	GLUCOSE_METABOLISM	Tricarboxylic Acid (TCA) Cycle
DLD	1738	GLUCOSE_METABOLISM	Tricarboxylic Acid (TCA) Cycle
DLST	1743	GLUCOSE_METABOLISM	Tricarboxylic Acid (TCA) Cycle
ENO1	2023	GLUCOSE_METABOLISM	Glycolysis
ENO2	2026	GLUCOSE_METABOLISM	Glycolysis
ENO3	2027	GLUCOSE_METABOLISM	Glycolysis
FBP1	2203	GLUCOSE_METABOLISM	Gluconeogenesis
FBP2	8789	GLUCOSE_METABOLISM	Gluconeogenesis
FH	2271	GLUCOSE_METABOLISM	Tricarboxylic Acid (TCA) Cycle
G6PC	2538	GLUCOSE_METABOLISM	Gluconeogenesis
G6PC3	92579	GLUCOSE_METABOLISM	Gluconeogenesis
G6PD	2539	GLUCOSE_METABOLISM	Pentose Phosphate Pathway
GALM	130589	GLUCOSE_METABOLISM	Glycolysis
GBE1	2632	GLUCOSE_METABOLISM	Glycogen Synthesis
GCK	2645	GLUCOSE_METABOLISM	Glycolysis
GPI	2821	GLUCOSE_METABOLISM	Unclassified
GSK3A	2931	GLUCOSE_METABOLISM	Regulation of Glycogen Metabolism
GSK3B	2932	GLUCOSE_METABOLISM	Regulation of Glycogen Metabolism, AKT & PI3 Kinase - mTOR Signaling, Cancer Therapeutic Targets
GYS1	2997	GLUCOSE_METABOLISM	Glycogen Synthesis
GYS2	2998	GLUCOSE_METABOLISM	Glycogen Synthesis
H6PD	9563	GLUCOSE_METABOLISM	Pentose Phosphate Pathway

HK2	3099	GLUCOSE_METABOLISM	Unclassified
HK3	3101	GLUCOSE_METABOLISM	Unclassified
IDH1	3417	GLUCOSE_METABOLISM	Tricarboxylic Acid (TCA) Cycle
IDH2	3418	GLUCOSE_METABOLISM	Tricarboxylic Acid (TCA) Cycle
IDH3A	3419	GLUCOSE_METABOLISM	Tricarboxylic Acid (TCA) Cycle
IDH3B	3420	GLUCOSE_METABOLISM	Tricarboxylic Acid (TCA) Cycle
IDH3G	3421	GLUCOSE_METABOLISM	Tricarboxylic Acid (TCA) Cycle
MDH1	4190	GLUCOSE_METABOLISM	Tricarboxylic Acid (TCA) Cycle
MDH1B	130752	GLUCOSE_METABOLISM	Tricarboxylic Acid (TCA) Cycle
MDH2	4191	GLUCOSE_METABOLISM	Tricarboxylic Acid (TCA) Cycle
OGDH	4967	GLUCOSE_METABOLISM	Tricarboxylic Acid (TCA) Cycle
PC	5091	GLUCOSE_METABOLISM	Gluconeogenesis, Tricarboxylic Acid (TCA) Cycle
PCK1	5105	GLUCOSE_METABOLISM	Gluconeogenesis, Tricarboxylic Acid (TCA) Cycle
PCK2	5106	GLUCOSE_METABOLISM	Gluconeogenesis, Tricarboxylic Acid (TCA) Cycle
PDHA1	5160	GLUCOSE_METABOLISM	Tricarboxylic Acid (TCA) Cycle
PDHB	5162	GLUCOSE_METABOLISM	Tricarboxylic Acid (TCA) Cycle
PDK1	5163	GLUCOSE_METABOLISM	Regulation of Glucose Metabolism
PDK2	5164	GLUCOSE_METABOLISM	Regulation of Glucose Metabolism
PDK3	5165	GLUCOSE_METABOLISM	Regulation of Glucose Metabolism
PDK4	5166	GLUCOSE_METABOLISM	Regulation of Glucose Metabolism
PDP2	57546	GLUCOSE_METABOLISM	Regulation of Glucose Metabolism
PDPR	55066	GLUCOSE_METABOLISM	Regulation of Glucose Metabolism
PFKL	5211	GLUCOSE_METABOLISM	Unclassified
PGAM2	5224	GLUCOSE_METABOLISM	Unclassified
PGK1	5230	GLUCOSE_METABOLISM	Glycolysis
PGK2	5232	GLUCOSE_METABOLISM	Glycolysis
PGLS	25796	GLUCOSE_METABOLISM	Pentose Phosphate Pathway
PGM1	5236	GLUCOSE_METABOLISM	Glycogen Degradation
PGM2	55276	GLUCOSE_METABOLISM	Glycogen Degradation
PGM3	5238	GLUCOSE_METABOLISM	Glycogen Degradation
PHKA1	5255	GLUCOSE_METABOLISM	Regulation of Glycogen Metabolism
PHKB	5257	GLUCOSE_METABOLISM	Regulation of Glycogen Metabolism
PHKG1	5260	GLUCOSE_METABOLISM	Regulation of Glycogen Metabolism
PHKG2	5261	GLUCOSE_METABOLISM	Regulation of Glycogen Metabolism
PKLR	5313	GLUCOSE_METABOLISM	Unclassified
PRPS1	5631	GLUCOSE_METABOLISM	Pentose Phosphate Pathway
PRPS1L1	221823	GLUCOSE_METABOLISM	Pentose Phosphate Pathway
PRPS2	5634	GLUCOSE_METABOLISM	Pentose Phosphate Pathway
PYGL	5836	GLUCOSE_METABOLISM	Glycogen Degradation
PYGM	5837	GLUCOSE_METABOLISM	Glycogen Degradation
RBKS	64080	GLUCOSE_METABOLISM	Pentose Phosphate Pathway
RPE	6120	GLUCOSE_METABOLISM	Pentose Phosphate Pathway
RPIA	22934	GLUCOSE_METABOLISM	Pentose Phosphate Pathway
SDHA	6389	GLUCOSE_METABOLISM	Tricarboxylic Acid (TCA) Cycle
SDHB	6390	GLUCOSE_METABOLISM	Tricarboxylic Acid (TCA) Cycle
SDHC	6391	GLUCOSE_METABOLISM	Tricarboxylic Acid (TCA) Cycle

SDHD	6392	GLUCOSE_METABOLISM	Tricarboxylic Acid (TCA) Cycle
SUCLA2	8803	GLUCOSE_METABOLISM	Tricarboxylic Acid (TCA) Cycle
SUCLG1	8802	GLUCOSE_METABOLISM	Tricarboxylic Acid (TCA) Cycle
SUCLG2	8801	GLUCOSE_METABOLISM	Tricarboxylic Acid (TCA) Cycle
TALDO1	6888	GLUCOSE_METABOLISM	Pentose Phosphate Pathway
TKT	7086	GLUCOSE_METABOLISM	Pentose Phosphate Pathway
TPI1	7167	GLUCOSE_METABOLISM	Glycolysis
UGP2	7360	GLUCOSE_METABOLISM	Glycogen Synthesis

S0.2 TCGA OV patient samples

List of the 372 TCGA Ovarian Serous Cystadenocarcinoma patient samples used.

TCGA-04-1331-01A	TCGA-13-1483-01A	TCGA-24-1563-01A	TCGA-29-1766-01A
TCGA-04-1332-01A	TCGA-13-1485-01A	TCGA-24-1565-01A	TCGA-29-1768-01A
TCGA-04-1338-01A	TCGA-13-1487-01A	TCGA-24-1567-01A	TCGA-29-1769-01A
TCGA-04-1341-01A	TCGA-13-1488-01A	TCGA-24-1603-01A	TCGA-29-1770-01A
TCGA-04-1343-01A	TCGA-13-1489-01A	TCGA-24-1604-01A	TCGA-29-1774-01A
TCGA-04-1347-01A	TCGA-13-1489-02A	TCGA-24-1616-01A	TCGA-29-1776-01A
TCGA-04-1350-01A	TCGA-13-1492-01A	TCGA-24-1842-01A	TCGA-29-1777-01A
TCGA-04-1356-01A	TCGA-13-1495-01A	TCGA-24-1843-01A	TCGA-29-1778-01A
TCGA-04-1357-01A	TCGA-13-1496-01A	TCGA-24-1844-01A	TCGA-29-1781-01A
TCGA-04-1361-01A	TCGA-13-1497-01A	TCGA-24-1845-01A	TCGA-29-1783-01A
TCGA-04-1362-01A	TCGA-13-1498-01A	TCGA-24-1846-01A	TCGA-29-1784-01A
TCGA-04-1364-01A	TCGA-13-1499-01A	TCGA-24-1847-01A	TCGA-29-1785-01A
TCGA-04-1365-01A	TCGA-13-1501-01A	TCGA-24-1850-01A	TCGA-29-2414-01A
TCGA-04-1514-01A	TCGA-13-1505-01A	TCGA-24-1923-01A	TCGA-29-2414-02A
TCGA-04-1519-01A	TCGA-13-1506-01A	TCGA-24-1924-01A	TCGA-29-2425-01A
TCGA-04-1530-01A	TCGA-13-1507-01A	TCGA-24-1928-01A	TCGA-29-2427-01A
TCGA-04-1536-01A	TCGA-13-1509-01A	TCGA-24-1930-01A	TCGA-29-2428-01A
TCGA-04-1542-01A	TCGA-13-1510-01A	TCGA-24-2020-01A	TCGA-30-1714-01A
TCGA-04-1648-01A	TCGA-13-1511-01A	TCGA-24-2023-01A	TCGA-30-1718-01A
TCGA-04-1651-01A	TCGA-13-1512-01A	TCGA-24-2024-01A	TCGA-30-1853-01A
TCGA-04-1655-01A	TCGA-13-2060-01A	TCGA-24-2026-01A	TCGA-30-1857-01A
TCGA-09-0364-01A	TCGA-20-0987-01A	TCGA-24-2027-01A	TCGA-30-1860-01A
TCGA-09-0366-01A	TCGA-20-0991-01A	TCGA-24-2033-01A	TCGA-30-1861-01A
TCGA-09-0367-01A	TCGA-20-1682-01A	TCGA-24-2035-01A	TCGA-30-1862-01A
TCGA-09-0369-01A	TCGA-20-1683-01A	TCGA-24-2036-01A	TCGA-30-1866-01A
TCGA-09-1659-01B	TCGA-20-1686-01A	TCGA-24-2038-01A	TCGA-30-1891-01A
TCGA-09-1661-01B	TCGA-20-1687-01A	TCGA-24-2254-01A	TCGA-30-1892-01A
TCGA-09-1662-01A	TCGA-23-1021-01B	TCGA-24-2261-01A	TCGA-31-1944-01A
TCGA-09-1665-01B	TCGA-23-1022-01A	TCGA-24-2262-01A	TCGA-31-1946-01A
TCGA-09-1666-01A	TCGA-23-1023-01A	TCGA-24-2267-01A	TCGA-31-1950-01A
TCGA-09-1667-01C	TCGA-23-1024-01A	TCGA-24-2271-01A	TCGA-31-1951-01A
TCGA-09-1668-01B	TCGA-23-1026-01B	TCGA-24-2280-01A	TCGA-31-1953-01A
TCGA-09-1669-01A	TCGA-23-1027-01A	TCGA-24-2281-01A	TCGA-31-1956-01A
TCGA-09-1670-01A	TCGA-23-1028-01A	TCGA-24-2288-01A	TCGA-31-1959-01A
TCGA-09-1673-01A	TCGA-23-1029-01B	TCGA-24-2289-01A	TCGA-36-1568-01A
TCGA-09-2044-01B	TCGA-23-1030-01A	TCGA-24-2290-01A	TCGA-36-1569-01A

TCGA-09-2045-01A	TCGA-23-1107-01A	TCGA-24-2293-01A	TCGA-36-1570-01A
TCGA-09-2048-01A	TCGA-23-1109-01A	TCGA-24-2297-01A	TCGA-36-1571-01A
TCGA-09-2051-01A	TCGA-23-1110-01A	TCGA-24-2298-01A	TCGA-36-1574-01A
TCGA-09-2053-01C	TCGA-23-1111-01A	TCGA-25-1312-01A	TCGA-36-1576-01A
TCGA-09-2054-01A	TCGA-23-1113-01A	TCGA-25-1313-01A	TCGA-36-1577-01A
TCGA-09-2056-01B	TCGA-23-1114-01B	TCGA-25-1315-01A	TCGA-36-1580-01A
TCGA-10-0927-01A	TCGA-23-1116-01A	TCGA-25-1316-01A	TCGA-36-1581-01A
TCGA-10-0928-01A	TCGA-23-1118-01A	TCGA-25-1317-01A	TCGA-57-1582-01A
TCGA-10-0931-01A	TCGA-23-1119-01A	TCGA-25-1318-01A	TCGA-57-1583-01A
TCGA-10-0933-01A	TCGA-23-1120-01A	TCGA-25-1319-01A	TCGA-57-1584-01A
TCGA-10-0936-01A	TCGA-23-1122-01A	TCGA-25-1320-01A	TCGA-57-1585-01A
TCGA-10-0937-01A	TCGA-23-1123-01A	TCGA-25-1321-01A	TCGA-57-1586-01A
TCGA-10-0938-01A	TCGA-23-1809-01A	TCGA-25-1322-01A	TCGA-57-1993-01A
TCGA-13-0714-01A	TCGA-23-2077-01A	TCGA-25-1323-01A	TCGA-57-1994-01A
TCGA-13-0720-01A	TCGA-23-2078-01A	TCGA-25-1326-01A	TCGA-59-2348-01A
TCGA-13-0724-01A	TCGA-23-2084-01A	TCGA-25-1328-01A	TCGA-59-2350-01A
TCGA-13-0725-01A	TCGA-24-0966-01A	TCGA-25-1329-01A	TCGA-59-2351-01A
TCGA-13-0726-01A	TCGA-24-0968-01A	TCGA-25-1623-01A	TCGA-59-2352-01A
TCGA-13-0727-01A	TCGA-24-0970-01B	TCGA-25-1626-01A	TCGA-59-2354-01A
TCGA-13-0730-01A	TCGA-24-0979-01A	TCGA-25-1627-01A	TCGA-59-2355-01A
TCGA-13-0762-01A	TCGA-24-0982-01A	TCGA-25-1628-01A	TCGA-59-2363-01A
TCGA-13-0765-01A	TCGA-24-1103-01A	TCGA-25-1630-01A	TCGA-61-1721-01A
TCGA-13-0766-01A	TCGA-24-1104-01A	TCGA-25-1631-01A	TCGA-61-1724-01A
TCGA-13-0768-01A	TCGA-24-1105-01A	TCGA-25-1632-01A	TCGA-61-1725-01A
TCGA-13-0795-01A	TCGA-24-1413-01A	TCGA-25-1633-01A	TCGA-61-1728-01A
TCGA-13-0797-01A	TCGA-24-1416-01A	TCGA-25-1634-01A	TCGA-61-1733-01A
TCGA-13-0800-01A	TCGA-24-1417-01A	TCGA-25-1635-01A	TCGA-61-1736-01B
TCGA-13-0804-01A	TCGA-24-1418-01A	TCGA-25-1870-01A	TCGA-61-1737-01A
TCGA-13-0883-01A	TCGA-24-1419-01A	TCGA-25-1877-01A	TCGA-61-1738-01A
TCGA-13-0884-01B	TCGA-24-1422-01A	TCGA-25-2042-01A	TCGA-61-1741-01A
TCGA-13-0885-01A	TCGA-24-1423-01A	TCGA-25-2391-01A	TCGA-61-1900-01A
TCGA-13-0886-01A	TCGA-24-1424-01A	TCGA-25-2392-01A	TCGA-61-1907-01A
TCGA-13-0887-01A	TCGA-24-1425-01A	TCGA-25-2393-01A	TCGA-61-1910-01A
TCGA-13-0888-01A	TCGA-24-1426-01A	TCGA-25-2396-01A	TCGA-61-1911-01A
TCGA-13-0891-01A	TCGA-24-1427-01A	TCGA-25-2398-01A	TCGA-61-1914-01A
TCGA-13-0893-01B	TCGA-24-1428-01A	TCGA-25-2399-01A	TCGA-61-1918-01A
TCGA-13-0897-01A	TCGA-24-1430-01A	TCGA-25-2400-01A	TCGA-61-1919-01A
TCGA-13-0900-01B	TCGA-24-1431-01A	TCGA-25-2401-01A	TCGA-61-1995-01A
TCGA-13-0901-01B	TCGA-24-1434-01A	TCGA-25-2404-01A	TCGA-61-1998-01A
TCGA-13-0905-01B	TCGA-24-1435-01A	TCGA-25-2409-01A	TCGA-61-2000-01A
TCGA-13-0906-01A	TCGA-24-1464-01A	TCGA-29-1688-01A	TCGA-61-2002-01A
TCGA-13-0908-01B	TCGA-24-1467-01A	TCGA-29-1690-01A	TCGA-61-2003-01A
TCGA-13-0911-01A	TCGA-24-1469-01A	TCGA-29-1691-01A	TCGA-61-2008-01A
TCGA-13-0913-02A	TCGA-24-1470-01A	TCGA-29-1693-01A	TCGA-61-2008-02A
TCGA-13-0916-01A	TCGA-24-1471-01A	TCGA-29-1694-01A	TCGA-61-2009-01A
TCGA-13-0920-01A	TCGA-24-1474-01A	TCGA-29-1695-01A	TCGA-61-2012-01A
TCGA-13-0923-01A	TCGA-24-1544-01A	TCGA-29-1696-01A	TCGA-61-2088-01A
TCGA-13-0924-01A	TCGA-24-1546-01A	TCGA-29-1697-01A	TCGA-61-2092-01A
TCGA-13-1403-01A	TCGA-24-1549-01A	TCGA-29-1701-01A	TCGA-61-2097-01A

TCGA-13-1404-01A	TCGA-24-1550-01A	TCGA-29-1703-01A	TCGA-61-2098-01A
TCGA-13-1405-01A	TCGA-24-1551-01A	TCGA-29-1705-01A	TCGA-61-2101-01A
TCGA-13-1407-01A	TCGA-24-1552-01A	TCGA-29-1707-02A	TCGA-61-2102-01A
TCGA-13-1408-01A	TCGA-24-1553-01A	TCGA-29-1710-01A	TCGA-61-2104-01A
TCGA-13-1409-01A	TCGA-24-1557-01A	TCGA-29-1711-01A	TCGA-61-2109-01A
TCGA-13-1410-01A	TCGA-24-1558-01A	TCGA-29-1761-01A	TCGA-61-2110-01A
TCGA-13-1411-01A	TCGA-24-1560-01A	TCGA-29-1762-01A	TCGA-61-2111-01A
TCGA-13-1477-01A	TCGA-24-1562-01A	TCGA-29-1763-01A	TCGA-61-2113-01A

S0.3 TCGA BRCA basal-like patient samples

List of the 122 TCGA Breast Invasive Carcinoma patient samples of Basal-like subtype, according to the PAM50 molecular subtype classification, used for validation.

TCGA-A1-A0SK-01A	TCGA-AC-A8OQ-01A	TCGA-BH-A0B3-01A	TCGA-E2-A574-01A
TCGA-A1-A0SO-01A	TCGA-AN-A0XU-01A	TCGA-BH-A0B9-01A	TCGA-E9-A1N8-01A
TCGA-A1-A0SP-01A	TCGA-AO-A0JL-01A	TCGA-BH-A0E0-01A	TCGA-E9-A1NC-01A
TCGA-A2-A0SX-01A	TCGA-AO-A124-01A	TCGA-BH-A0RX-01A	TCGA-E9-A1ND-01A
TCGA-A2-A0T0-01A	TCGA-AO-A128-01A	TCGA-BH-A0WA-01A	TCGA-E9-A22G-01A
TCGA-A2-A0YE-01A	TCGA-AO-A129-01A	TCGA-BH-A1F0-01A	TCGA-E9-A243-01A
TCGA-A2-A0YJ-01A	TCGA-AO-A1KR-01A	TCGA-BH-A1F6-01A	TCGA-E9-A244-01A
TCGA-A2-A0YM-01A	TCGA-AQ-A54N-01A	TCGA-BH-A1FC-01A	TCGA-E9-A3QA-01A
TCGA-A2-A1G1-01A	TCGA-AR-A0TP-01A	TCGA-BH-A5IZ-01A	TCGA-E9-A5FL-01A
TCGA-A2-A25F-01A	TCGA-AR-A0TU-01A	TCGA-BH-A6R9-01A	TCGA-EW-A1OW-01A
TCGA-A2-A3XS-01A	TCGA-AR-A0U0-01A	TCGA-C8-A1HJ-01A	TCGA-EW-A1P4-01A
TCGA-A2-A3XU-01A	TCGA-AR-A0U4-01A	TCGA-C8-A27B-01A	TCGA-EW-A1P8-01A
TCGA-A2-A3XX-01A	TCGA-AR-A1AI-01A	TCGA-D8-A1JK-01A	TCGA-EW-A1PB-01A
TCGA-A2-A3XY-01A	TCGA-AR-A1AJ-01A	TCGA-D8-A1JL-01A	TCGA-EW-A1PH-01A
TCGA-A2-A4RX-01A	TCGA-AR-A1AQ-01A	TCGA-D8-A1JM-01A	TCGA-EW-A3U0-01A
TCGA-A7-A13D-01A	TCGA-AR-A1AR-01A	TCGA-D8-A1XK-01A	TCGA-EW-A6SB-01A
TCGA-A7-A13E-01A	TCGA-AR-A1AY-01A	TCGA-D8-A1XQ-01A	TCGA-GM-A2DF-01A
TCGA-A7-A26F-01B	TCGA-AR-A24Q-01A	TCGA-D8-A27F-01A	TCGA-GM-A3XL-01A
TCGA-A7-A26G-01A	TCGA-AR-A251-01A	TCGA-D8-A27H-01A	TCGA-LL-A441-01A
TCGA-A7-A4SD-01A	TCGA-AR-A256-01A	TCGA-D8-A27M-01A	TCGA-LL-A5YP-01A
TCGA-A7-A4SE-01A	TCGA-AR-A2LR-01A	TCGA-E2-A14N-01A	TCGA-LL-A6FR-01A
TCGA-A7-A5ZV-01A	TCGA-AR-A5QQ-01A	TCGA-E2-A1AZ-01A	TCGA-LL-A8F5-01A
TCGA-A7-A6VV-01A	TCGA-B6-A0I1-01A	TCGA-E2-A1B6-01A	TCGA-OL-A5D7-01A
TCGA-A7-A6VW-01A	TCGA-B6-A0RE-01A	TCGA-E2-A1II-01A	TCGA-OL-A5RW-01A
TCGA-A7-A6VY-01A	TCGA-B6-A0RT-01A	TCGA-E2-A1LG-01A	TCGA-OL-A5S0-01A
TCGA-AC-A2BK-01A	TCGA-B6-A0RU-01A	TCGA-E2-A1LH-01A	TCGA-OL-A66I-01A
TCGA-AC-A2QH-01B	TCGA-B6-A0X1-01A	TCGA-E2-A1LI-01A	TCGA-OL-A6VO-01A
TCGA-AC-A2QJ-01A	TCGA-B6-A1KF-01A	TCGA-E2-A1LK-01A	TCGA-S3-AA0Z-01A
TCGA-AC-A62X-01A	TCGA-B6-A400-01A	TCGA-E2-A1LL-01A	TCGA-S3-AA10-01A
TCGA-AC-A6IW-01A	TCGA-B6-A402-01A	TCGA-E2-A573-01A	TCGA-S3-AA15-01A
TCGA-AC-A7VC-01A	TCGA-B6-A409-01A		

S1. Data analysis: Algorithm and results

The proposed algorithm and its main operations for extracting and analyzing data for each target gene are summarized in Figure S1.1. After the initial data extraction and preparation, described in the main paper, the data analysis part is composed of two subsequent main phases: Feature selection and then linear regression, detailed as follows.

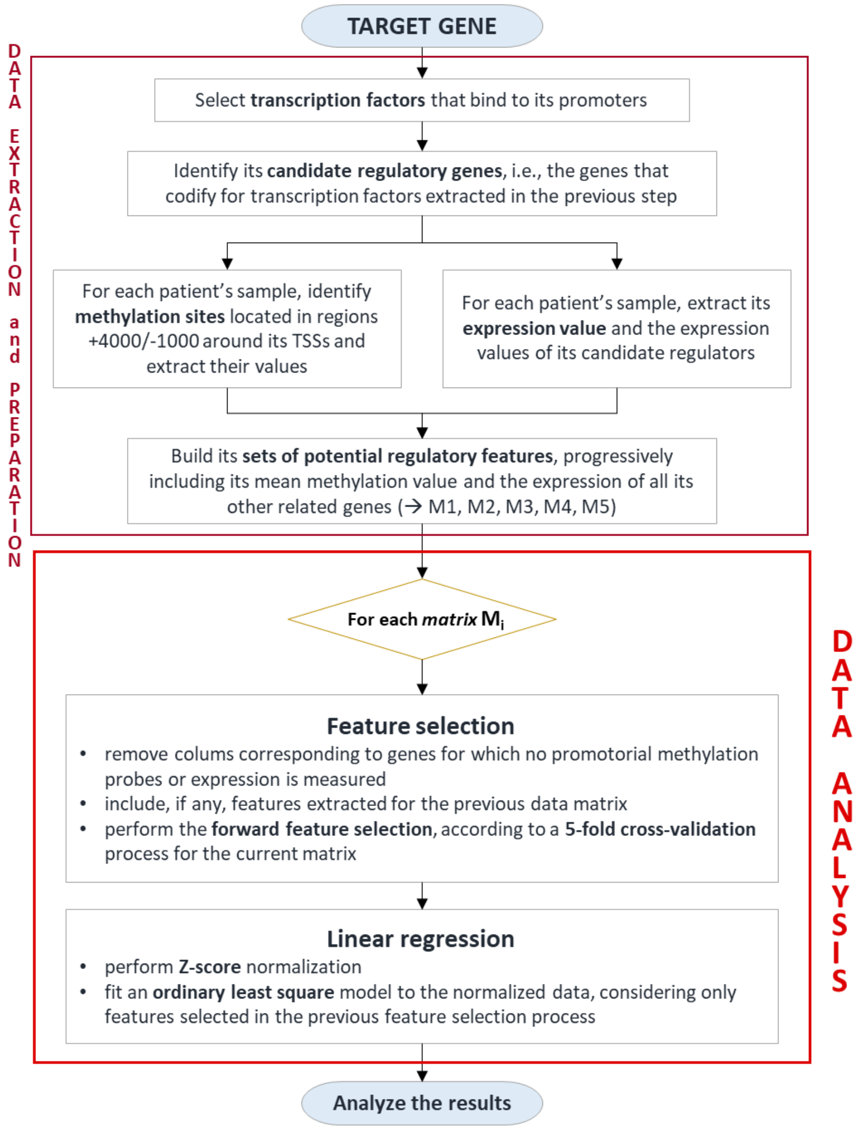

Figure S1.1: Operations performed on each gene of interest.

S1.1 Feature selection

As described in the main paper, the first data analysis phase for each target gene consists in selecting its best subset of features for the subsequent regression modeling task, avoiding to consider the ones found non-relevant to model the regulation of the expression of the target gene. This feature selection process allows to perform a more accurate analysis for each target gene in a shorter time, reducing the dimension of the set of features with respect to the set of observations; considering the whole set of possible features, in fact, would highly increase the computational complexity and would result in an underdetermined regression problem. The feature selection process (Figure S1.2(a) and Figure S1.2(b)) iterates along the list of target genes belonging

to the gene set selected for the analysis, and for each of such genes it executes the following operations for each of the data matrices considered:

a. The current gene data matrix M_i is imported and all its empty columns are removed, since they do not contribute to the regression model (e.g., columns with all *NaN* methylation values, corresponding to genes for which no promoter methylation probes are measured in TCGA, or columns regarding genes whose expression is zero in the considered TCGA samples);

b. All those features belonging to the previous evaluated set of features (i.e., data matrix), if any, of the same gene that were not selected by the previous feature selection process, if any, are removed. We assume those same features could not be selected in the current feature selection process, since they have already been discarded once. The features retrieved from the previous selection, along with the new features in the current data matrix, are involved in the new feature selection process; this means that features previously selected may be discarded as a result of the current feature selection, because some of the new added features may better explain the expression of the target gene;

c. On such features of the current data matrix M_i, a forward feature selection is first performed by evaluating the linear regression performance of growing subsets of these features: starting from a feature, all the other features are added one by one according to the one providing the best regression performance on a training set of samples; then, all defined subsets of features are evaluated on a testing set of different samples, and the subset with the best cross-validation regression performance is retained. In order to reduce the possible bias induced by the particular patient samples considered for the training and test sets, we randomly split the available TCGA aliquots into five, possibly equal, subgroups of samples. This partition is performed only once at the beginning of the data analysis, and then the same five subgroups of samples are used for all the feature selections of all target genes. Thus, for each target gene and each of its data matrices considered, we execute the feature selection five times, using each time four of the sample subgroups as training set and the fifth remaining subgroup as testing set, according to a k-fold cross-validation process with k = 5. Finally, the intersection of all the features extracted in the five testing sets is computed to obtain the final selected features (i.e., those extracted in all five subgroups) for the current target gene in the current data matrix. Figure S1.2(a) and Figure S1.2(b) illustrate this feature selection procedure.

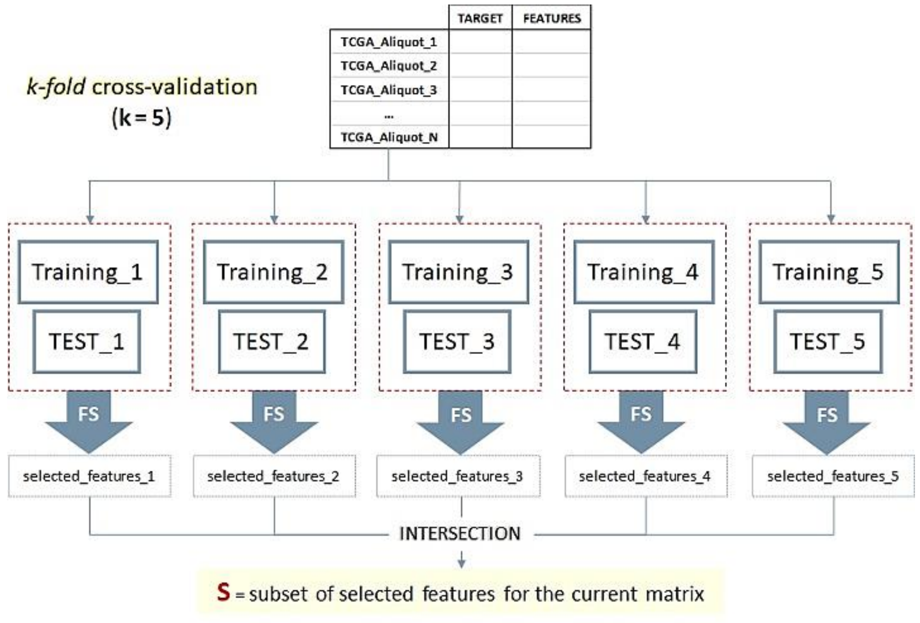

Figure S1.2(a): Feature selection procedure: k-fold cross-validation for each data matrix.

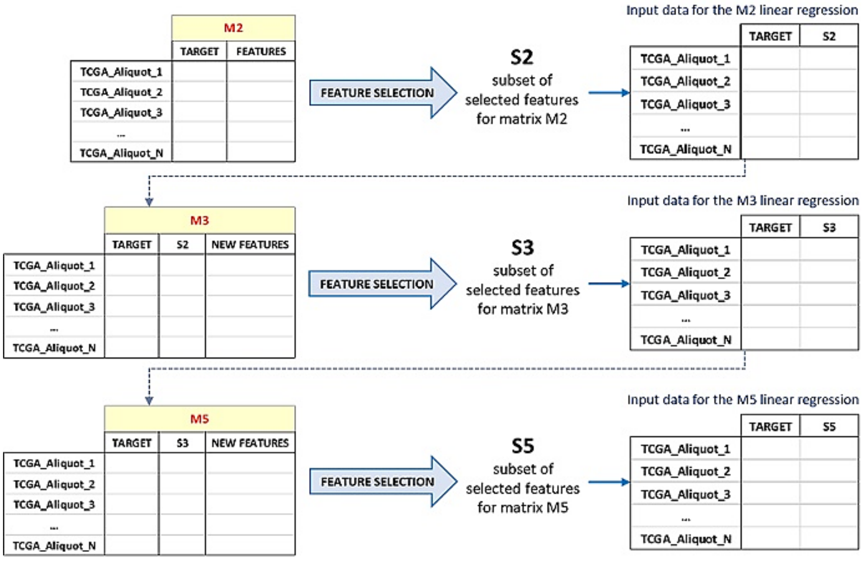

Figure S1.2(b): Feature selection procedure: subsequently evaluated data matrices for each target gene.

S1.2 Linear regression

The second phase of our data analysis consists in fitting a linear model on each individual target gene and on the set of features selected in the previous phase for each of its data matrices, with the aim of extracting the most significant features of each data matrix (i.e., feature type) that better explain the target gene expression (i.e., output variable), quantifying their effect.

Just as done for the feature selection, the process iterates along the list of target genes belonging to the gene set under analysis and their considered data matrices M_i, fitting a linear model as follows:

a. First, data are normalized to allow comparisons of results; since heterogeneous data are used for assessing the regulation of the target gene expression, normalization is needed for consistently comparing results not only within but also across the different individual regression models defined. The Z-score normalization is applied to convert the values of each considered variable (i.e., feature or target gene expression) to have a distribution with `mean_value = 0` and `variance = 1`, using the following formula:

$$\text{Normalized(v)} = \frac{\text{v - mean\_value}}{\text{std\_dev}}$$

This normalization allows comparing the regression coefficients assigned to the different features within the same or in different models and establishing which one has the highest impact on the output variable;

b. Then, we build the best linear regression model by fitting an ordinary least squares (OLS) model to the normalized data.

For each target gene, from each data matrix we extract and evaluate only the relevant features for the regulation of the target gene expression, according to the values of their confidence intervals. A confidence interval represents an interval estimate that is supposed to contain the true value of an unknown parameter. It has an associated confidence level (i.e., 95%) quantifying the probability that the parameter value (in our case, the estimated regression coefficient) lies in the interval. So, only if the effect of a feature is very unlikely to be zero (i.e., its 95% confidence interval does not contain the zero value), then the feature is considered relevant for the model. This means that the actual set of relevant features predicted involved in the regulation of the expression of the target gene is smaller than, or equal to, the set of features selected by the previous feature selection process and used as input for the regression model. Furthermore, the *coefficient of determination* (R^2

score) is calculated to measure the regression fit quality. Figure S1.3 shows how this identification of relevant features works, by considering as an example the gene TKT and the features selected from its data matrix M3.

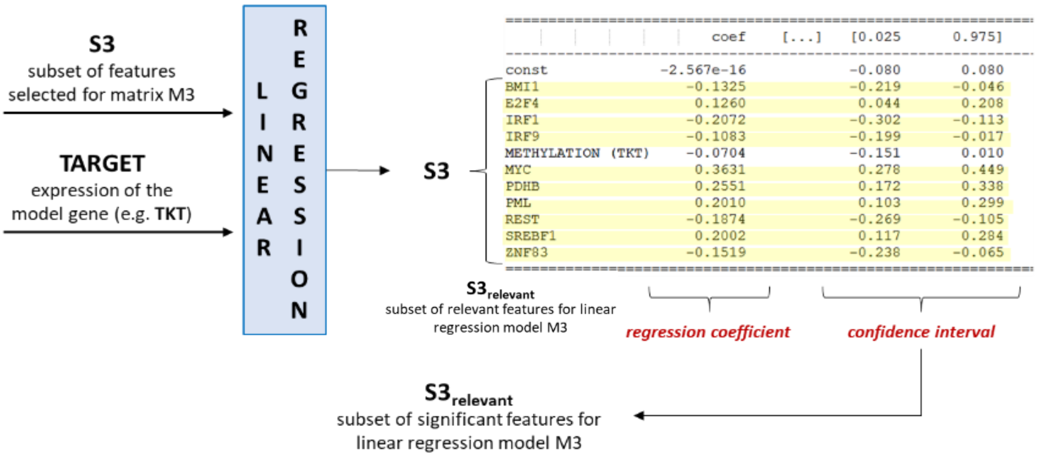

$S3_{relevant}$	Coefficient	Feature Description
MYC	0.3631	Candidate regulatory gene of the model gene TKT
PDHB	0.2551	Gene of the GLUCOSE_METABOLISM pathway
PML	0.2010	Candidate regulatory gene of the GLUCOSE_METABOLISM pathway
SREBF1	0.2002	Candidate regulatory gene of the model gene TKT
E2F4	0.1260	Candidate regulatory gene of the GLUCOSE_METABOLISM pathway
IRF9	-0.1083	Candidate regulatory gene of the model gene TKT
BMI1	-0.1325	Candidate regulatory gene of the GLUCOSE_METABOLISM pathway
ZNF83	-0.1519	Candidate regulatory gene of the GLUCOSE_METABOLISM pathway
REST	-0.1874	Candidate regulatory gene of the model gene TKT
IRF1	-0.2072	Candidate regulatory gene of the GLUCOSE_METABOLISM pathway

Figure S1.3: Example of linear regression for matrix M3 of gene TKT and identification of relevant features.

Finally, all the results (i.e., target genes and their predicted regulatory features) are graphically represented through expression networks, one for each evaluated target gene set and feature set type (i.e., data matrix), grouping target genes in subclasses according to their function inside their gene set.
In our specific application case, we considered 3 sets of target genes, each grouping the genes known to compose one of the pathways more relevant in Ovarian Serous Cystadenocarcinoma (OV), as follows:

- DNA_REPAIR pathway genes (genomic instability due to DNA damages is one of the main causes of different types of cancer, including ovarian tumor);
- STEM_CELLS pathway genes (tumor stem cells represent one of the main tumor relapsing mechanisms, regulating tumor's either sensitive or resistant response to chemotherapeutic drugs);
- GLUCOSE_METABOLISM pathway genes (there is a strict correlation between glucose addiction and cancer platinum-based therapy, and the resistance of some cancer cells to this type of therapy may be associated with an alteration in their metabolic profile).

The common color code shared by the drawn networks allows an easy interpretation of the results and the assessment of the effect of both methylation and transcription factors, as well as of the interrelationships between pathways of interest (i.e., how each target gene is regulated and how in turn it participates, if so, in regulating other target genes). *Section S3* of this document illustrates all the resulting gene expression networks for OV cancer analysis.

S1.3 Results: Additional details

Here, we report additional details of the main results obtained for each evaluated target gene set (i.e., pathway gene set) and feature set type (i.e., data matrix) model.

S1.3.1 DNA_REPAIR pathway

Model M2: the expression of each target gene is considered regulated by genes in the DNA_REPAIR pathway and genes encoding for transcription factors binding to the target gene promoters.
The Adjusted R^2 score resulted higher than 0.6 only for 3 genes of the pathway (POLE, TP53BP1 and FANCD2), reaching the maximum value of 0.67 for the gene POLE.
For 6 genes of the pathway, the gene methylation is selected as one of the relevant features involved in the regulation of their expression:

- BRCA1, with regression coefficient -0.3940;
- CDK12, with regression coefficient -0.1907;
- ERCC1, with regression coefficient -0.1214;
- ERCC4, with regression coefficient -0.2574;
- ERCC5, with regression coefficient -0.1430.
- FANCF, with regression coefficient -0.2287;

There are 4 most frequent regulators, each selected as a relevant regulatory feature for 4 DNA repair genes:

- POLQ (gene of the DNA_REPAIR pathway) is selected as:
  - 1st (out of 6 features) for gene FANCD2, with regression coefficient 0.5649;
  - 2nd (out of 4 features) for gene BRCA1, with regression coefficient 0.3057;
  - 5th (out of 5 features) for gene MLH1, with regression coefficient -0.1146;
  - 10th (out of 10 features) for gene OGG1, with regression coefficient -0.3507;

- POLE (gene of the DNA_REPAIR pathway) is selected as:
  - 1st (out of 5 features) for gene FANCA, with regression coefficient 0.5217;
  - 2nd (out of 5 features) for gene FANCC, with regression coefficient 0.2999;
  - 2nd (out of 8 features) for gene FANCF, with regression coefficient 0.2506;
  - 6th (out of 6 features) for gene XPA, with regression coefficient -0.2565;

- FANCD2 (gene of the DNA_REPAIR pathway) is selected as:
  - 1st (out of 5 features) for gene MLH1, with regression coefficient 0.4074;
  - 1st (out of 10 features) for gene OGG1, with regression coefficient 0.4532;
  - 1st (out of 4 features) for gene POLQ, with regression coefficient 0.6733;
  - 1st (out of 8 features) for gene RAD51, with regression coefficient 0.4551;

- HCFC1 (candidate regulatory gene of the DNA_REPAIR pathway) is selected as:
  - 1st (out of 6 features) for gene PARP1, with regression coefficient 0.3347;
  - 2nd (out of 10 features) for gene POLE, with regression coefficient 0.1900;
  - 2nd (out of 9 features) for gene ERCC2, with regression coefficient 0.3464;
  - 11th (out of 11 features) for gene PALB2, with regression coefficient -0.1770.

Model M3: the expression of each target gene is considered regulated by genes in the DNA_REPAIR pathway and genes encoding for transcription factors binding to the target gene promoters or to the promoters of other genes in the DNA_REPAIR pathway.
The Adjusted R^2 score resulted higher than 0.6 for 6 genes of the pathway (FANCC, TP53BP1, ERCC2, FANCD2, POLQ and BRCA1), reaching the maximum value of 0.64 for the gene FANCC.

For 5 genes of the pathway, the gene methylation is selected as one of the relevant features involved in the regulation of their expression:

- BRCA1, with regression coefficient -0.3442;
- ERCC1, with regression coefficient -0.1347;
- ERCC4, with regression coefficient -0.1817;
- ERCC5, with regression coefficient -0.1630.
- FANCF, with regression coefficient -0.1314;

Differently from the previous model M2, gene CDK12 loses its methylation from the set of relevant regulatory features, because more relevant regulators are found among the set of candidate regulatory genes of the pathway, including the selected regulatory genes SUZ12, BCLAF1, RUNX1 and ZNF217.

There are 4 most frequent regulators, each selected as a relevant regulatory feature for 4 DNA repair genes:

- POLQ (gene of the DNA_REPAIR pathway) is selected as:
  - 1st (out of 7 features) for gene FANCD2, with regression coefficient 0.5386;
  - 2nd (out of 8 features) for gene BRCA1, with regression coefficient 0.2603;
  - 7th (out of 9 features) for gene MLH1, with regression coefficient -0.2060;
  - 9th (out of 9 features) for gene OGG1, with regression coefficient -0.4165;

- FANCD2 (gene of the DNA_REPAIR pathway) is selected as:
  - 1st (out of 8 features) for gene POLQ, with regression coefficient 0.4926;
  - 1st (out of 9 features) for gene OGG1, with regression coefficient 0.4220;
  - 2nd (out of 9 features) for gene MLH1, with regression coefficient 0.2916;
  - 2nd (out of 11 features) for gene RAD51, with regression coefficient 0.3216;

- SUZ12 (candidate regulatory gene of the DNA_REPAIR pathway) is selected as:
  - 1st (out of 5 features) for gene CDK12, with regression coefficient 0.3550;
  - 2nd (out of 8 features) for gene BRCA1, with regression coefficient 0.1691;
  - 3rd (out of 9 features) for gene PALB2, with regression coefficient 0.1983;
  - 6th (out of 9 features) for gene OGG1, with regression coefficient 0.1212;

- ZHX1 (candidate regulatory gene of the DNA_REPAIR pathway) is selected as:
  - 2nd (out of1 features) for gene XPA, with regression coefficient 0.2350;
  - 3rd (out of 7 features) for gene ERCC5, with regression coefficient 0.1853;
  - 7th (out of 11 features) for gene FANCF, with regression coefficient 0.1212;
  - 11th (out of 13 features) for gene ERCC1, with regression coefficient -0.1759.

<u>Model M5</u>: the expression of each target gene is considered regulated by genes in the DNA_REPAIR pathway, genes encoding for transcription factors binding to the target gene promoters or to the promoters of other genes in the DNA_REPAIR pathway, genes in the STEM_CELLS or GLUCOSE_METABOLISM pathway and their regulatory genes.
The Adjusted R^2 score resulted higher than 0.6 for 8 genes of the pathway (POLB, FANCC, POLQ, TP53BP1, FANCD2, ERCC2, POLE and ERCC1), reaching the maximum value of 0.68 for gene POLB.
For 4 genes of the pathway the gene methylation is selected as one of the relevant features involved in the regulation of their expression:

- BRCA1, with regression coefficient -0.3475;
- ERCC4, with regression coefficient -0.2608;
- ERCC5, with regression coefficient -0.1774.
- FANCF, with regression coefficient -0.1659;

Differently from the previous model M3, gene ERCC1 loses its methylation from the set of its relevant regulatory features, because more relevant regulators are found among the set of candidate regulatory genes of the pathway, including the selected regulatory genes HNRNPUL1 and NCOA3 of the STEM_CELLS pathway.

There is one single most frequent regulator, selected as a relevant regulatory feature for 5 DNA repair genes:

➢ XRCC3 (candidate regulatory gene of the STEM_CELLS pathway) is selected as:
  - 2nd (out of 11 features) for gene POLQ, with regression coefficient 0.1916;
  - 4th (out of 9 features) for gene POLE, with regression coefficient 0.1625;
  - 5th (out of 11 features) for gene POLE, with regression coefficient 0.1098;
  - 9th (out of 11 features) for gene FANCC, with regression coefficient 0.0985;
  - 9th (out of 12 features) for gene PARP1, with regression coefficient -0.0767;

Figure S1.4 at the end of the section displays the 8 genes of the DNA_REPAIR pathway that overall show the best linear fit (Adjusted $R^2 > 0.6$) in model M5 and the set of their relevant regulatory features.

S1.3.2 STEM_CELLS pathway

<u>Model M2</u>: the expression of each target gene is considered regulated by genes in the STEM_CELLS pathway and genes encoding for transcription factors binding to the target gene promoters.
The Adjusted R^2 score resulted higher than 0.6 for 5 genes of the pathway (PTPRC, ITGA4, PECAM1, ENG and LATS1), reaching the maximum value of 0.77 for the gene PTPRC.
For 10 genes of the pathway the gene methylation is selected as one of the relevant features involved in the regulation of their expression:

- ATM, with regression coefficient -0.2246;
- CD34, with regression coefficient -0.1010;
- CHEK1, with regression coefficient -0.1121;
- CXCL8, with regression coefficient -0.1422;
- ENG, with regression coefficient -0.0896;
- EPCAM, with regression coefficient -0.3359;
- MAML1, with regression coefficient -0.1017;
- PLAT, with regression coefficient -0.2656;
- POU5F1, with regression coefficient -0.2565;
- SAV1, with regression coefficient -0.2558.

Only one single most frequent regulator is selected as a relevant regulatory feature for 9 stem cells target genes:
➢ PTPRC (gene of the STEM_CELLS pathway) is selected as:
  - 1st (out of 4 feature) for gene CD38, with regression coefficient 0.4178;
  - 1st (out of 6 features) for gene CD44, with regression coefficient 0.3582;
  - 1st (out of 5 features) for gene ITGA4, with regression coefficient 0.5299;

- 1st (out of 10 features) for gene JAK2, with regression coefficient 0.3359;
- 1st (out of 4 features) for gene MS4A1, with regression coefficient 0.4193;
- 1st (out of 5 features) for gene PECAM1, with regression coefficient 0.4741;
- 3rd (out of 5 features) for gene AXL, with regression coefficient 0.1786;
- 6th (out of 6 features) for gene DLL4, with regression coefficient -0.2629;
- 7th (out of 8 features) for gene SMO, with regression coefficient -0.2176.

Model M3: the expression of each target gene is considered regulated by genes in the STEM_CELLS pathway and genes encoding for transcription factors binding to the target gene promoters or to the promoters of other genes in the STEM_CELLS pathway.
The Adjusted R^2 score resulted higher than 0.6 for 11 genes of the pathway (PTPRC, ITGA4, LATS1, DNMT1, JAK2, NOTCH2, MAML1, PECAM1, CHEK1, AXL and ENG), reaching the maximum value of 0.82 for the gene PTPRC.
For 10 genes of the pathway the gene methylation is selected as one of the relevant features involved in the regulation of their expression:

- ATM, with regression coefficient -0.1591;
- CD34, with regression coefficient -0.1733;
- CHEK1, with regression coefficient -0.0960;
- CXCL8, with regression coefficient -0.1628;
- DACH1, with regression coefficient -0.1215;
- EPCAM, with regression coefficient -0.2276;
- MAML1, with regression coefficient -0.0723;
- PLAT, with regression coefficient -0.2482;
- POU5F1, with regression coefficient -0.1984;
- SAV1, with regression coefficient -0.1725.

Differently from the previous model M2, methylation becomes relevant for gene DACH1, while gene ENG loses its methylation from the set of its relevant regulatory features, because more relevant regulators are found among the set of candidate regulatory genes of the pathway, in particular the selected regulatory genes TEAD2 and ZNF143 of the STEM_CELLS pathway.

Only one single most frequent regulator is selected as a relevant regulatory feature for 10 stem cells target genes:

➢ PAX8 (candidate regulatory gene of the STEM_CELLS pathway) is selected as:
- 1st (out of 1 feature) for gene NOS2, with regression coefficient -0.1792;
- 2nd (out of 8 features) for gene BMP7, with regression coefficient 0.2424;
- 2nd (out of 3 features) for gene NANOG, with regression coefficient -0.1257;
- 6th (out of 7 features) for gene LIN28B, with regression coefficient -0.1251;
- 6th (out of 6 features) for gene MYCN, with regression coefficient -0.1533;
- 7th (out of 7 features) for gene KLF4, with regression coefficient -0.1542;
- 8th (out of 8 features) for gene CD44, with regression coefficient -0.1997;
- 10th (out of 12 features) for gene MYC, with regression coefficient -0.1491;
- 11th (out of 13 features) for gene ETFA, with regression coefficient -0.1424;
- 11th (out of 11 features) for gene ITGA4, with regression coefficient -0.1075;

Model M5: the expression of each target gene is considered regulated by genes in the STEM_CELLS pathway, genes encoding for transcription factors binding to the target gene promoters or to the promoters of other genes in the STEM_CELLS pathway, genes in the DNA_REPAIR or GLUCOSE_METABOLISM pathway and their regulatory genes.
The Adjusted R^2 score resulted higher than 0.6 for the same 11 genes of the pathway as in model M3 (PTPRC, ITGA4, DNMT1, LATS1, MAML1, JAK2, CHEK1, PECAM1, NOTCH2, AXL and ENG), reaching the maximum value of 0.82 for the gene PTPRC.
For 9 genes of the pathway, the gene methylation is selected as one of the relevant features involved in the regulation of their expression, i.e., the same as in M3, with the exception of gene DACH1:

- ATM, with regression coefficient -0.1591;
- CD34, with regression coefficient -0.1733;
- CHEK1, with regression coefficient -0.0960;
- CXCL8, with regression coefficient -0.1628;
- EPCAM, with regression coefficient -0.2276;
- MAML1, with regression coefficient -0.0723;
- PLAT, with regression coefficient -0.2482;
- POU5F1, with regression coefficient -0.1984;
- SAV1, with regression coefficient -0.1725.

The same single most frequent regulator is selected as a relevant regulatory feature for the same 10 stem cells target genes as in model M3:

➢ PAX8 (candidate regulatory gene of the STEM_CELLS pathway) is selected as:
- 2nd (out of 2 features) for gene NOS2, with regression coefficient -0.1858;
- 2nd (out of 8 features) for gene BMP7, with regression coefficient 0.2264;
- 2nd (out of 3 features) for gene NANOG, with regression coefficient -0.1257;
- 6th (out of 9 features) for gene LIN28B, with regression coefficient -0.1277;
- 6th (out of 7 features) for gene MYCN, with regression coefficient -0.1625;
- 8th (out of 8 features) for gene CD44, with regression coefficient -0.1997;
- 9th (out of 9 features) for gene KLF4, with regression coefficient -0.1344;
- 10th (out of 12 features) for gene ETFA, with regression coefficient -0.1213;
- 11th (out of 13 features) for gene MYC, with regression coefficient -0.1132;
- 12th (out of 12 features) for gene ITGA4, with regression coefficient -0.0921;

Figure S1.5 at the end of the section displays the 11 genes of the STEM_CELLS pathway that overall show the best linear fit (Adjusted $R^2 > 0.6$) in model M5 and the set of their relevant regulatory features.

S1.3.3 GLUCOSE_METABOLISM pathway

Model M2: the expression of each target gene is considered regulated by genes in GLUCOSE_METABOLISM pathway and genes encoding for transcription factors binding to the target gene promoters.
The Adjusted R^2 score resulted higher than 0.6 only for 3 genes of the pathway (SDHD, DLAT and ACO2), reaching the maximum value of 0.78 for the gene SDHD.
For 14 genes of the pathway, the gene methylation is selected as one of the relevant features involved in the regulation of their expression:

- AGL, with regression coefficient -0.2198;

- ALDOC, with regression coefficient -0.3634;
- DLD, with regression coefficient -0.2418;
- IDH3A, with regression coefficient -0.1198;
- IDH3B, with regression coefficient -0.2608;
- MDH2, with regression coefficient -0.1573;
- PCK1, with regression coefficient -0.3248;
- PCK2, with regression coefficient -0.1278;
- PDHA1, with regression coefficient -0.1490;
- PDK4, with regression coefficient -0.1237;
- PGM3, with regression coefficient -0.2025;
- PYGM, with regression coefficient -0.1122;
- RPE, with regression coefficient -0.1445;
- SDHA, with regression coefficient -0.2296.

There are 2 most frequent regulators, each selected as a relevant regulatory feature for 10 glucose metabolism target genes:

➢ SUCLG1 (gene of the GLUCOSE_METABOLISM pathway) is selected as:
  - 1st (out of 10 features) for gene FH, with regression coefficient 0.1944;
  - 1st (out of 8 features) for gene PDK2, with regression coefficient 0.2395;
  - 1st (out of 10 features) for gene SDHC, with regression coefficient 0.3659;
  - 2nd (out of 5 features) for gene RPIA, with regression coefficient 0.2701;
  - 2nd (out of 8 features) for gene SDHD, with regression coefficient 0.1968;
  - 3rd (out of 10 features) for gene SDHB, with regression coefficient 0.1796;
  - 3rd (out of 5 features) for gene SUCLA2, with regression coefficient 0.1769;
  - 4th (out of 11 features) for gene MDH1, with regression coefficient 0.2055;
  - 5th (out of 13 features) for gene ACO2, with regression coefficient 0.1116;
  - 7th (out of 7 features) for gene ENO2, with regression coefficient -0.3067;

➢ PHKB (gene of the GLUCOSE_METABOLISM pathway) is selected as:
  - 2nd (out of 3 features) for gene PDP2, with regression coefficient 0.1832;
  - 2nd (out of 9 features) for gene SUCLG2, with regression coefficient 0.2254;
  - 3rd (out of 10 features) for gene SDHC, with regression coefficient 0.2183;
  - 4th (out of 6 features) for gene H6PD, with regression coefficient 0.2405;
  - 4th (out of 4 features) for gene PDPR, with regression coefficient 0.0921;
  - 4th (out of 5 features) for gene PKLR, with regression coefficient -0.1542;
  - 5th (out of 9 features) for gene IDH3A, with regression coefficient 0.0977;
  - 6th (out of 11 features) for gene MDH1, with regression coefficient -0.0955;
  - 6th (out of 9 features) for gene PHKG1, with regression coefficient -0.0912;
  - 10th (out of 10 features) for gene PDHB, with regression coefficient -0.1710;

<u>Model M3</u>: the expression of each target gene is considered regulated by genes in GLUCOSE_METABOLISM pathway and genes encoding for transcription factors binding to the target gene promoters or to the promoters of other genes in the GLUCOSE_METABOLISM pathway.
The Adjusted R^2 score resulted higher than 0.6 for 6 genes of the pathway (SDHD, TPI1, DLAT, MDH1, ACO2, and PRPS1), reaching the maximum value of 0.78 for the gene SDHD.

For 12 genes of the pathway, the gene methylation is selected as one of the relevant features involved in the regulation of their expression:

- AGL, with regression coefficient of -0.0917;
- ALDOC, with regression coefficient -0.3614;
- DLD, with a regression coefficient -0.2463;
- IDH3B, with regression coefficient -0.2262;
- MDH2, with regression coefficient -0.1419;
- PCK1, with regression coefficient -0.3192;
- PDK3, with regression coefficient -0.1905;
- PDK4, with regression coefficient -0.1139;
- PGM3, with regression coefficient -0.1658;
- PYGM, with regression coefficient -0.1122;
- RPE, with regression coefficient -0.1595;
- SDHA, with regression coefficient -0.2319.

There is one single most frequent regulator, selected as a relevant regulatory feature for 8 glucose metabolism target genes:

➢ ILK (candidate regulatory gene of the GLUCOSE_METABOLISM pathway) is selected as:
  - 1st (out of 4 features) for gene ALDOC, with regression coefficient 0.2099;
  - 2nd (out of 8 features) for gene TALDO1, with regression coefficient 0.2258;
  - 3rd (out of 7 features) for gene DLST, with regression coefficient 0.1962;
  - 5th (out of 7 features) for gene ACO2, with regression coefficient 0.1340;
  - 5th (out of 11 features) for gene ENO1, with regression coefficient 0.1271;
  - 5th (out of 9 features) for gene SUCLG1, with regression coefficient 0.1253;
  - 6th (out of 7 features) for gene DLAT, with regression coefficient 0.1025;
  - 6th (out of 7 features) for gene RPIA, with regression coefficient -0.1845.

<u>Model M5</u>: the expression of each target gene is considered regulated by genes in GLUCOSE_METABOLISM pathway, genes encoding for transcription factors binding to the target gene promoters or to the promoters of other genes in the GLUCOSE_METABOLISM pathway, genes in the DNA_REPAIR or STEM_CELLS pathway and their regulatory genes.
The Adjusted R^2 score resulted higher than 0.6 for 9 genes of the pathway (SDHD, TPI1, DLAT, HK3, ACO2, ACLY, PRPS1, PHKA1 and ALDOA), reaching the maximum value of 0.79 for the gene SDHD.
For 12 genes of the pathway, the gene methylation is selected as one of the relevant features involved in the regulation of their expression (the same genes as in model M3, with the exception of gene PDK4 and the addition of gene TKT):

- AGL, with regression coefficient -0.0896;
- ALDOC, with regression coefficient -0.3334;
- DLD, with regression coefficient -0.2559;
- IDH3B, with regression coefficient -0.2132;
- MDH2, with regression coefficient -0.1513;
- PCK1, with regression coefficient -0.3547;
- PDK3, with regression coefficient -0.2408;
- PGM3, with regression coefficient -0.1134;
- PYGM, with regression coefficient -0.1141;

- RPE, with regression coefficient -0.1718;
- SDHA, with regression coefficient -0.2191;
- TKT, with regression coefficient -0.0819.

There is one single most frequent regulator, selected as a relevant regulatory feature for 10 glucose metabolism target genes:

➢ TSC22D4 (candidate regulatory gene of the STEM_CELLS pathway) is selected as:
- 1st (out of 5 features) for gene PCK1, with regression coefficient 0.1532;
- 4th (out of 9 features) for gene MDH2, with regression coefficient 0.1929;
- 5th (out of 8 features) for gene PFKL, with regression coefficient 0.2117;
- 6th (out of 12 features) for gene BPGM, with regression coefficient 0.1340;
- 6th (out of 10 features) for gene H6PD, with regression coefficient -0.1458;
- 7th (out of 13 features) for gene CS, with regression coefficient -0.0804;
- 8th (out of 12 features) for gene SUCLG2, with regression coefficient 0.1094;
- 9th (out of 9 features) for gene AGL, with regression coefficient -0.1076;
- 12th (out of 12 features) for gene ENO, with regression coefficient -0.1792;
- 13th (out of 13 features) for gene PGM3, with regression coefficient -0.2090;

Figure S1.6 at the end of the section displays the 9 genes of the GLUCOSE_METABOLISM pathway that overall show the best linear fit (Adjusted $R^2 > 0.6$) in model M5 and the set of their relevant regulatory features.

GENE	Relevant Features	Adjusted R^2	Regression Coefficient	Feature Description
**POLB**	THAP1	**0.68**	0.5420	Candidate regulatory gene of the DNA_REPAIR pathway
	SRSF7		0.2957	Candidate regulatory gene of the genes STEM_CELLS pathway and GLUCOSE_METABOLISM pathways
	IKBKB		0.2249	Gene of the STEM_CELLS pathway
	ZNF207		0.1338	Candidate regulatory gene of the STEM_CELLS pathway
	XRCC3		0.1098	Candidate regulatory gene of the STEM_CELLS pathway
	ITGB1		0.0837	Gene of the STEM_CELLS pathway
	MCM3		-0.0770	Candidate regulatory gene of the STEM_CELLS pathway
	PTCH1		-0.1124	Gene of the STEM_CELLS pathway
	SRSF1		-0.1350	Candidate regulatory gene of the genes STEM_CELLS pathway and GLUCOSE_METABOLISM pathways
	ELF1		-0.1629	Candidate regulatory gene of the DNA_REPAIR pathway
	ZBTB1		-0.1823	Candidate regulatory gene of the STEM_CELLS pathway
**FANCC**	XPA	**0.67**	0.3552	Gene of the DNA_REPAIR pathway
	POLE		0.2505	Gene of the DNA_REPAIR pathway
	MCM7		0.1869	Candidate regulatory gene of the STEM_CELLS pathway
	BRCA1		0.1826	Gene of the DNA_REPAIR pathway
	ZBTB5		0.1681	Candidate regulatory gene of the DNA_REPAIR pathway
	EPCAM		0.1157	Gene of the STEM_CELLS pathway
	ITGB1		0.1146	Gene of the STEM_CELLS pathway
	THRA		0.1119	Candidate regulatory gene of the genes STEM_CELLS pathway and GLUCOSE_METABOLISM pathways
	XRCC3		0.0985	Candidate regulatory gene of the STEM_CELLS pathway
	SOX2		0.0624	Gene of the STEM_CELLS pathway
	SIRT1		-0.1191	Gene of the STEM_CELLS pathway
**POLQ**	FANCD2	**0.66**	0.4779	Gene of the DNA_REPAIR pathway
	XRCC3		0.1916	Candidate regulatory gene of the STEM_CELLS pathway
	FOXM1		0.1758	Candidate regulatory gene of the DNA_REPAIR pathway
	ZBTB11		0.1705	Candidate regulatory gene of the DNA_REPAIR pathway
	GSK3B		0.1606	Gene of the STEM_CELLS pathway
	TRIM22		0.1342	Candidate regulatory gene of the DNA_REPAIR pathway
	HLTF		0.1330	Candidate regulatory gene of the DNA_REPAIR pathway
	TAF15		0.1038	Candidate regulatory gene of the DNA_REPAIR pathway
	ITGA2		-0.0789	Gene of the STEM_CELLS pathway
	GATAD2B		-0.1087	Candidate regulatory gene of the DNA_REPAIR pathway
	OGG1		-0.2126	Gene of the DNA_REPAIR pathway
**TP53BP1**	ZSCAN29	**0.65**	0.4646	Candidate regulatory gene of the model gene TP53BP1
	ZBTB40		0.3737	Candidate regulatory gene of the model gene TP53BP1
	MAML1		0.1917	Gene of the STEM_CELLS pathway
**FANCD2**	POLQ	**0.63**	0.5799	Gene of the DNA_REPAIR pathway
	NR2C2		0.2748	Candidate regulatory gene of the DNA_REPAIR pathway
	OGG1		0.2547	Gene of the DNA_REPAIR pathway
	MLH1		0.1692	Gene of the DNA_REPAIR pathway
	TAF15		-0.0866	Candidate regulatory gene of the DNA_REPAIR pathway
	ZNF354B		-0.1129	Candidate regulatory gene of the genes STEM_CELLS pathway and GLUCOSE_METABOLISM pathways
	GSK3B		-0.1506	Gene of the STEM_CELLS pathway
**ERCC2**	ERCC1	**0.63**	0.5332	Gene of the DNA_REPAIR pathway
	RCOR1		0.2446	Candidate regulatory gene of the DNA_REPAIR pathway
	PTBP1		0.2316	Candidate regulatory gene of the DNA_REPAIR pathway
	ENG		0.2164	Gene of the STEM_CELLS pathway
	TEAD2		0.2141	Candidate regulatory gene of the DNA_REPAIR pathway
	UBTF		0.1809	Candidate regulatory gene of the DNA_REPAIR pathway
	NFXL1		0.1035	Candidate regulatory gene of the genes STEM_CELLS pathway and GLUCOSE_METABOLISM pathways
	ZZZ3		0.0809	Candidate regulatory gene of the DNA_REPAIR pathway
	ATXN1		-0.0745	Gene of the STEM_CELLS pathway
	NFKB1		-0.1099	Gene of the STEM_CELLS pathway
	MXI1		-0.1113	Candidate regulatory gene of the DNA_REPAIR pathway
	ABCG2		-0.1216	Gene of the STEM_CELLS pathway
	E2F1		-0.1785	Candidate regulatory gene of the DNA_REPAIR pathway
**POLE**	FANCA	**0.62**	0.3124	Gene of the DNA_REPAIR pathway
	NRF1		0.1990	Candidate regulatory gene of the model gene POLE
	FANCF		0.1868	Gene of the DNA_REPAIR pathway
	XRCC3		0.1625	Candidate regulatory gene of the STEM_CELLS pathway
	MCM3		0.1516	Candidate regulatory gene of the STEM_CELLS pathway
	NR2C1		0.1389	Candidate regulatory gene of the DNA_REPAIR pathway
	KITLG		0.1032	Gene of the STEM_CELLS pathway
	MCM7		0.0880	Candidate regulatory gene of the STEM_CELLS pathway
	RNF2		-0.1134	Candidate regulatory gene of the DNA_REPAIR pathway
**ERCC1**	ERCC2	**0.60**	0.5420	Gene of the DNA_REPAIR pathway
	HNRNPUL1		0.2743	Candidate regulatory gene of the STEM_CELLS pathway
	PTTG1		0.1799	Candidate regulatory gene of the DNA_REPAIR pathway
	NCOA3		-0.0821	Candidate regulatory gene of the genes STEM_CELLS pathway and GLUCOSE_METABOLISM pathways
	ZHX1		-0.1278	Candidate regulatory gene of the model gene ERCC1
	E2F4		-0.1561	Candidate regulatory gene of the DNA_REPAIR pathway
	POLR2A		-0.1605	Candidate regulatory gene of the model gene ERCC1
	RCOR1		-0.1712	Candidate regulatory gene of the model gene ERCC1
	SKIL		-0.1874	Candidate regulatory gene of the model gene ERCC1
	PTBP1		-0.2344	Candidate regulatory gene of the model gene ERCC1

Figure S1.4: Model M5 best DNA_REPAIR genes and their regulatory features.

GENE	Relevant Features	Adjusted R^2	Regression Coefficient	Feature Description
**PTPRC**	IKZF1	**0.82**	0.5857	Candidate regulatory gene of the STEM_CELLS pathway
	ITGA4		0.2392	Gene of the STEM_CELLS pathway
	CD38		0.1394	Gene of the STEM_CELLS pathway
	CD44		0.1217	Gene of the STEM_CELLS pathway
	AXL		0.0729	Gene of the STEM_CELLS pathway
	ERCC2		-0.1169	Gene of the DNA_REPAIR pathway
	ELF4		-0.1252	Candidate regulatory gene of the STEM_CELLS pathway
**ITGA4**	PTPRC	**0.76**	0.4511	Gene of the STEM_CELLS pathway
	AXL		0.2539	Gene of the STEM_CELLS pathway
	MEF2A		0.2216	Candidate regulatory gene of the STEM_CELLS pathway
	ERCC2		0.1325	Gene of the DNA_REPAIR pathway
	MERTK		0.1047	Gene of the STEM_CELLS pathway
	ZZZ3		0.0966	Candidate regulatory gene of the STEM_CELLS pathway
	MLH1		0.0961	Gene of the DNA_REPAIR pathway
	SMAD1		0.0924	Candidate regulatory gene of the STEM_CELLS pathway
	SKIL		-0.0715	Candidate regulatory gene of the STEM_CELLS pathway
	XPA		-0.0735	Gene of the DNA_REPAIR pathway
	NR2F2		-0.0804	Candidate regulatory gene of the STEM_CELLS pathway
	PAX8		-0.0921	Candidate regulatory gene of the STEM_CELLS pathway
	SOX6		-0.1051	Candidate regulatory gene of the STEM_CELLS pathway
**DNMT1**	SMARCA4	**0.70**	0.5505	Candidate regulatory gene of the STEM_CELLS pathway
	POLE		0.1738	Gene of the DNA_REPAIR pathway
	CHEK1		0.1565	Gene of the STEM_CELLS pathway
	CC2D1A		0.1552	Candidate regulatory gene of the STEM_CELLS pathway
	MTA2		0.1208	Candidate regulatory gene of the STEM_CELLS pathway
	MCM3		0.1049	Candidate regulatory gene of the STEM_CELLS pathway
	DEAF1		-0.1112	Candidate regulatory gene of the STEM_CELLS pathway
	TEAD2		-0.1282	Candidate regulatory gene of the STEM_CELLS pathway
**LATS1**	ARID1B	**0.70**	0.5248	Candidate regulatory gene of the model gene LATS1
	BCLAF1		0.3338	Candidate regulatory gene of the STEM_CELLS pathway
	CHAMP1		0.1451	Candidate regulatory gene of the STEM_CELLS pathway
	HDAC2		0.1291	Candidate regulatory gene of the model gene LATS1
	TRIM22		0.1166	Candidate regulatory gene of the STEM_CELLS pathway
	POLQ		0.0719	Gene of the DNA_REPAIR pathway
	NR2F1		0.0711	Candidate regulatory gene of the STEM_CELLS pathway
	SMARCB1		-0.0996	Candidate regulatory gene of the STEM_CELLS pathway
	KHSRP		-0.1135	Candidate regulatory gene of the STEM_CELLS pathway
	ELF4		-0.1137	Candidate regulatory gene of the STEM_CELLS pathway
	HNRNPUL1		-0.1191	Candidate regulatory gene of the STEM_CELLS pathway
**MAML1**	ZNF354B	**0.69**	0.4257	Candidate regulatory gene of the STEM_CELLS pathway
	HCFC1		0.2320	Candidate regulatory gene of the model gene MAML1
	TP53BP1		0.2072	Gene of the DNA_REPAIR pathway
	HSF1		0.1889	Candidate regulatory gene of the STEM_CELLS pathway
	ELF1		0.1887	Candidate regulatory gene of the model gene MAML1
	THRAP3		0.1558	Candidate regulatory gene of the STEM_CELLS pathway
	HNRNPUL1		0.1388	Candidate regulatory gene of the STEM_CELLS pathway
	PTTG1		0.1324	Candidate regulatory gene of the STEM_CELLS pathway
	CDK12		0.0778	Gene of the DNA_REPAIR pathway
	ETHYLATION (MAML		-0.0637	Methylation of the model gene MAML1
	XPA		-0.0784	Gene of the DNA_REPAIR pathway
	ATF1		-0.1106	Candidate regulatory gene of the model gene MAML1
	XRCC3		-0.1177	Candidate regulatory gene of the STEM_CELLS pathway
**JAK2**	TRIM22	**0.68**	0.3314	Candidate regulatory gene of the STEM_CELLS pathway
	PTPRC		0.3111	Gene of the STEM_CELLS pathway
	IRF1		0.2624	Candidate regulatory gene of the STEM_CELLS pathway
	NFIB		0.1920	Candidate regulatory gene of the STEM_CELLS pathway
	DDX20		0.1904	Candidate regulatory gene of the STEM_CELLS pathway
	ZBTB5		0.1630	Candidate regulatory gene of the STEM_CELLS pathway
	NCOA1		0.1382	Candidate regulatory gene of the STEM_CELLS pathway
	ZBTB33		0.0966	Candidate regulatory gene of the STEM_CELLS pathway
	FOXM1		-0.0827	Candidate regulatory gene of the STEM_CELLS pathway
	RELA		-0.1076	Candidate regulatory gene of the STEM_CELLS pathway
	ZNF143		-0.1216	Candidate regulatory gene of the STEM_CELLS pathway
**CHEK1**	NFRKB	**0.66**	0.4343	Candidate regulatory gene of the model gene CHEK1
	RAD51		0.2605	Candidate regulatory gene of the STEM_CELLS pathway
	FANCA		0.2058	Gene of the DNA_REPAIR pathway
	CBX5		0.1290	Candidate regulatory gene of the STEM_CELLS pathway
	SRSF1		0.1290	Candidate regulatory gene of the STEM_CELLS pathway
	DACH1		0.1073	Gene of the STEM_CELLS pathway
	YBX3		0.0818	Candidate regulatory gene of the DNA_REPAIR and GLUCOSE_METABOLISM pathways
	IETHYLATION (CHEK1		-0.1144	Methylation of the model gene CHEK1
	MAFK		-0.1611	Candidate regulatory gene of the STEM_CELLS pathway
	ZNF384		-0.1680	Candidate regulatory gene of the STEM_CELLS pathway
	RB1		-0.1680	Candidate regulatory gene of the model gene CHEK1
	ZKSCAN1		-0.1872	Candidate regulatory gene of the STEM_CELLS pathway
**PECAM1**	PTPRC	**0.64**	0.3596	Gene of the STEM_CELLS pathway
	DLL4		0.3124	Gene of the STEM_CELLS pathway
	ZEB2		0.2640	Candidate regulatory gene of the STEM_CELLS pathway
	RUNX1		0.1312	Candidate regulatory gene of the STEM_CELLS pathway
	ITGB1		0.1059	Gene of the STEM_CELLS pathway
**NOTCH2**	CSDE1	**0.64**	0.7441	Candidate regulatory gene of the STEM_CELLS pathway
	SUZ12		0.1462	Candidate regulatory gene of the STEM_CELLS pathway
	YBX3		-0.1356	Candidate regulatory gene of the DNA_REPAIR and GLUCOSE_METABOLISM pathways
**AXL**	ITGA4	**0.63**	0.3422	Gene of the STEM_CELLS pathway
	PTRF		0.3323	Candidate regulatory gene of the STEM_CELLS pathway
	PTPRC		0.2642	Gene of the STEM_CELLS pathway
	HNRNPUL1		0.1932	Candidate regulatory gene of the STEM_CELLS pathway
	LEF1		0.1771	Candidate regulatory gene of the model gene AXL
	OGG1		0.0750	Gene of the DNA_REPAIR pathway
	ETFA		-0.1134	Gene of the STEM_CELLS pathway
	ETV6		-0.1290	Candidate regulatory gene of the STEM_CELLS pathway
**ENG**	ZEB2	**0.62**	0.4504	Candidate regulatory gene of the model gene ENG
	DLL4		0.3729	Gene of the STEM_CELLS pathway
	MEF2D		0.2125	Candidate regulatory gene of the model gene ENG
	ZNF143		-0.1086	Candidate regulatory gene of the STEM_CELLS pathway

Figure S1.5: Model M5 best STEM_CELLS genes and their regulatory features.

GENE	Relevant Features	Adjusted R^2	Regression Coefficient	Feature Description
**SDHD**	DLAT	**0.79**	0.7345	Gene of the GLUCOSE_METABOLISM pathway
	SUCLG1		0.1897	Gene of the GLUCOSE_METABOLISM pathway
	HCFC1		-0.0735	Candidate regulatory gene of the model gene SDHD
	MERTK		-0.0849	Gene of the STEM_CELLS pathway
	MAZ		-0.1266	Candidate regulatory gene of the GLUCOSE_METABOLISM pathway
	TP53BP1		-0.1406	Gene of the DNA_REPAIR pathway
	ACLY		-0.1521	Gene of the GLUCOSE_METABOLISM pathway
**TPI1**	ENO2	**0.76**	0.2825	Gene of the GLUCOSE_METABOLISM pathway
	PHB2		0.2656	Candidate regulatory gene of the GLUCOSE_METABOLISM pathway
	ZNF384		0.2360	Candidate regulatory gene of the GLUCOSE_METABOLISM pathway
	PGM1		0.2176	Gene of the GLUCOSE_METABOLISM pathway
	YBX3		0.1689	Candidate regulatory gene of the GLUCOSE_METABOLISM pathway
	PTTG1		0.1673	Candidate regulatory gene of the GLUCOSE_METABOLISM pathway
	ETFA		0.1334	Gene of the STEM_CELLS pathway
	CREB3		0.1176	Candidate regulatory gene of the GLUCOSE_METABOLISM pathway
	FOXP1		-0.0982	Gene of the STEM_CELLS pathway
	NR2C2		-0.1001	Candidate regulatory gene of the GLUCOSE_METABOLISM pathway
	ATM		-0.1768	Gene of the STEM_CELLS pathway
**DLAT**	SDHD	**0.75**	0.7804	Gene of the GLUCOSE_METABOLISM pathway
	ATM		0.2195	Gene of the STEM_CELLS pathway
	MAZ		0.1710	Candidate regulatory gene of the GLUCOSE_METABOLISM pathway
	ACO2		0.1541	Gene of the GLUCOSE_METABOLISM pathway
	MAML1		0.1416	Gene of the STEM_CELLS pathway
	MERTK		0.1310	Gene of the STEM_CELLS pathway
	ZBTB1		-0.0636	Candidate regulatory gene of the STEM_CELLS pathway
**HK3**	FBP1	**0.71**	0.5655	Gene of the GLUCOSE_METABOLISM pathway
	PTPRC		0.4751	Gene of the STEM_CELLS pathway
	MERTK		0.0898	Gene of the STEM_CELLS pathway
	PLAT		-0.0713	Gene of the STEM_CELLS pathway
	ALDH1A1		-0.0812	Gene of the STEM_CELLS pathway
	MS4A1		-0.0924	Gene of the STEM_CELLS pathway
	ZHX1		-0.1459	Candidate regulatory gene of the GLUCOSE_METABOLISM pathway
**ACO2**	L3MBTL2	**0.69**	0.5854	Candidate regulatory gene of the model gene ACO2
	ESRRA		0.2703	Candidate regulatory gene of the model gene ACO2
	IDH3A		0.2294	Gene of the GLUCOSE_METABOLISM pathway
	ZHX2		0.1470	Candidate regulatory gene of the GLUCOSE_METABOLISM pathway
	HNRNPUL1		0.1261	Candidate regulatory gene of the STEM_CELLS pathway
	ILK		0.1245	Candidate regulatory gene of the GLUCOSE_METABOLISM pathway
	SUCLG1		0.1078	Gene of the GLUCOSE_METABOLISM pathway
	NOTCH2		0.0843	Gene of the STEM_CELLS pathway
	CHEK1		-0.1221	Gene of the STEM_CELLS pathway
	LATS1		-0.2098	Gene of the STEM_CELLS pathway
**ACLY**	STAT3	**0.67**	0.2612	Gene of the STEM_CELLS pathway
	ZNF207		0.2587	Candidate regulatory gene of the STEM_CELLS pathway
	PARP1		0.2156	Gene of the DNA_REPAIR pathway
	SMARCE1		0.2111	Candidate regulatory gene of the GLUCOSE_METABOLISM pathway
	MEF2D		0.1723	Candidate regulatory gene of the GLUCOSE_METABOLISM pathway
	ITGB1		0.1627	Gene of the STEM_CELLS pathway
	G6PC3		0.1478	Gene of the GLUCOSE_METABOLISM pathway
	NFXL1		0.1440	Candidate regulatory gene of the GLUCOSE_METABOLISM pathway
	SNAI1		0.0896	Gene of the STEM_CELLS pathway
	ATF1		-0.1019	Candidate regulatory gene of the model gene ACLY
	ZNF354B		-0.1208	Candidate regulatory gene of the GLUCOSE_METABOLISM pathway
	REST		-0.1437	Candidate regulatory gene of the GLUCOSE_METABOLISM pathway
**PRPS1**	PDK3	**0.63**	0.2945	Gene of the GLUCOSE_METABOLISM pathway
	NONO		0.2459	Candidate regulatory gene of the GLUCOSE_METABOLISM pathway
	PHB2		0.2453	Candidate regulatory gene of the GLUCOSE_METABOLISM pathway
	PGK1		0.2334	Gene of the GLUCOSE_METABOLISM pathway
	CHAMP1		0.1906	Candidate regulatory gene of the GLUCOSE_METABOLISM pathway
	NKRF		0.1762	Candidate regulatory gene of the GLUCOSE_METABOLISM pathway
	PECAM1		0.1548	Gene of the STEM_CELLS pathway
	PCK2		0.1337	Gene of the GLUCOSE_METABOLISM pathway
	ZZZ3		0.1298	Candidate regulatory gene of the GLUCOSE_METABOLISM pathway
	METHYLATION (PRPS1		0.0805	Methylation of the model gene PRPS1
**PHKA1**	TAF1	**0.63**	0.3758	Candidate regulatory gene of the GLUCOSE_METABOLISM pathway
	SMO		0.3157	Gene of the STEM_CELLS pathway
	HDAC8		0.2654	Candidate regulatory gene of the GLUCOSE_METABOLISM pathway
	ELK1		0.1919	Candidate regulatory gene of the GLUCOSE_METABOLISM pathway
	MYC		0.1805	Candidate regulatory gene of the model gene PHKA1
	PRPS2		0.1168	Gene of the GLUCOSE_METABOLISM pathway
	KIT		0.1022	Gene of the STEM_CELLS pathway
	ETFA		0.0998	Gene of the STEM_CELLS pathway
	BRCA1		-0.0774	Gene of the DNA_REPAIR pathway
	TCF7L2		-0.0972	Candidate regulatory gene of the GLUCOSE_METABOLISM pathway
	CD44		-0.1002	Gene of the STEM_CELLS pathway
	ENG		-0.1125	Gene of the STEM_CELLS pathway
	KLF17		-0.1212	Gene of the STEM_CELLS pathway
	GSK3B		-0.1431	Gene of the GLUCOSE_METABOLISM pathway
	MXI1		-0.1740	Candidate regulatory gene of the GLUCOSE_METABOLISM pathway
**ALDOA**	PHKG2	**0.62**	0.3420	Gene of the GLUCOSE_METABOLISM pathway
	MAZ		0.2566	Candidate regulatory gene of the model gene ALDOA
	MDH2		0.2218	Gene of the GLUCOSE_METABOLISM pathway
	CD44		0.2031	Gene of the STEM_CELLS pathway
	ITGA4		0.1688	Gene of the STEM_CELLS pathway
	G6PD		0.1163	Gene of the GLUCOSE_METABOLISM pathway
	ACO2		0.0992	Gene of the GLUCOSE_METABOLISM pathway
	STAT3		0.0908	Gene of the STEM_CELLS pathway
	FANCF		-0.0831	Gene of the DNA_REPAIR pathway
	MERTK		-0.1016	Gene of the STEM_CELLS pathway
	ITGA6		-0.1060	Gene of the STEM_CELLS pathway
	ENO3		-0.1297	Gene of the GLUCOSE_METABOLISM pathway
	ADNP		-0.1406	Candidate regulatory gene of the GLUCOSE_METABOLISM pathway
	TAF15		-0.1769	Candidate regulatory gene of the GLUCOSE_METABOLISM pathway
	PTPRC		-0.2804	Gene of the STEM_CELLS pathway
	SMO		-0.3179	Gene of the STEM_CELLS pathway

Figure S1.6: Model M5 best GLUCOSE_METABOLISM genes and their regulatory features.

S2. Computational evaluation: ARACNe

ARACNe is a an algorithm for the reconstruction of gene regulatory networks in a mammalian cellular context: starting from gene expression data, it generates gene expression networks by considering triplets of genes and iteratively removing the weakest of the three relationships at each iteration, according to its assigned Mutual Information (MI) value and to an arbitrary threshold (I_0) set *a priori* by the user (i.e., only relationships between genes quantified by a MI value higher than I_0 are considered and, for each triplet, the edge with the lowest MI value is removed). The threshold allows defining the dimension of the generated network: the higher is I_0, the smaller are the networks and the number of displayed relationships. An example of how the dimension of the output network changes in relation to different values of the threshold I_0 is shown in Figure S2.1: nodes represent the genes of interest, while edges identify their correlations computed by ARACNe.

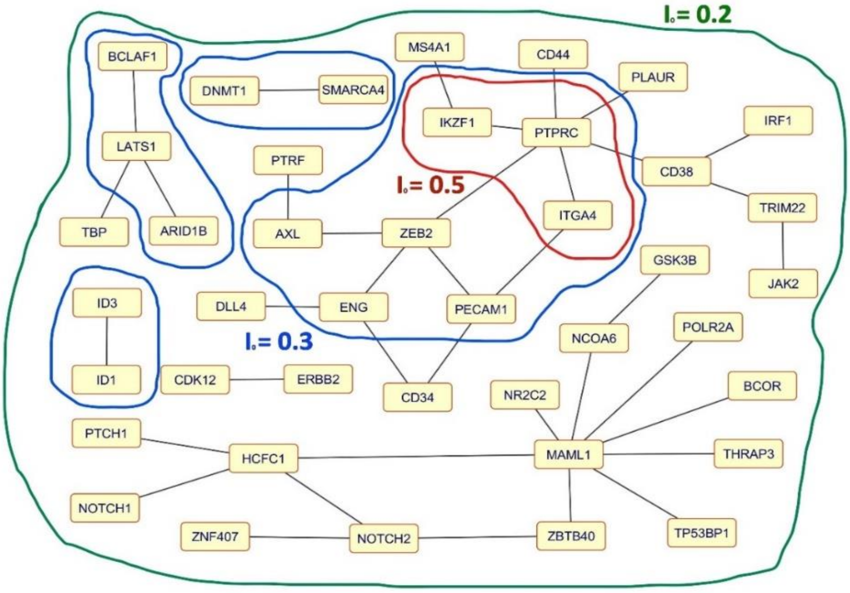

Figure S2.1: Example of the effect of different mutual information thresholds (I_0) in ARACNe.

ARACNe works under a specific set of initial approximations that, although proved to be reasonable, may lead the algorithm to wrong network inferences. Being a computational method, there is no guarantee that its results are correct or complete; some relationships it identifies may be wrong, as well as it may miss some relationships. However, being a recognized computational method, we used ARACNe for evaluating the associations found during our data analysis, by comparing our ovarian cancer regression model networks with the corresponding networks generated by the ARACNe algorithm. Depending on how this comparison is made and due to the fact that the linear regression algorithm is a different computational procedure with respect to the ARACNe algorithm, differences are expected. Specifically, according to the computational approach we defined, which is focused on identifying best-predicting sets of regulators although in case omitting potential regulators with a lower predicting power, the regression models computed on a subset of selected features can detect less correlations than ARACNe, although some of such correlations may not be found by ARACNe. In addition, ARACNe is made for building only gene expression networks without taking methylation or other regulatory elements into account, as conversely our novel modeling allows.

In the end, as a result of this comparison, for most target genes a set of common features between the two approaches is expected, i.e., a set of the main relationships revealed by the regression models that are also extracted by the ARACNe algorithm: this allows verifying that our initial aim and its implementation through a combined feature selection and linear regression approach leads to meaningful results that are worth investigating through further biological experimental analyses.

We processed through the ARACNe algorithm the information contained in M3 and M5 matrices for the Ovarian Serous Cystadenocarcinoma, generating two corresponding networks. As done for our linear regression procedure, this processing was performed for each considered set of genes, i.e., for the sets of genes belonging to the DNA_REPAIR, STEM_CELLS, or GLUCOSE_METABOLISM pathway (see *Section S0.1*). For each pathway, the two networks corresponding to the M3 and M5 matrices, respectively, that were generated using ARACNe were then used for the comparison: the former network takes the genes of the pathway and the complete set of their candidate regulators as input, along with their expression values in the different ovarian cancer TCGA samples, to allow a complete processing using the list of genes in the pathway as hubs of the network; the latter network adds expression information about genes of the other considered pathways and their candidate regulators, to allow a complete processing by setting the list of genes in the studied pathway as nodes and the list of their candidate regulatory genes as transcription factors.
The whole process is executed by setting the lowest possible Mutual Information threshold (I_0 = 0.001), in order to obtain the highest number of correlations. The generated networks (six in total) are then compared with the corresponding ones from our regression models based on the M3 and M5 matrices for the same pathways. Figure S2.2 summarizes the set of operations performed for the generation of pathway gene networks by our regression models and by ARACNe for their comparison.

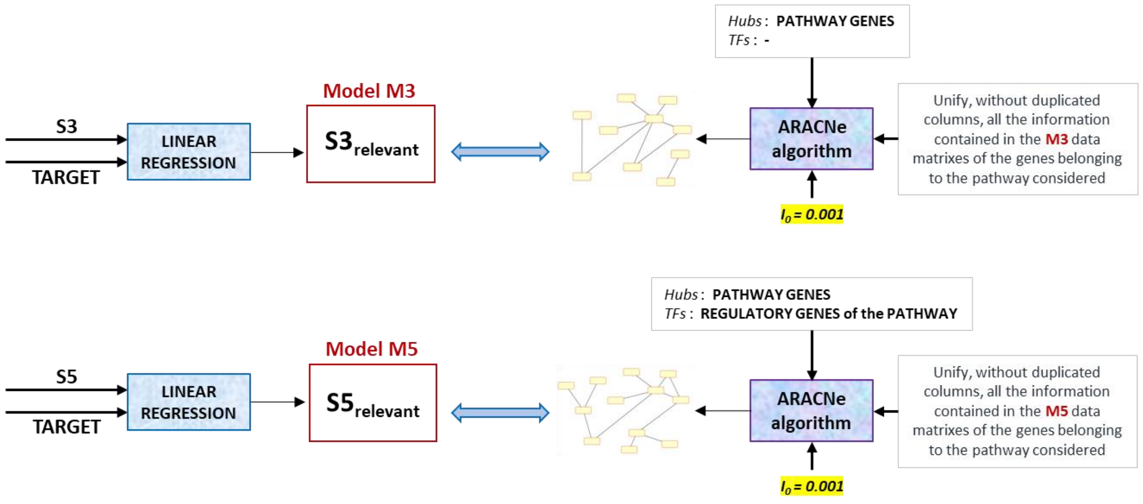

Figure S2.2 Processing for the generation of pathway gene networks by regression models vs. by ARACNe.

Overall comparison of results for the considered ovarian cancer data are provided in Figure S2.3, while Figure S2.4 shows an example of the detailed comparison for some genes in M5. The latter one was performed by matching relevant features found associated with the indicated gene through our regression models or through ARACNe, and by assessing their ranking among the gene associated features found, based on the association regression coefficient or mutual information, respectively; such rankings resulted very similar overall.

		M3		M5	
	Total N° Genes in the pathway	% of genes with common features in OV Models and ARACNe	Mean % OV Models features found by ARACNe	% of genes with common features in OV Models and ARACNe	Mean % OV Models features found by ARACNe
DNA_REPAIR	20	**90.0%**	37.4%	**95.0%**	35.4%
GLUCOSE_METABOLISM	84	**83.3%**	27.4%	**84.5%**	27.8%
STEM_CELLS	73	**72.6%**	26.5%	**73.9%**	26.4%

Figure S2.3: Overall comparison of ovarian cancer (OV) results by regression models vs. by ARACNe.

N° ARACNe Features	N° OV Models Features	Common Features	Mutual Information	Regression Coefficient	ARACNe Ranking	OV Models Ranking	% OV Models features found by ARACNE
**Gene FANCA**							
19	6	POLE	0.2337	0.3805	1	1	66.7%
		E2F4	0.1112	0.2595	3	2	
		MYBL2	0.1796	0.218	2	3	
		ZBTB1	0.0425	0.0841	4	4	
**Gene TP53BP1**							
94	3	ZSCAN29	0.5494	0.4646	1	1	100%
		ZBTB40	0.3848	0.3737	2	2	
		MAML1	0.2499	0.1917	3	3	
**Gene CHEK1**							
46	12	NFRKB	0.1022	0.4343	3	1	50%
		RAD51	0.15	0.2605	1	2	
		FANCA	0.1048	0.2058	2	3	
		CBX5	0.0937	0.129	4	4	
		SRSF1	0.0825	0.129	5	5	
		RB1	0.0132	-0.168	6	6	
**Gene PTPRC**							
13	7	IKZF1	0.7776	0.5857	1	1	57.1%
		ITGA4	0.5026	0.2392	2	2	
		CD38	0.254	0.1394	4	3	
		CD44	0.2987	0.1217	3	4	
**Gene DLAT**							
13	7	SDHD	0.3366	0.7804	1	1	28.6%
		ATM	0.12	0.2195	2	2	

Figure S2.4: Comparison of OV results by regression models vs. ARACNe for a sample set of genes.

Finally, Figure S2.5 summarizes the results of the comparison with ARACNe by varying the MI threshold, for each considered pathway.

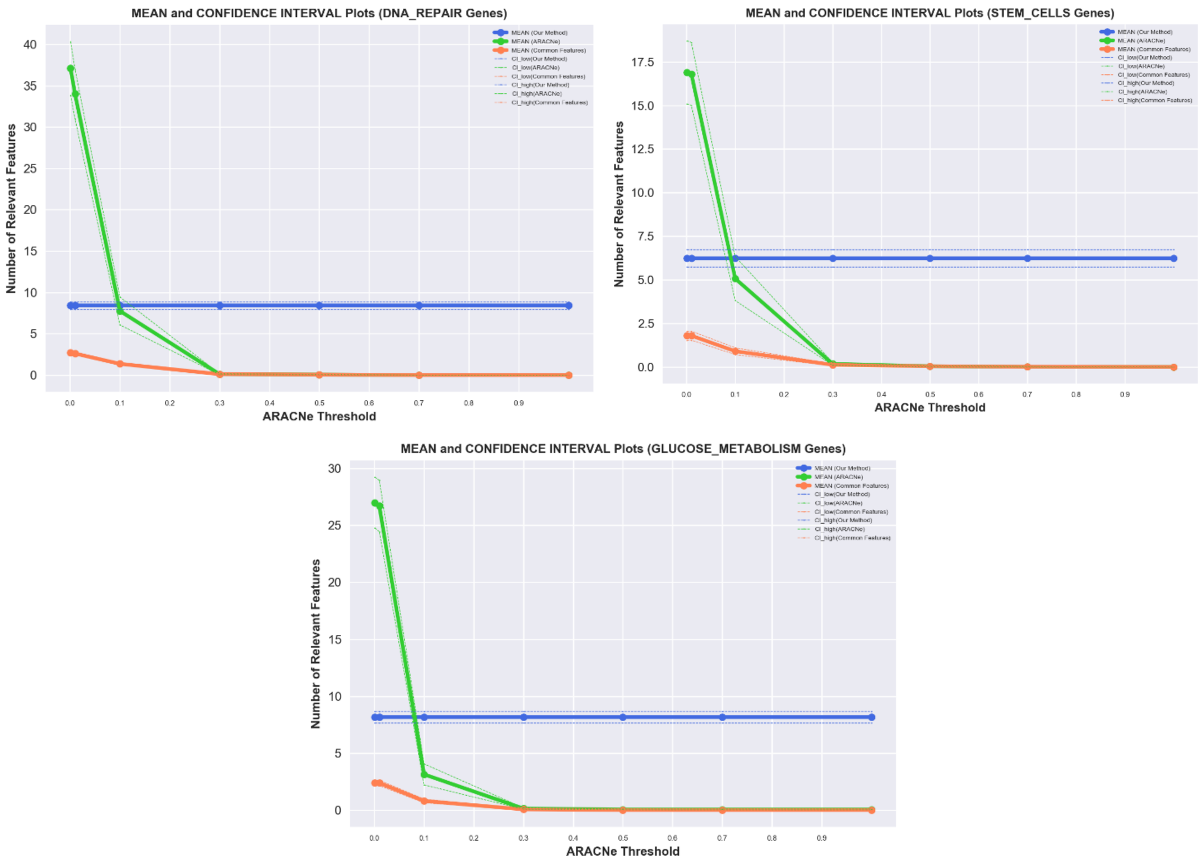

Figure S2.5: Comparison with ARACNe results: mean and confidence intervals of number of relevant features per gene.

S3. Gene expression networks

All our final results are graphically represented through the following reported expression networks, grouping target genes in subclasses according to the considered gene set (i.e., pathway, in our case) they belong to.

All the drawn networks follow the same color code: in red the genes in the DNA_REPAIR pathway, in orange the genes in the STEM_CELLS pathway, in pink the genes in the GLUCOSE_METABOLISM pathway, in green the gene methylations and in light blue the regulatory genes regardless of the pathway they belong to. The relationships are represented by directed edges starting in the regulatory element and incoming in the target gene: red edges correspond to positive regulations, while grey edges correspond to negative regulations, and edge labels correspond to the regression coefficient resulting from the computed model.

S3.1 DNA_REPAIR pathway (M3 models)

<u>Base Excision Repair (BER) pathway subclass</u>

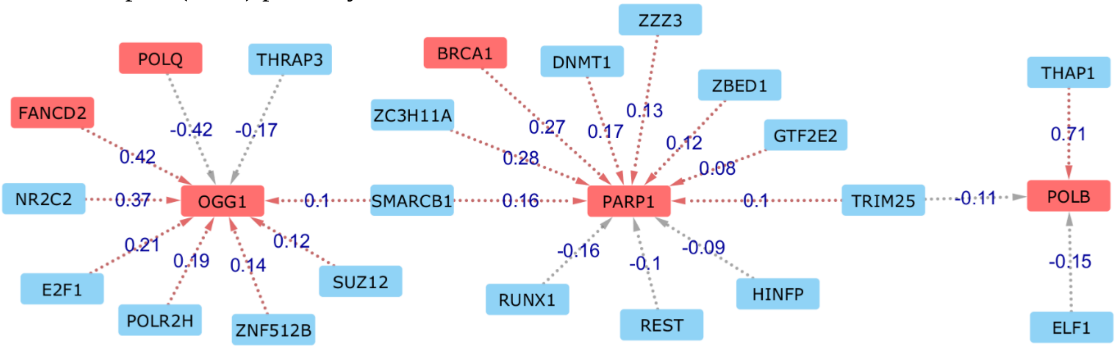

<u>Double Strand Breaks (DSB) pathway subclass</u>

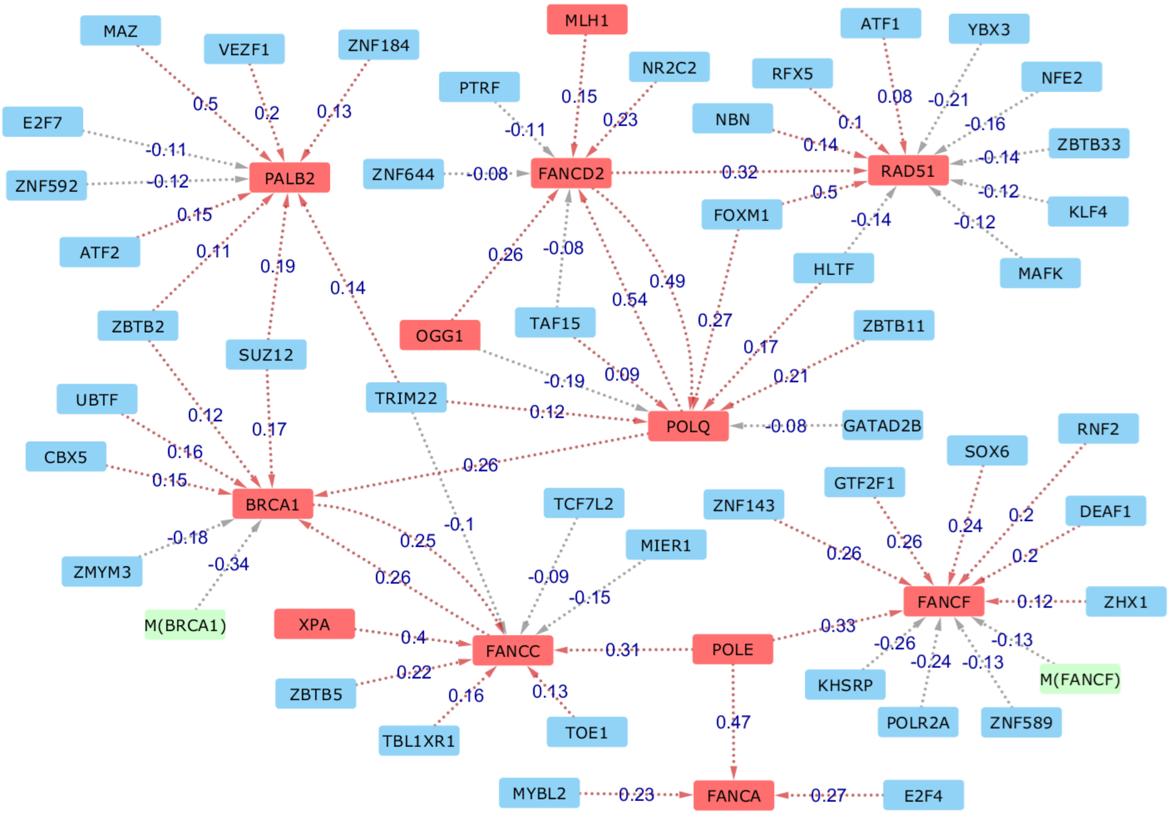

Nucleotide Excision Repair (NER) pathway subclass

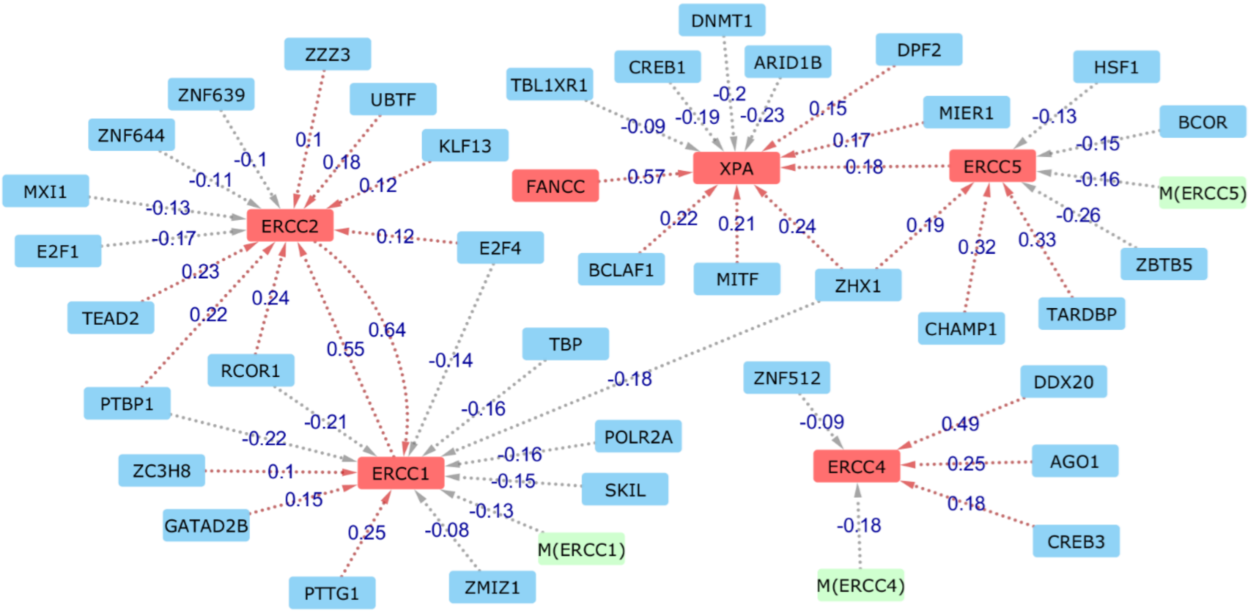

Gene CDK12 Group & Gene MLH1 Group pathway subclass

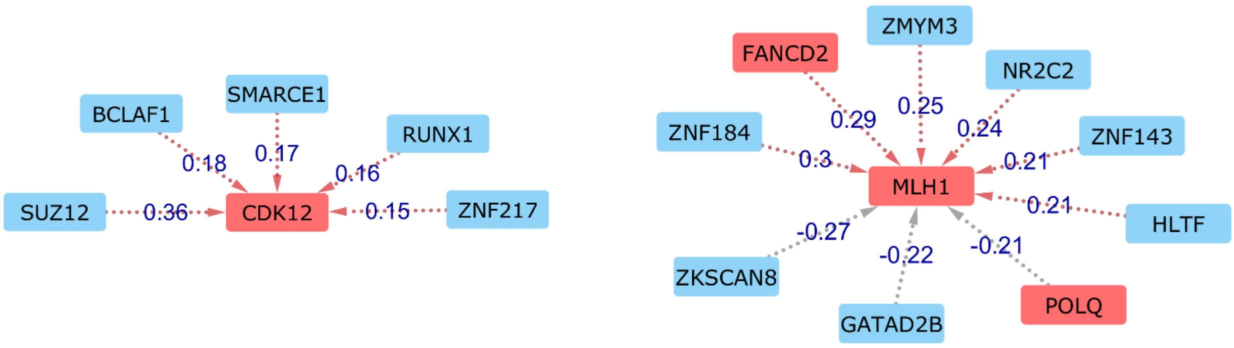

Unclassified pathway subclass

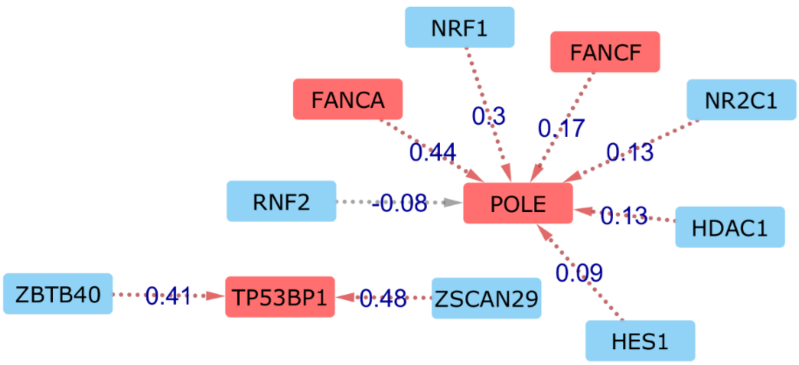

S3.2 DNA_REPAIR pathway (M5 models)

<u>Base Excision Repair (BER) pathway subclass</u>

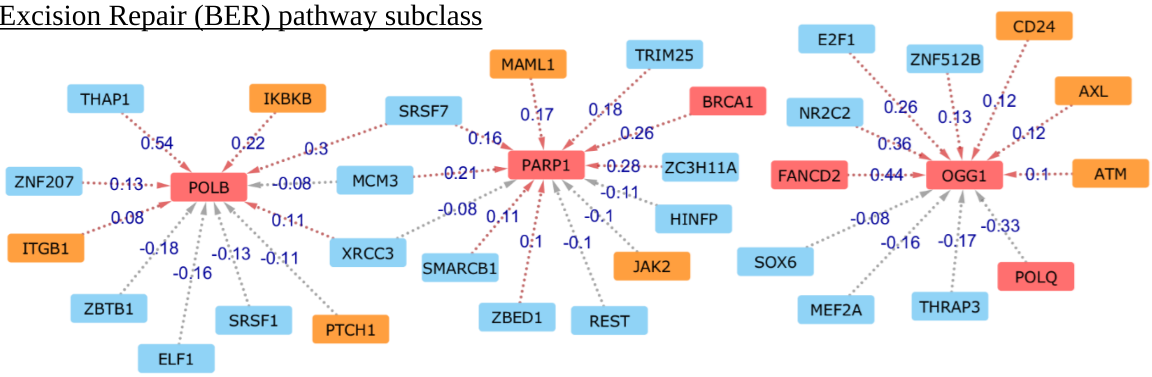

<u>Double Strand Breaks (DSB) pathway subclass</u>

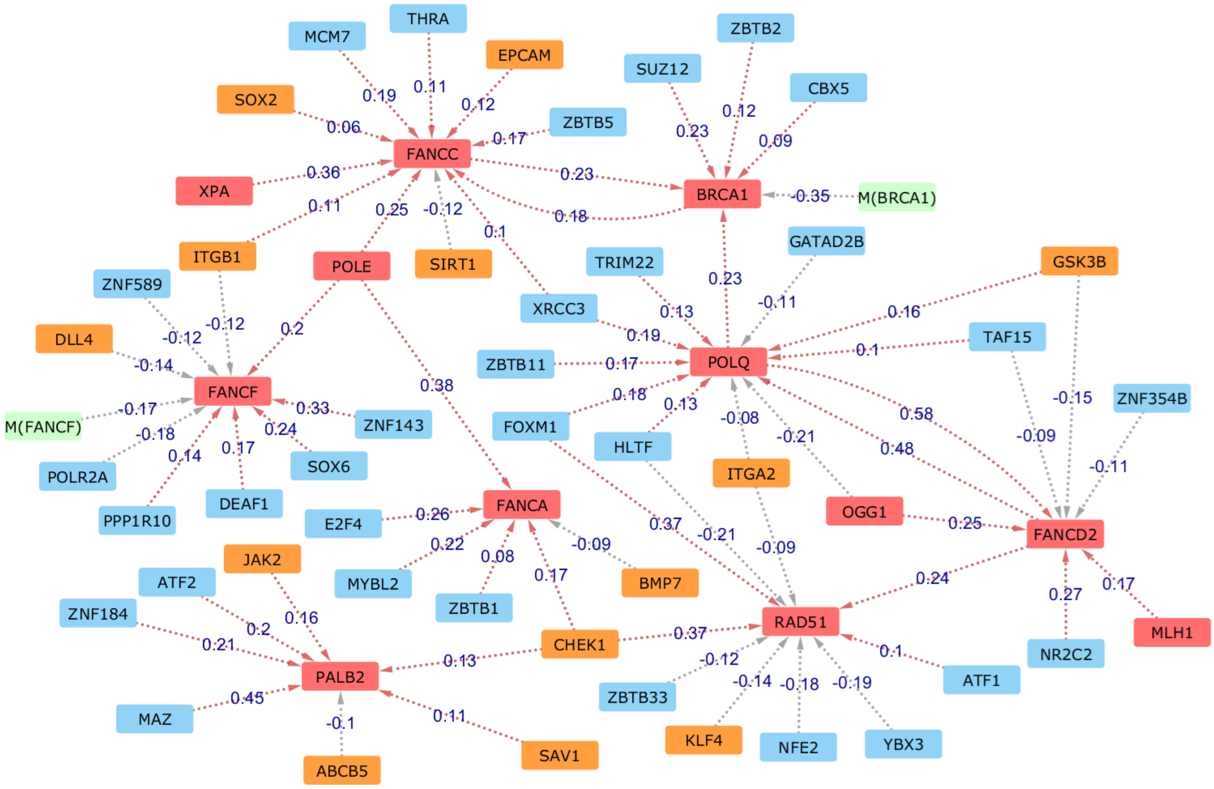

<u>Nucleotide Excision Repair (NER) pathway subclass</u>

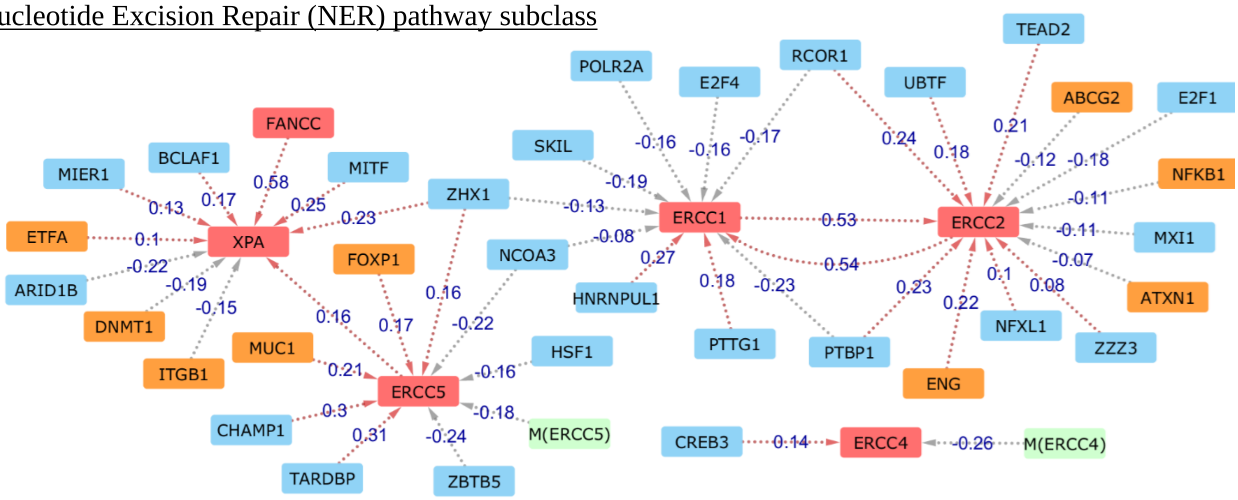

Gene CDK12 Group & Gene MLH1 Group pathway subclass

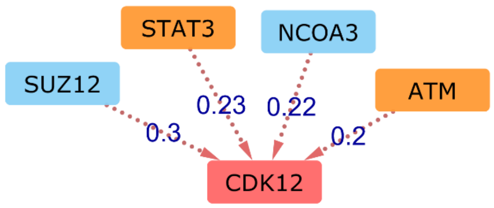

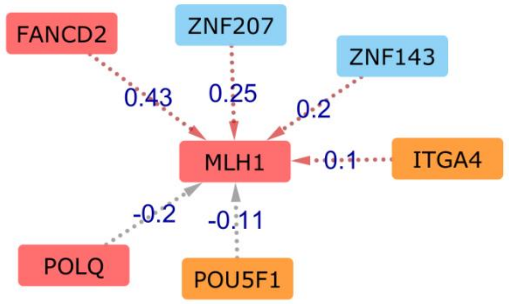

Unclassified pathway subclass

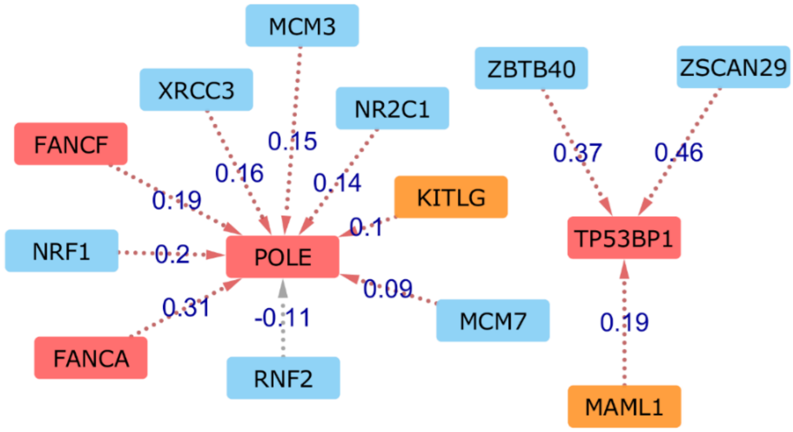

S3.3 STEM_CELLS pathway (M3 models)

AKT & PI3 Kinase – mTOR Signaling pathway subclass

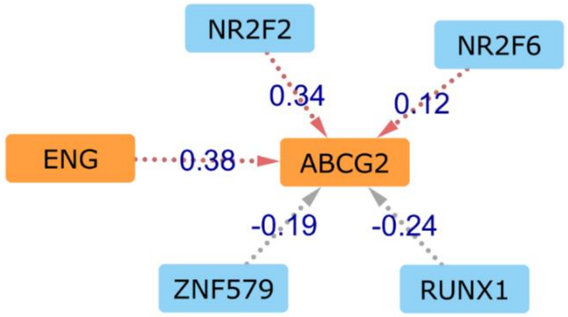

Asymmetric Division pathway subclass

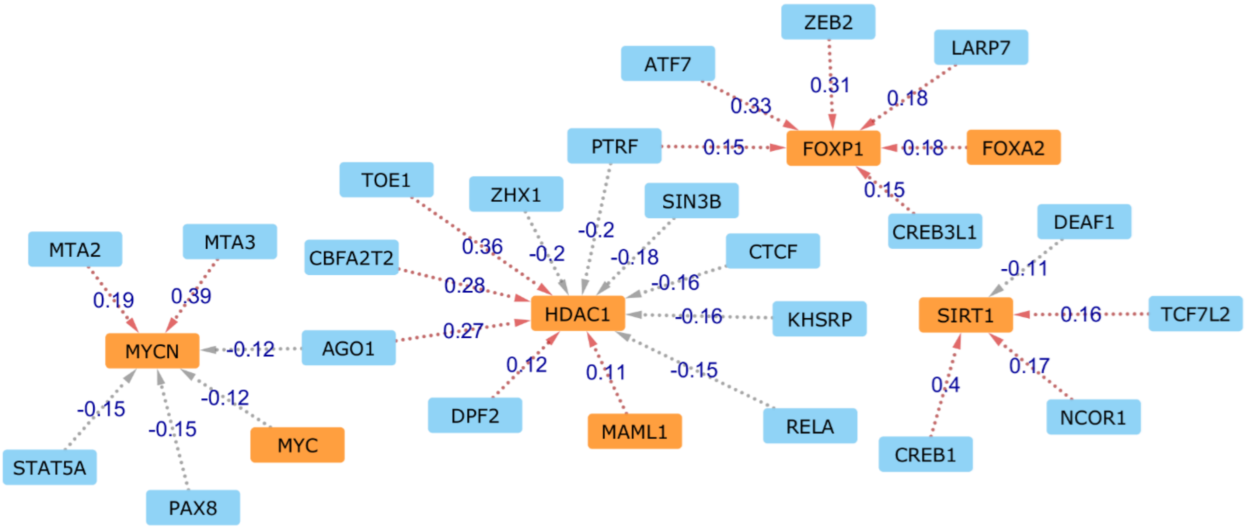

Cancer Stem Cells Markers pathway subclass

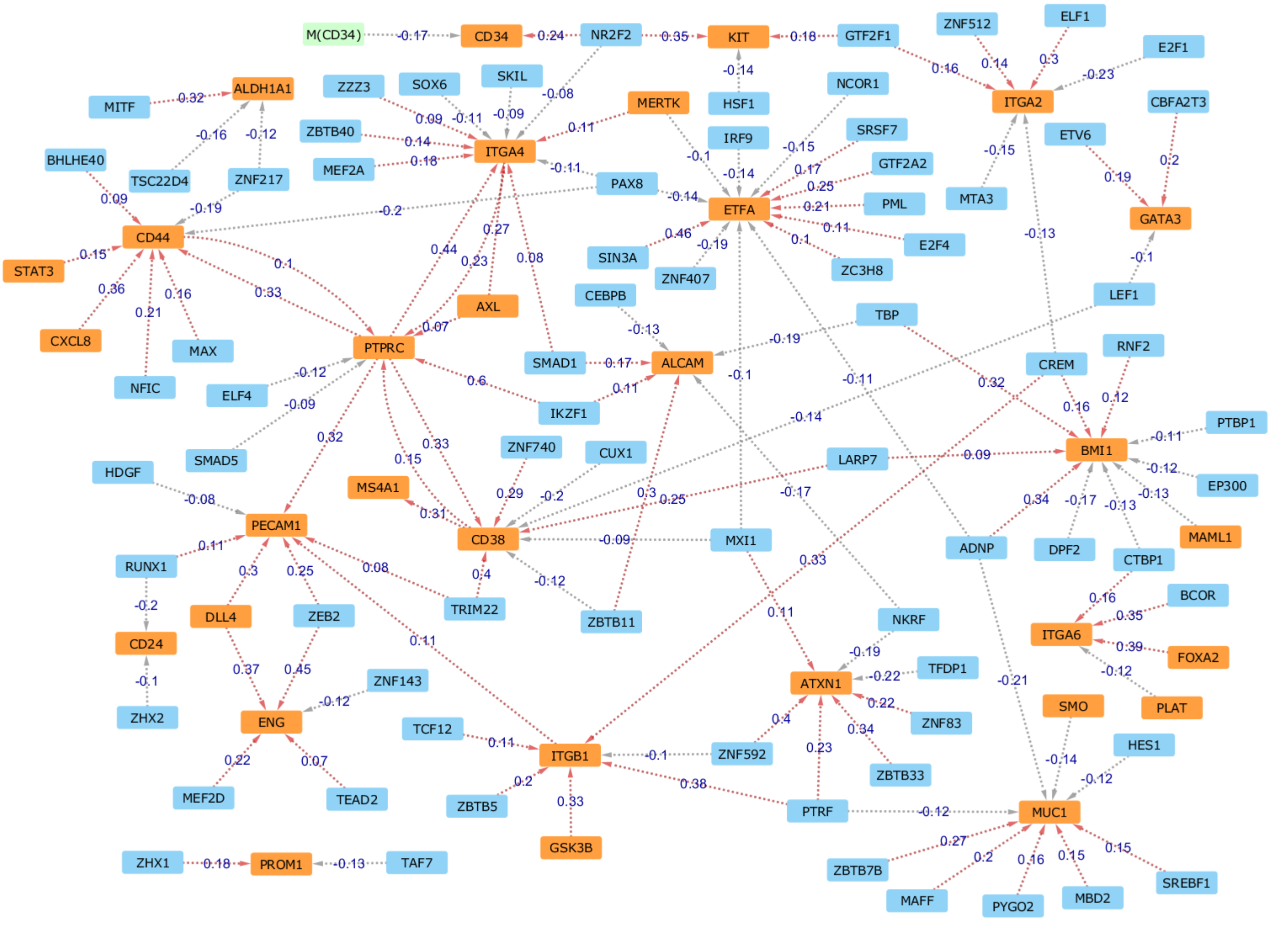

Cancer Therapeutic Targets pathway subclass

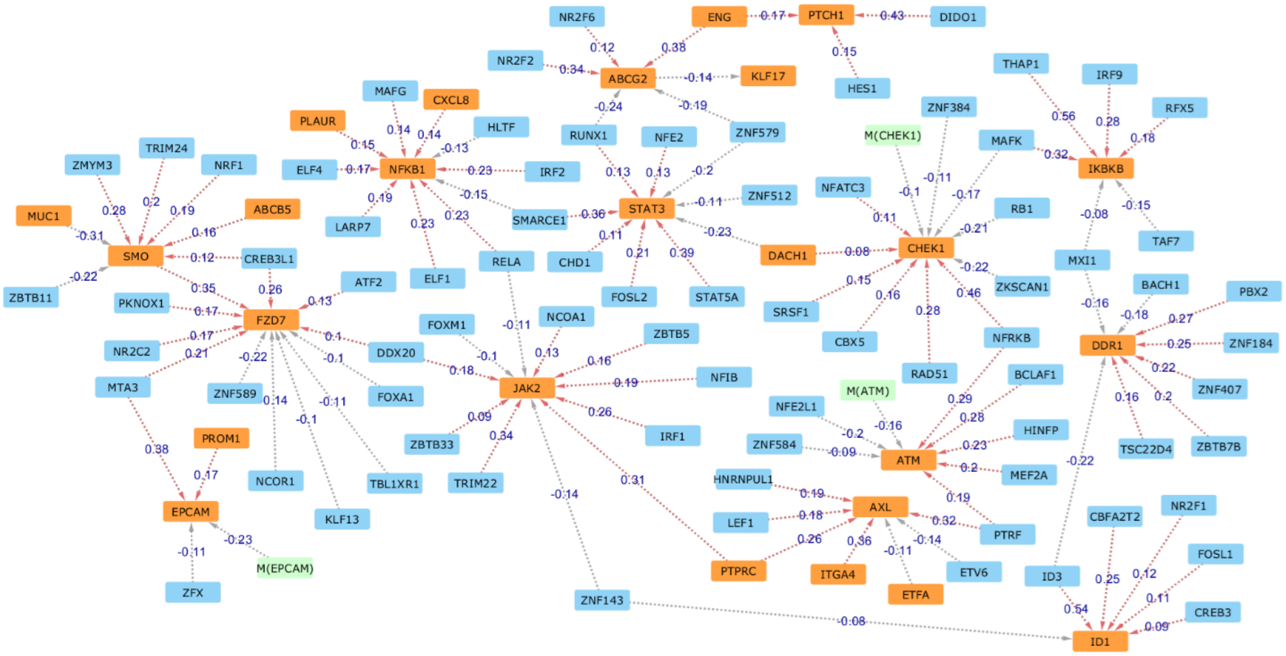

Cell Migration & Metastasis pathway subclass

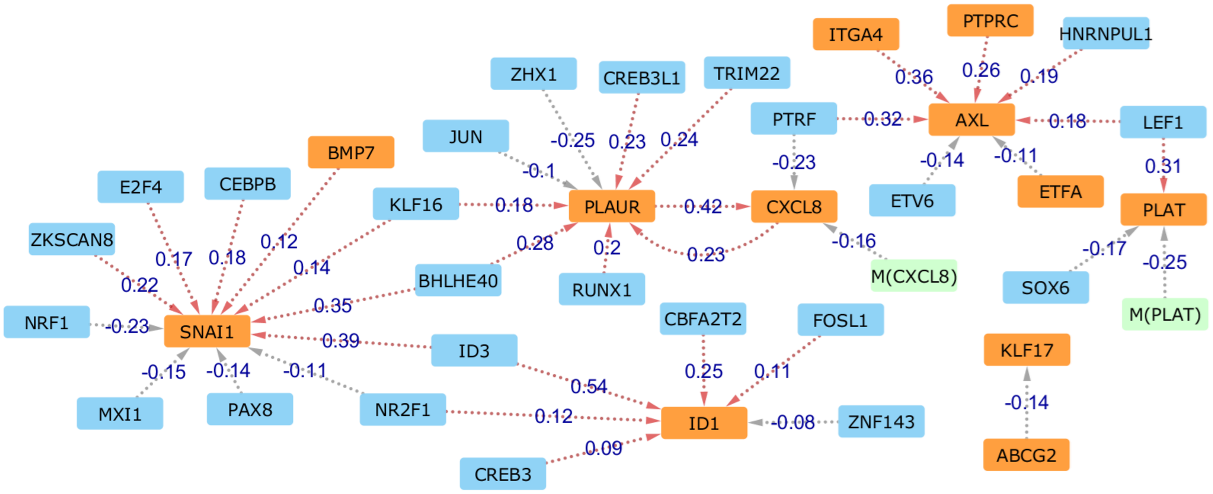

Cell Proliferation pathway subclass

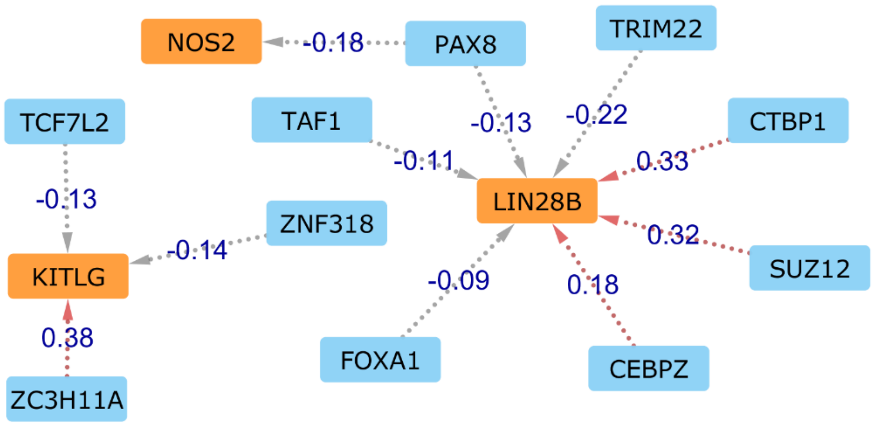

Hedgehog Signaling & Hippo Signaling pathway subclass

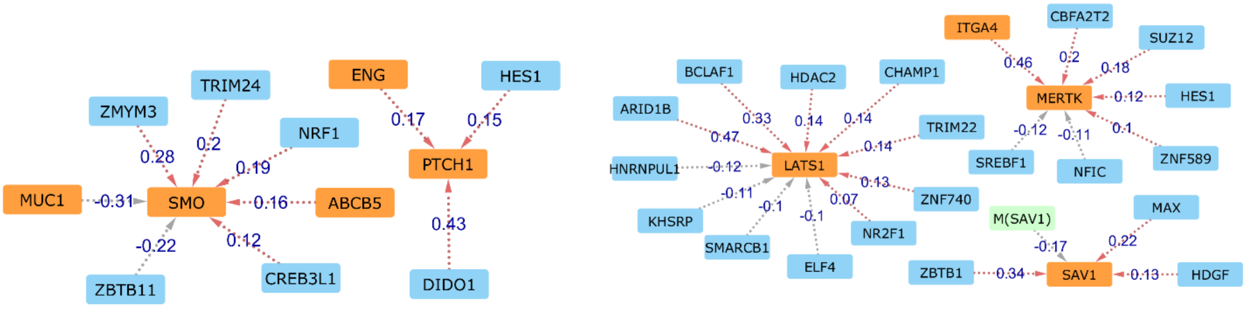

Loss of Stemness pathway subclass

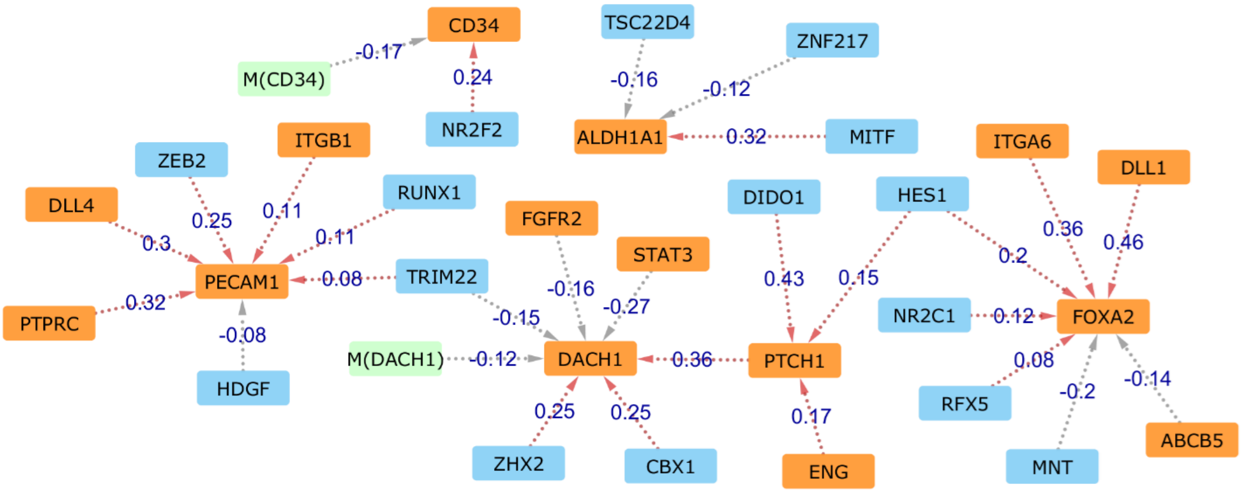

Notch Signaling pathway subclass

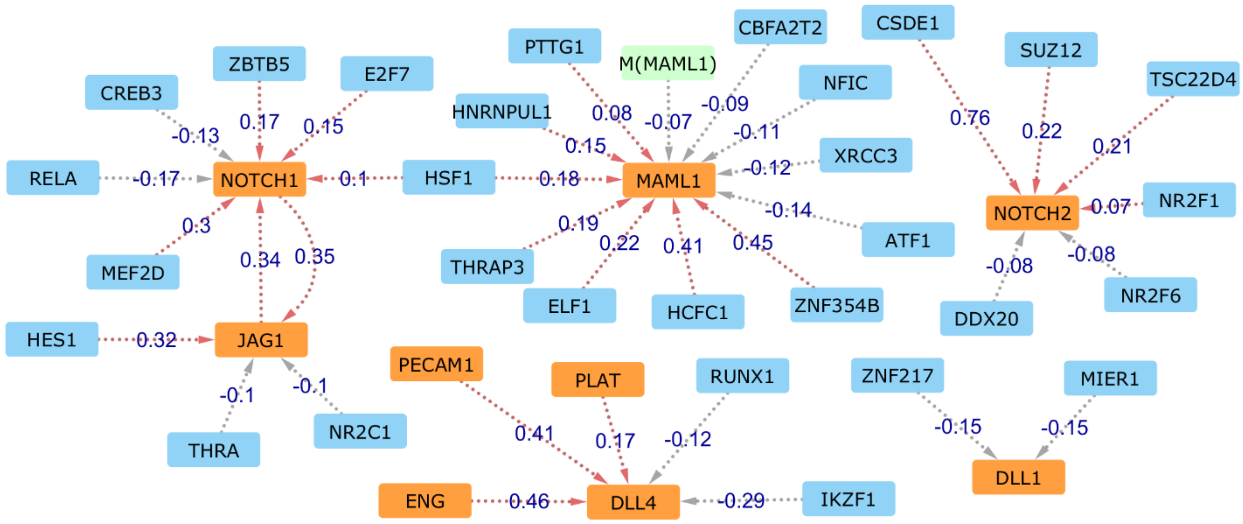

Pluripotency pathway subclass

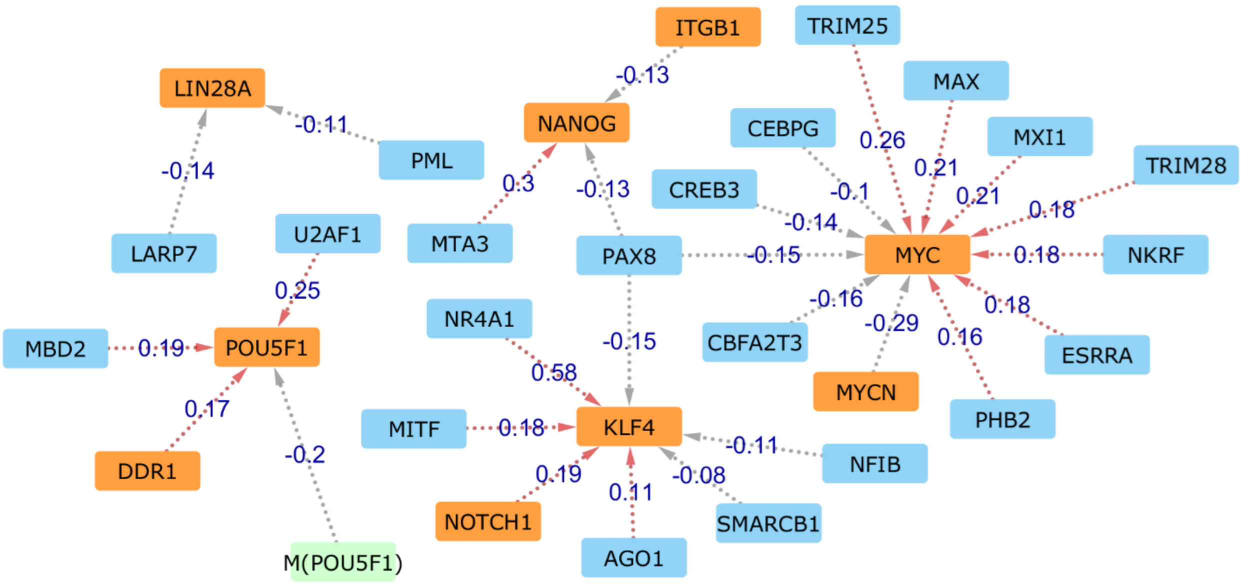

Self-Renewal pathway subclass

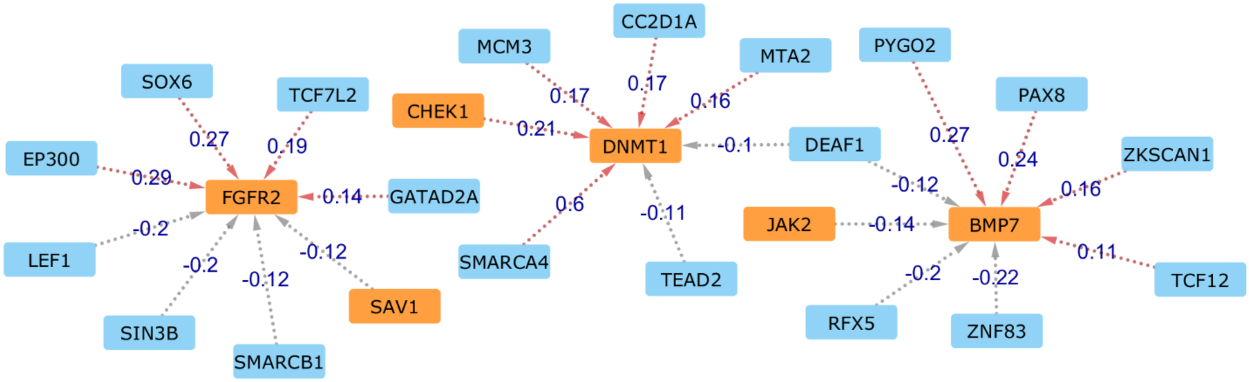

STAT-NFkB Signaling pathway subclass

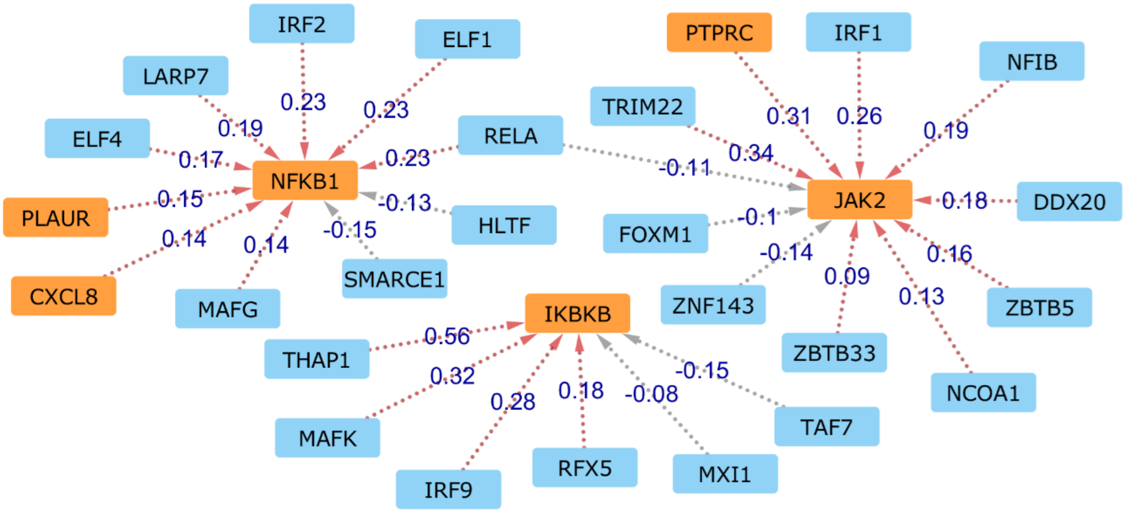

<u>WNT Signaling pathway subclass</u>

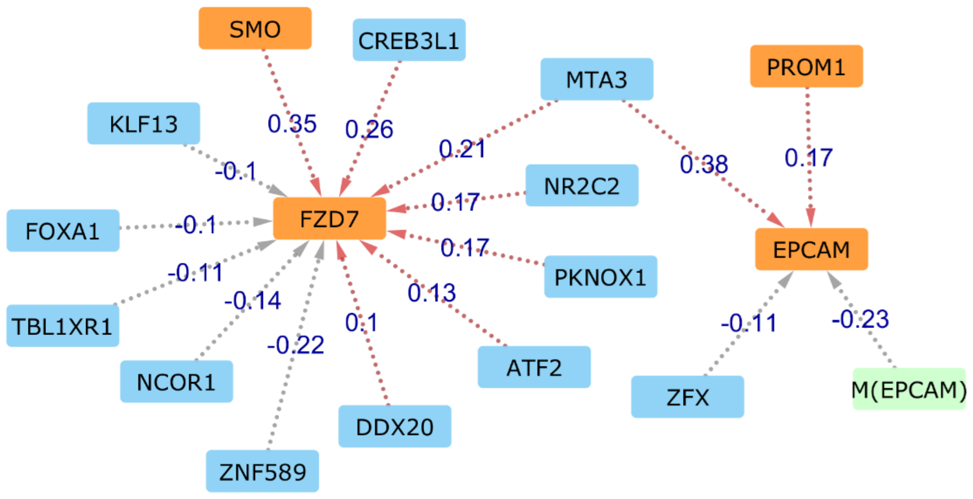

S3.4 STEM_CELLS pathway (M5 models)

<u>AKT & PI3 Kinase – mTOR Signaling pathway subclass</u>

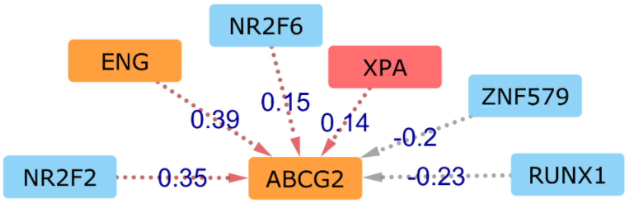

<u>Asymmetric Division pathway subclass</u>

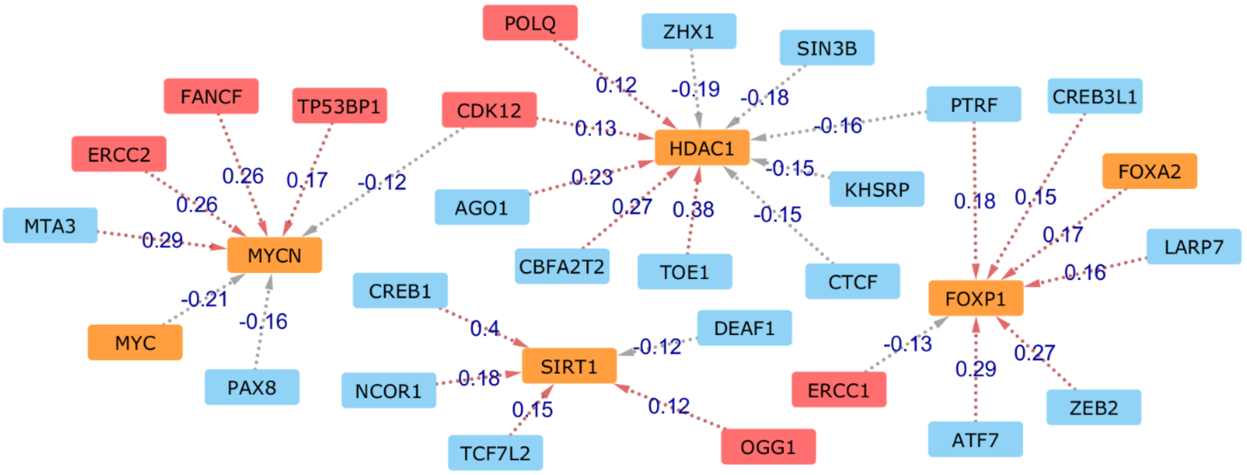

Cancer Stem Cells Markers pathway subclass

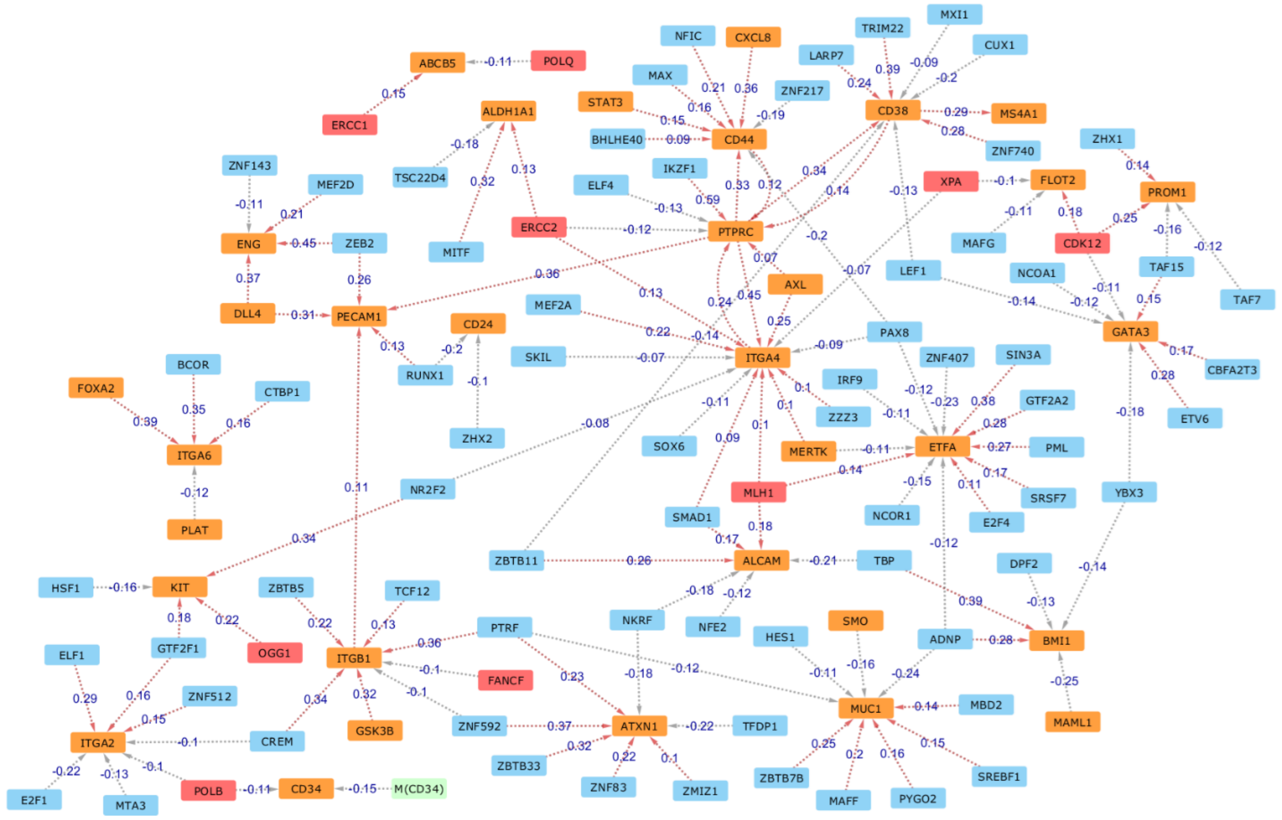

Cancer Therapeutic Targets pathway subclass

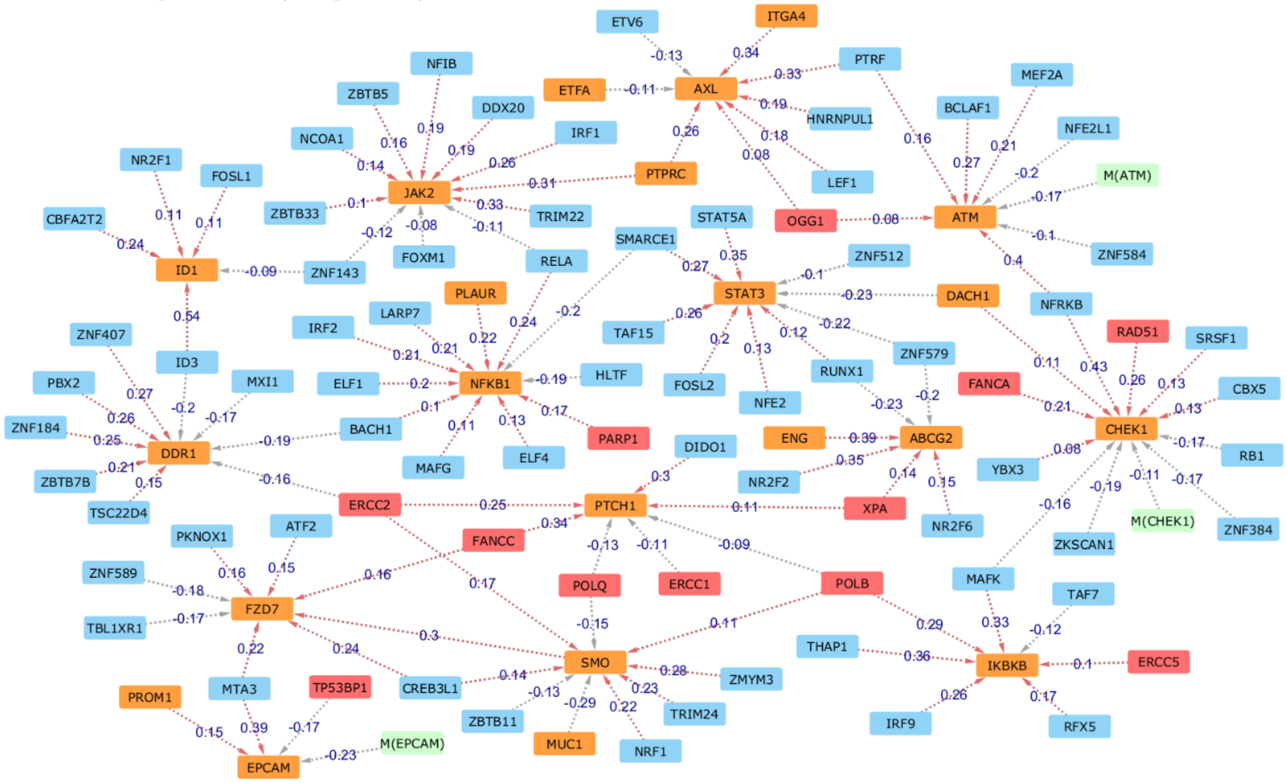

Cell Migration & Metastasis pathway subclass

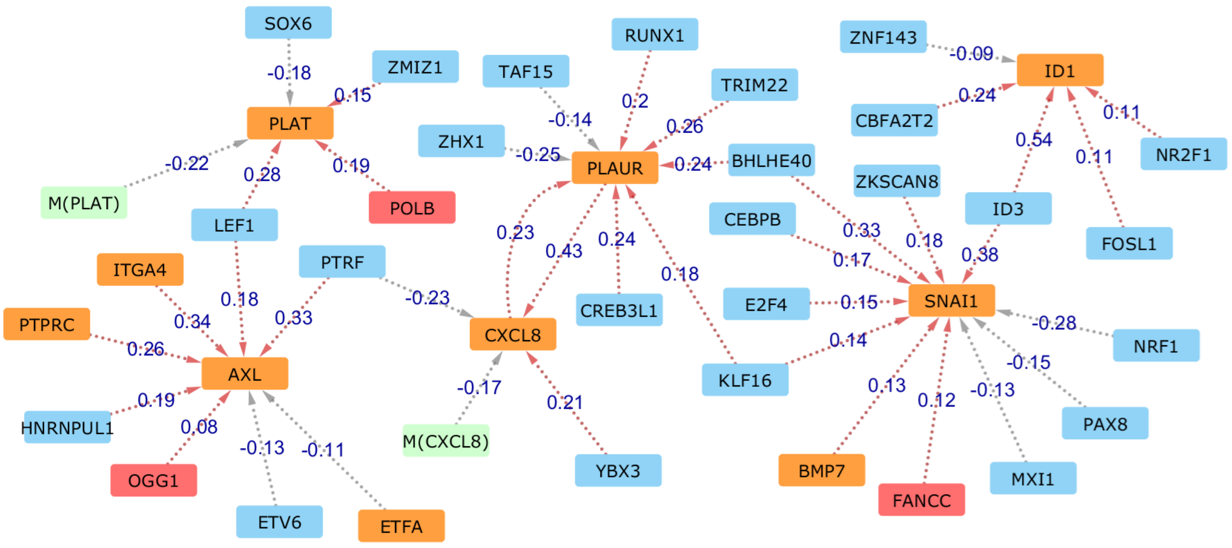

Cell Proliferation pathway subclass

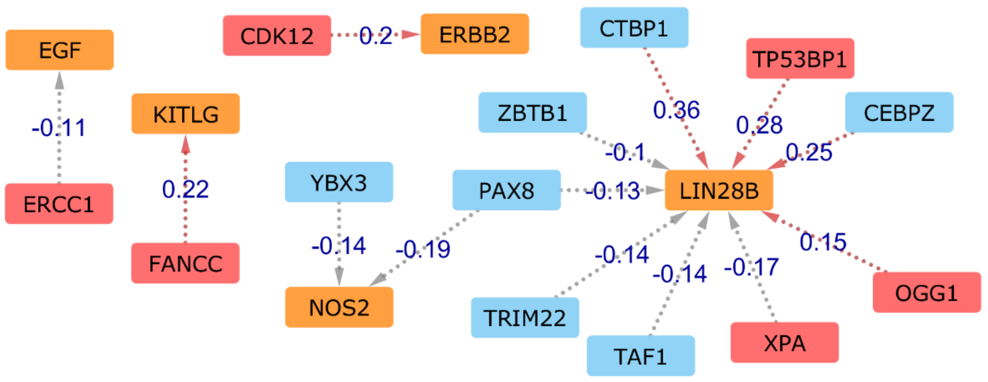

Hedgehog Signaling pathway subclass

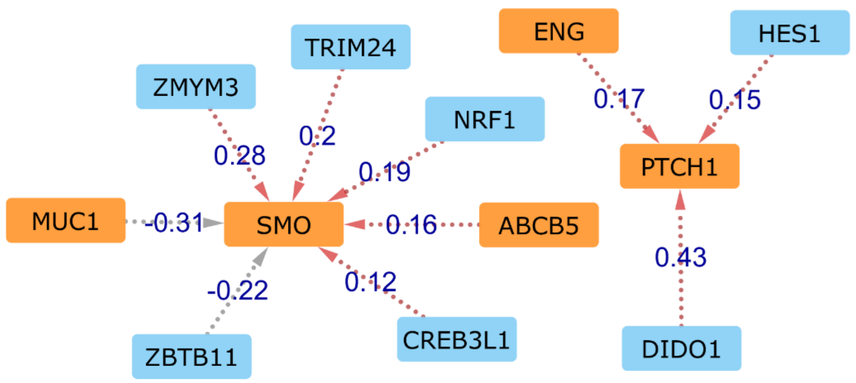

Hippo Signaling pathway subclass

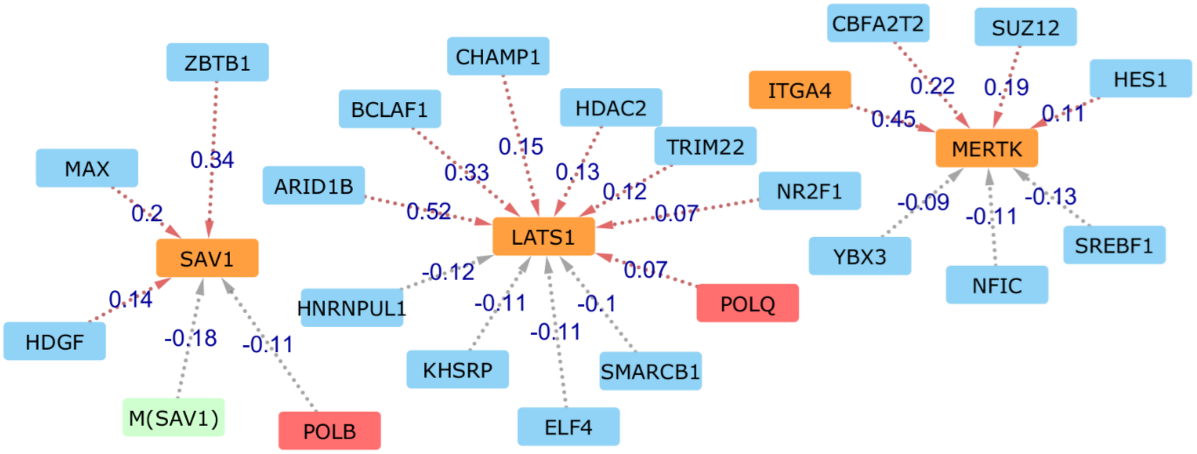

Loss of Stemness pathway subclass

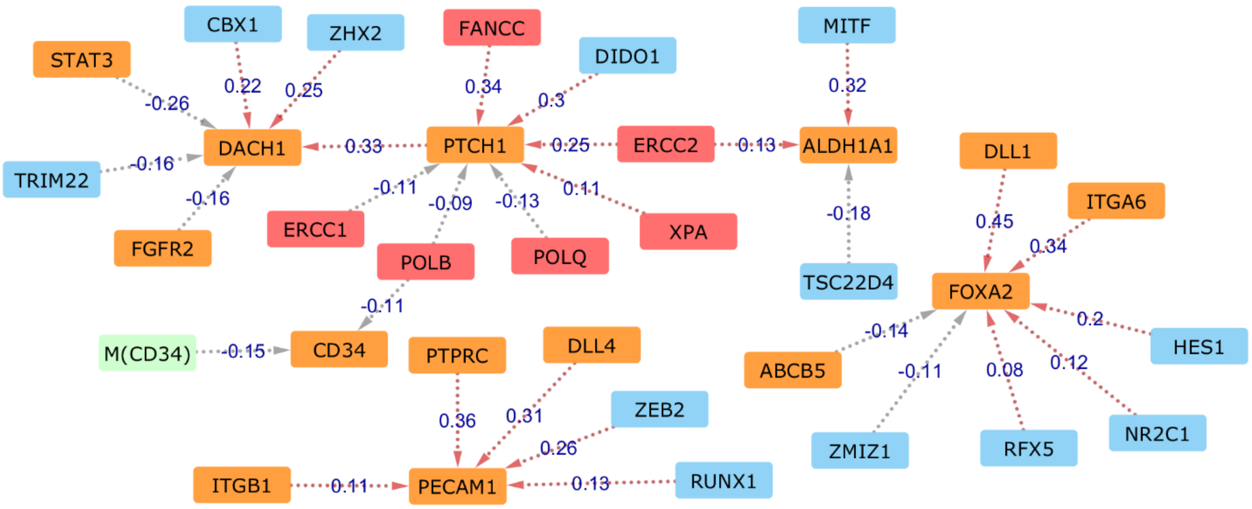

Notch Signaling pathway subclass

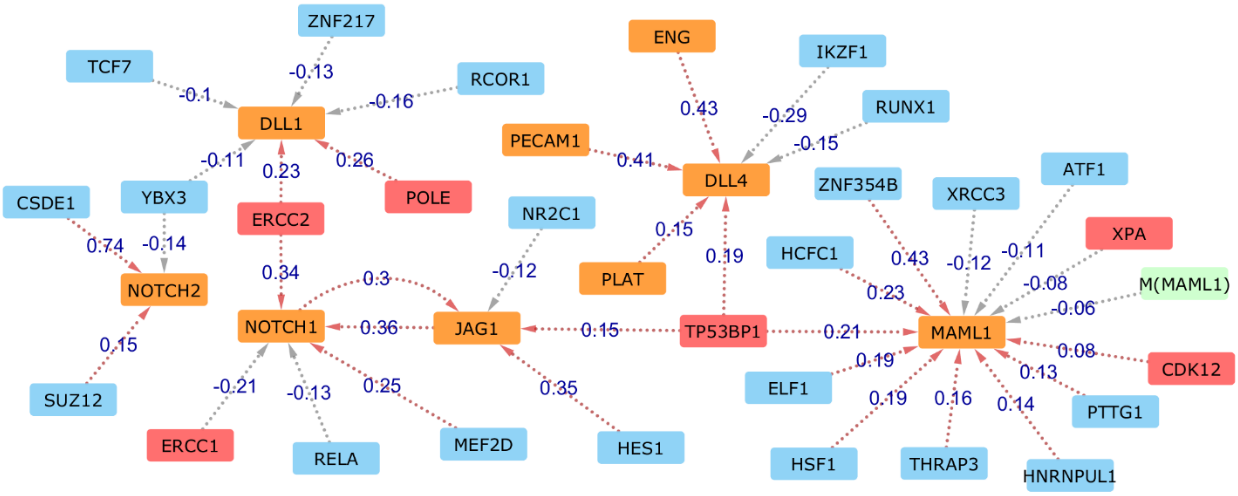

Pluripotency pathway subclass

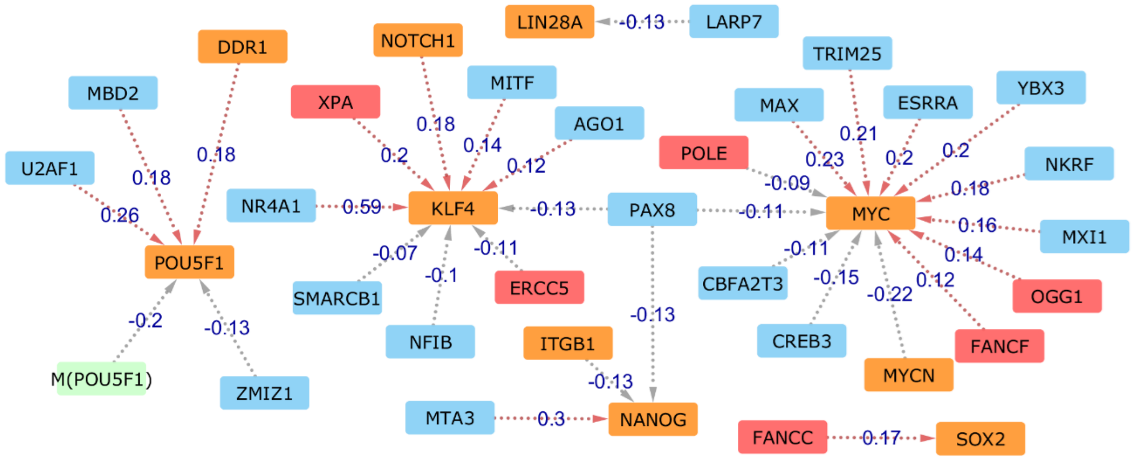

Self-Renewal pathway subclass

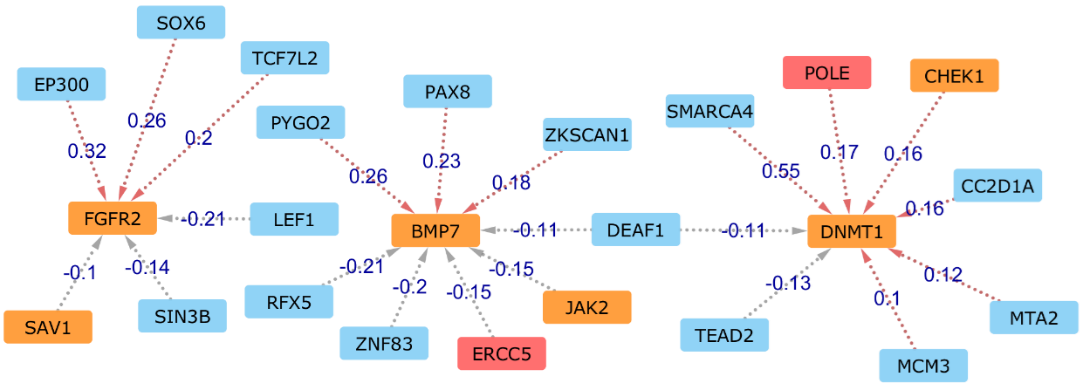

STAT-NFkB Signaling pathway subclass

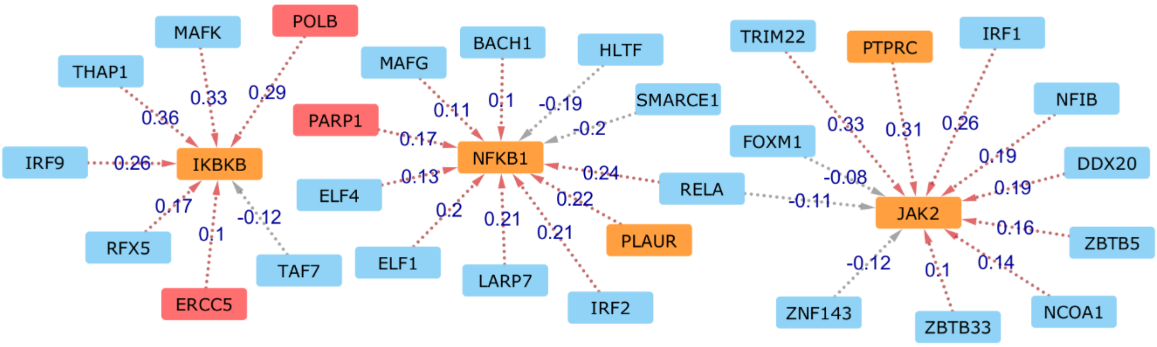

WNT Signaling pathway subclass

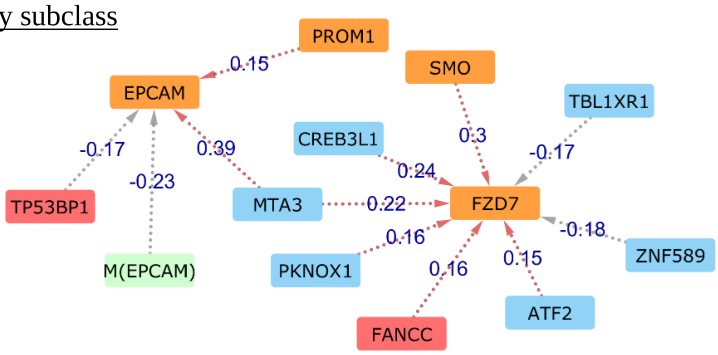

S3.5 GLUCOSE_METABOLISM pathway (M3 models)

Gluconeogenesis pathway subclass

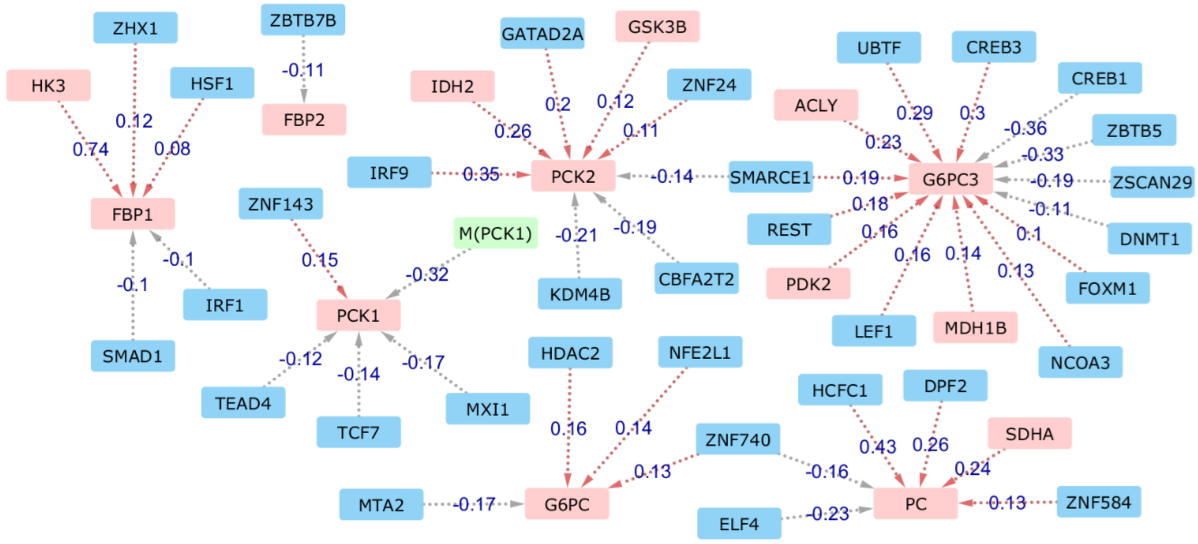

Glycogen Degradation pathway subclass

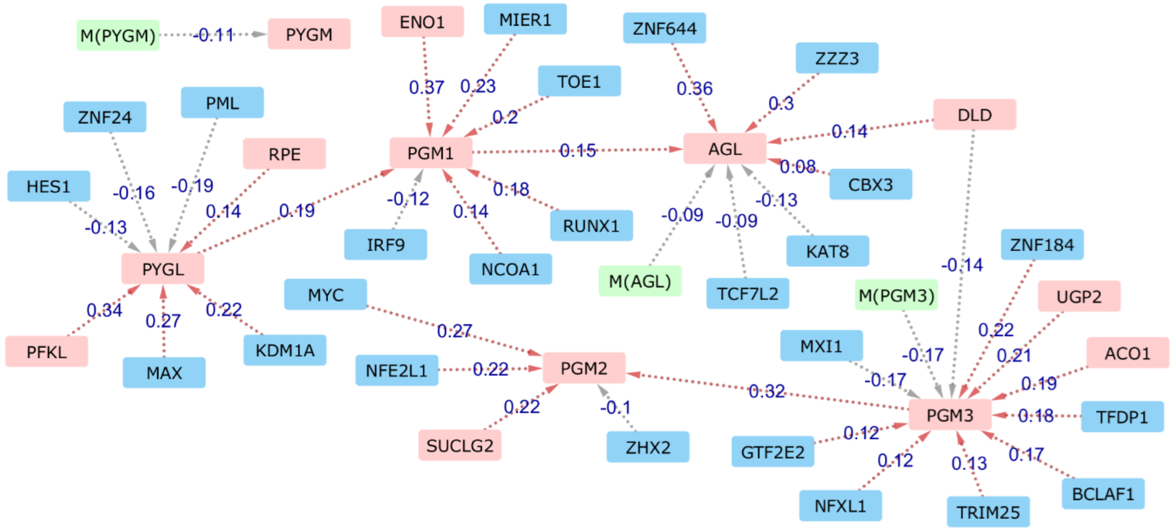

Glycogen Synthesis pathway subclass

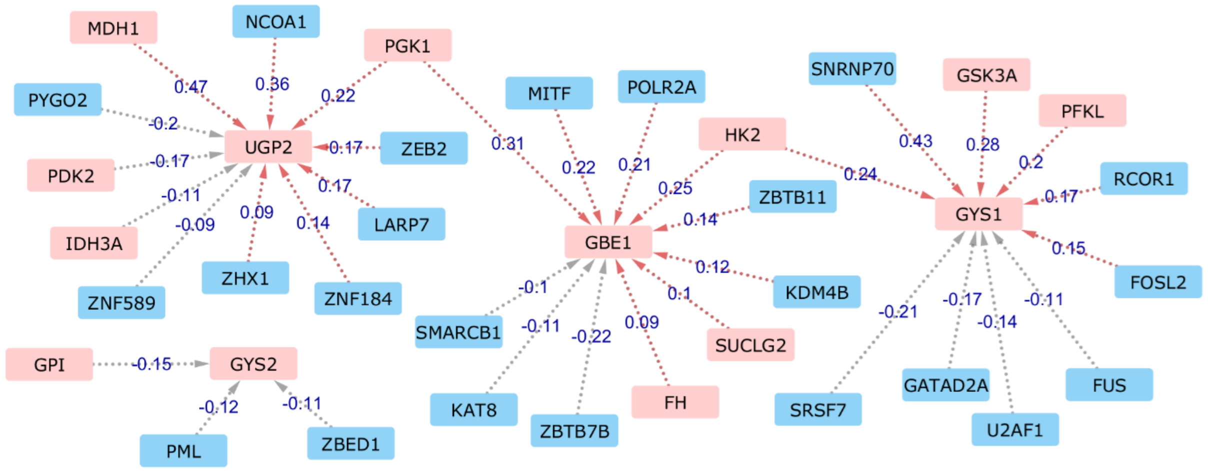

Glycolysis pathway subclass

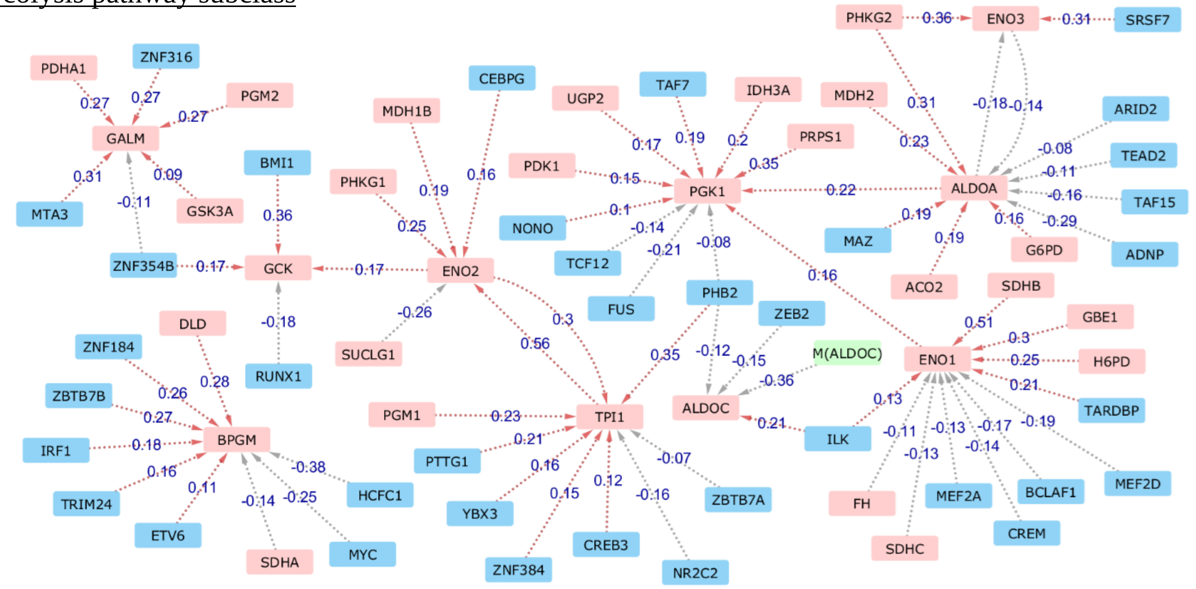

Pentose Phosphate Pathway pathway subclass

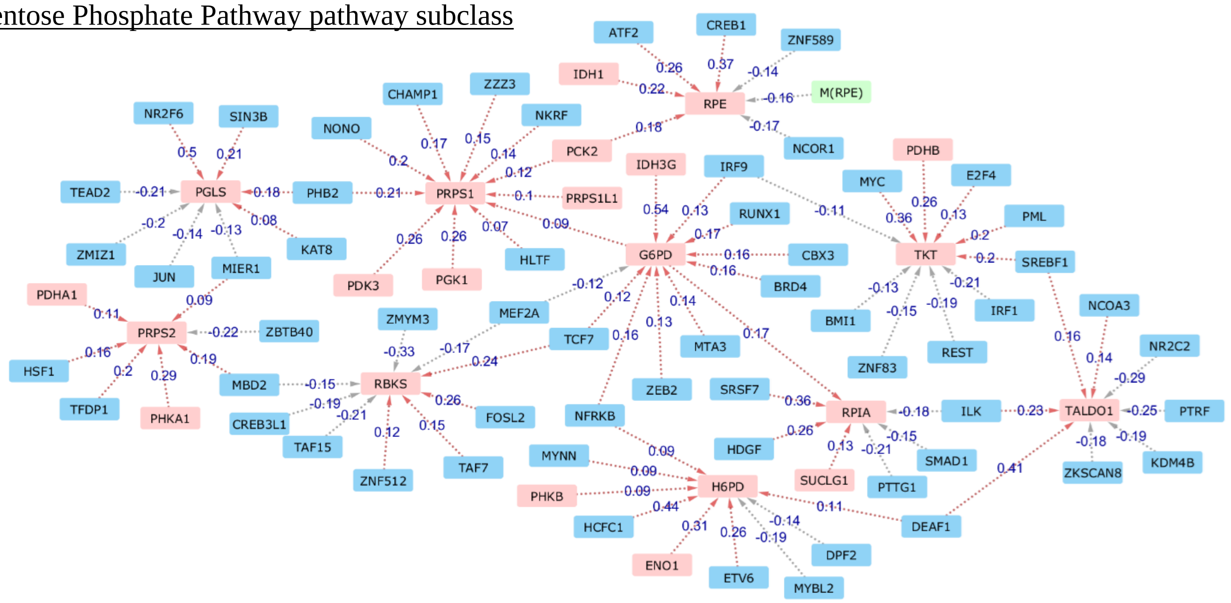

Regulation of Glucose Metabolism pathway subclass

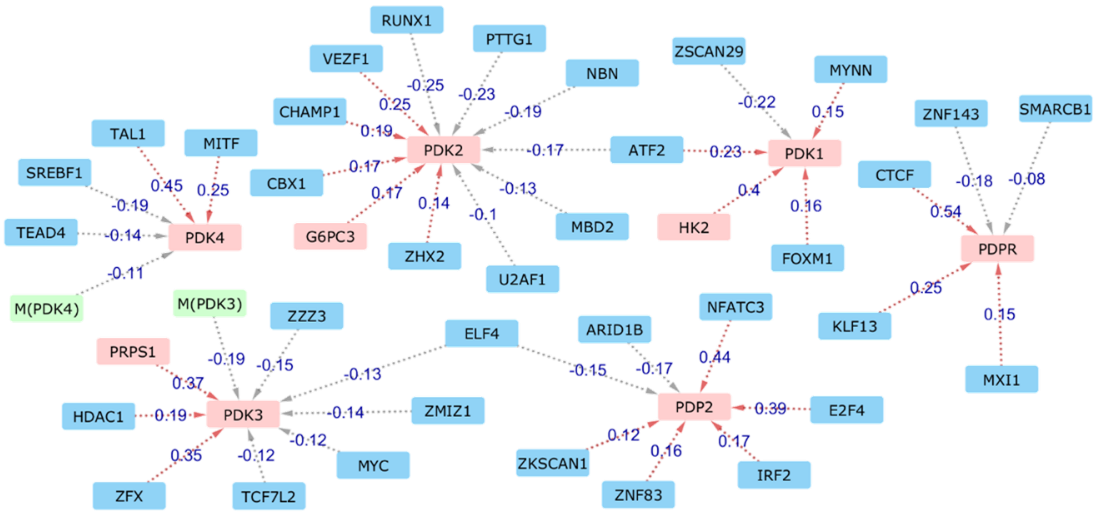

Regulation of Glycogen Metabolism pathway subclass

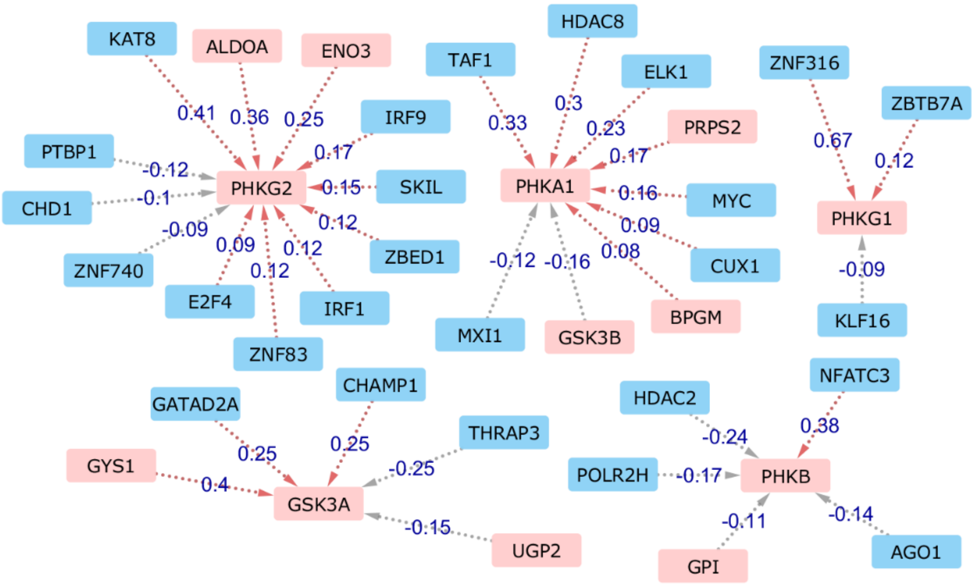

Tricarboxylic Acid Cycle pathway subclass

This network is almost unreadable on paper, due to the high number of genes of the GLUCOSE_METABOLISM pathway belonging to the Tricarboxylic Acid Cycle pathway subclass. The best way to visualize it is using the original network file and opening it with Cytoscape, in order to be able to retrieve all the correlations between the genes set as nodes (available at https://github.com/DEIB-GECO/genereg under *OV Cancer Results /Results Networks.cys*).

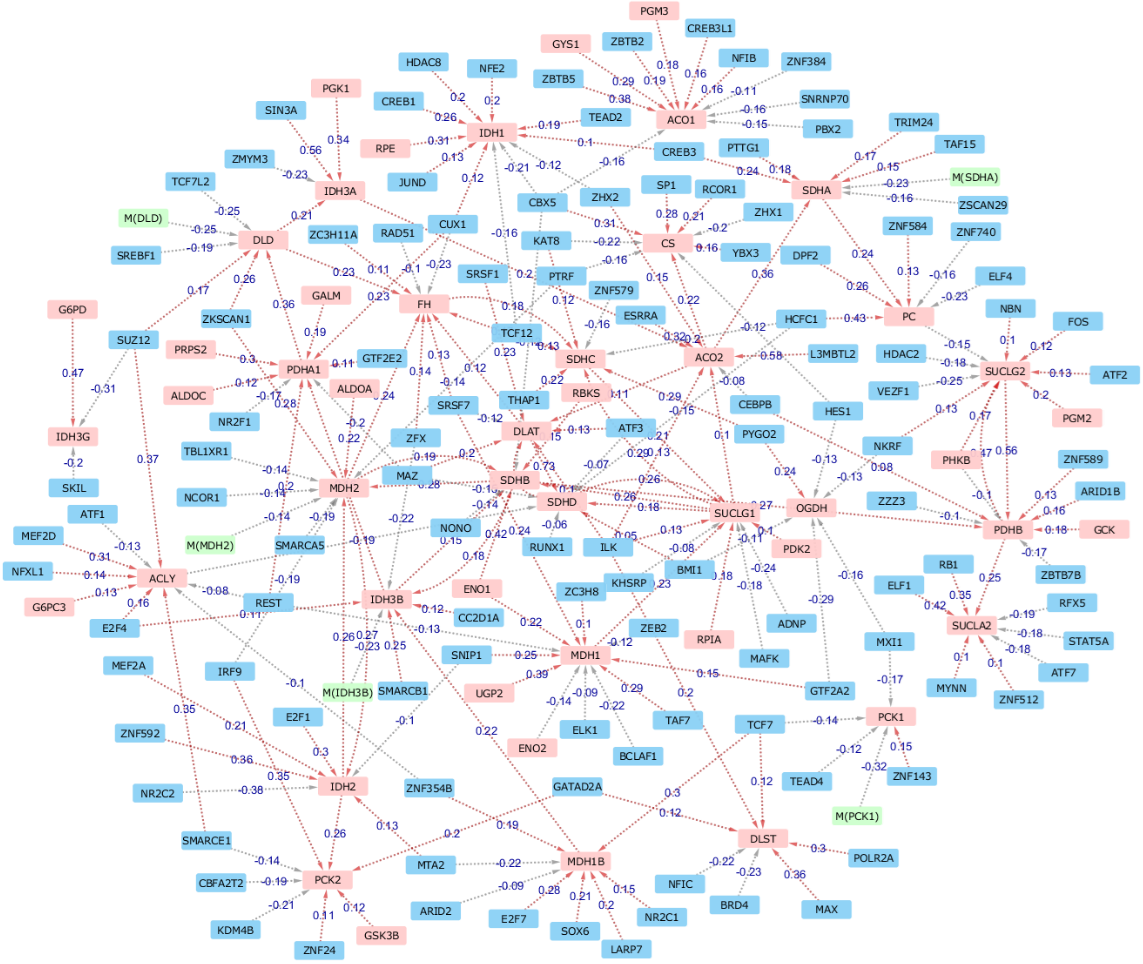

Unclassified pathway subclass

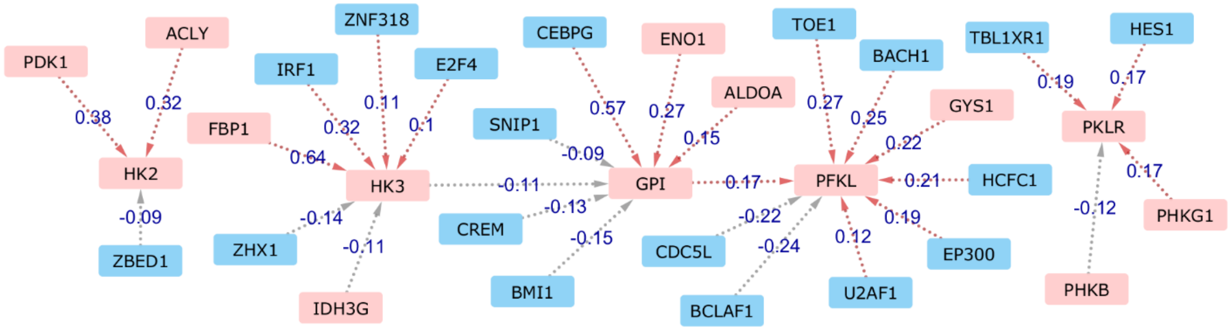

S3.6 GLUCOSE_METABOLISM pathway (M5 models)

Gluconeogenesis pathway subclass

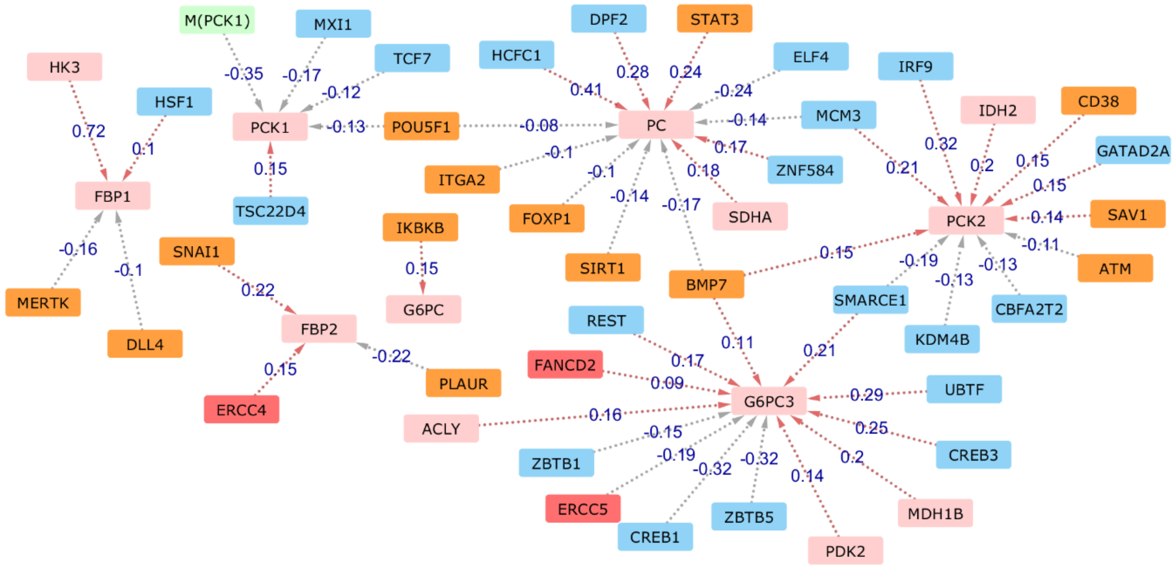

Glycogen Degradation pathway subclass

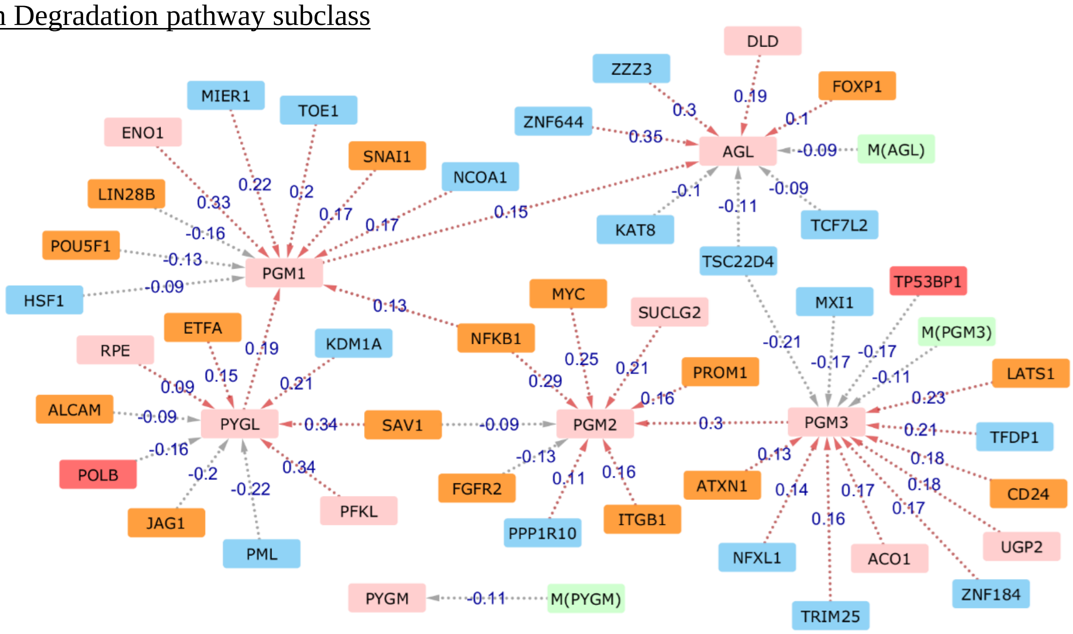

Glycogen Synthesis pathway subclass

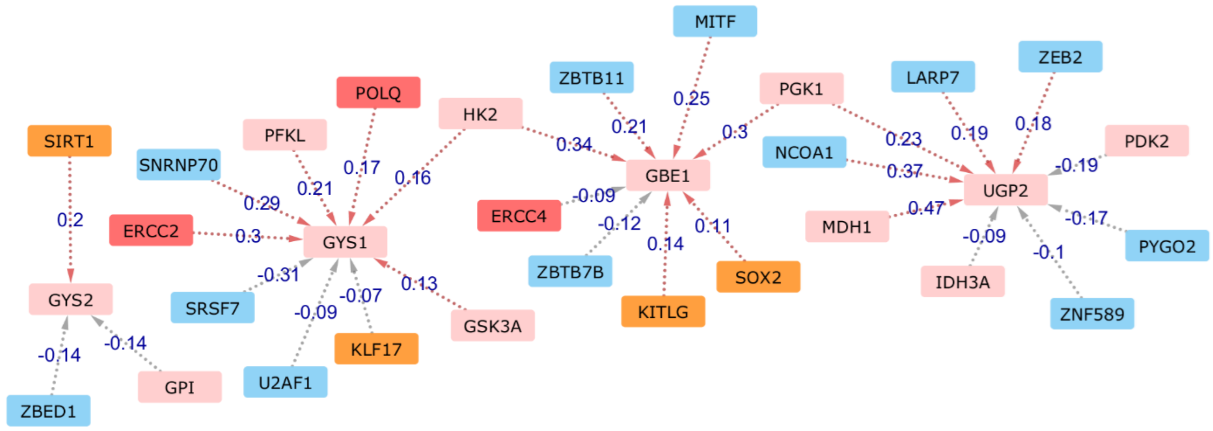

Glycolysis pathway subclass

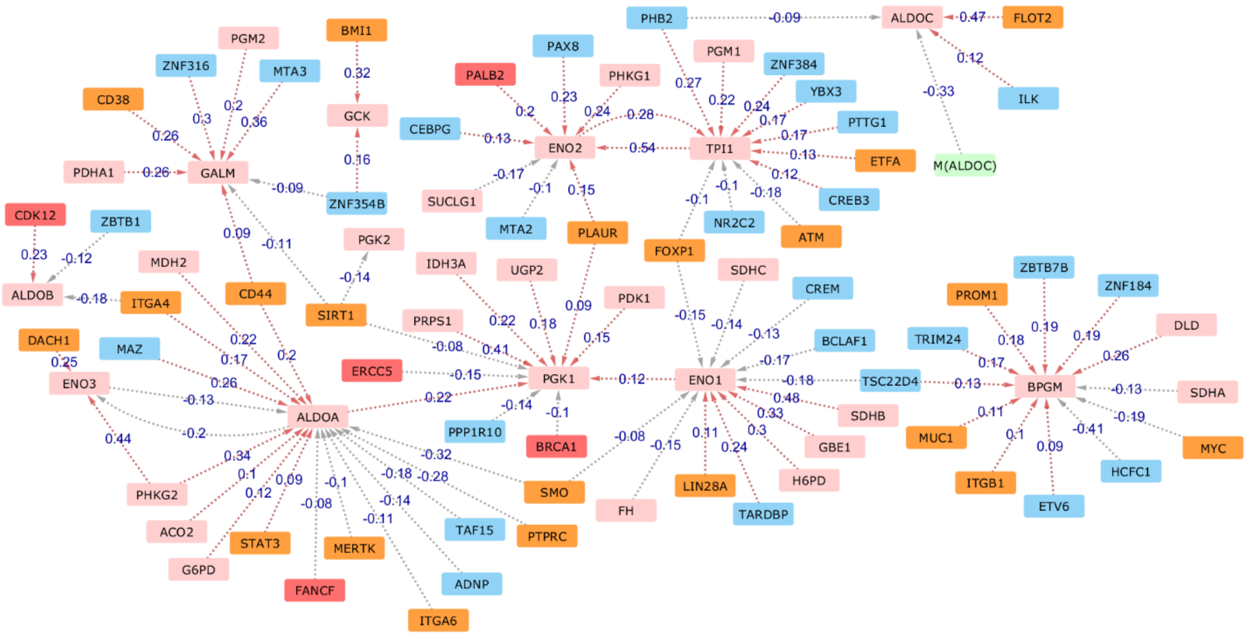

Pentose Phosphate Pathway pathway subclass

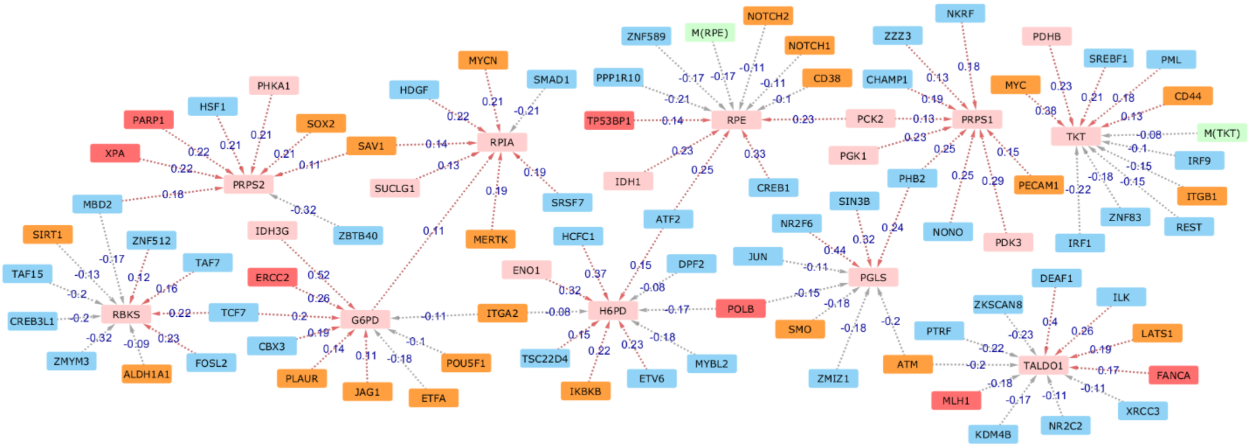

Regulation of Glucose Metabolism pathway subclass

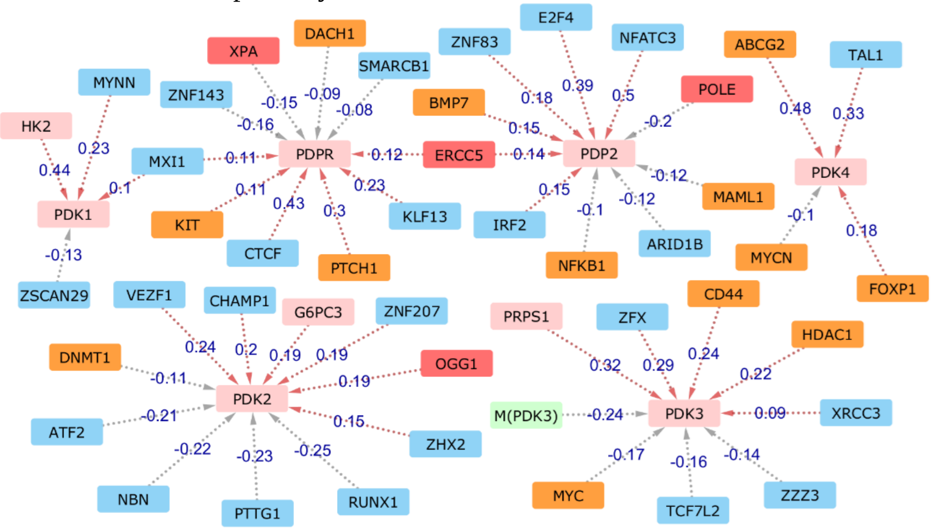

Regulation of Glycogen Metabolism pathway subclass

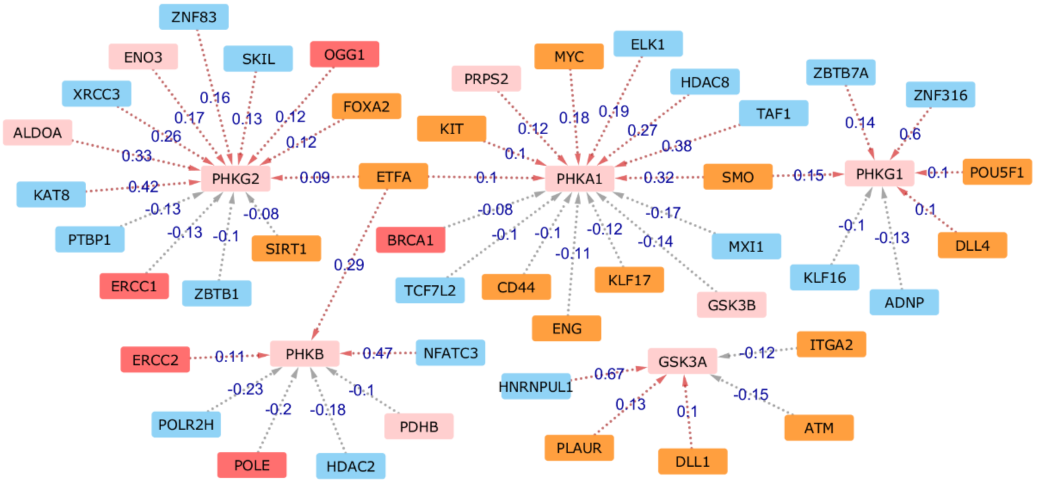

Tricarboxylic Acid Cycle pathway subclass

This network is completely unreadable on paper, thus it is not reported; what said for model M3 holds also for model M5 and its resulting network, which comprises even more genes and a higher number of correlations.

The original network can be visualized with Cytoscape and it is available at https://github.com/DEIB-GECO/genereg under *OV Cancer Results / Results Networks.cys*).

Unclassified pathway subclass

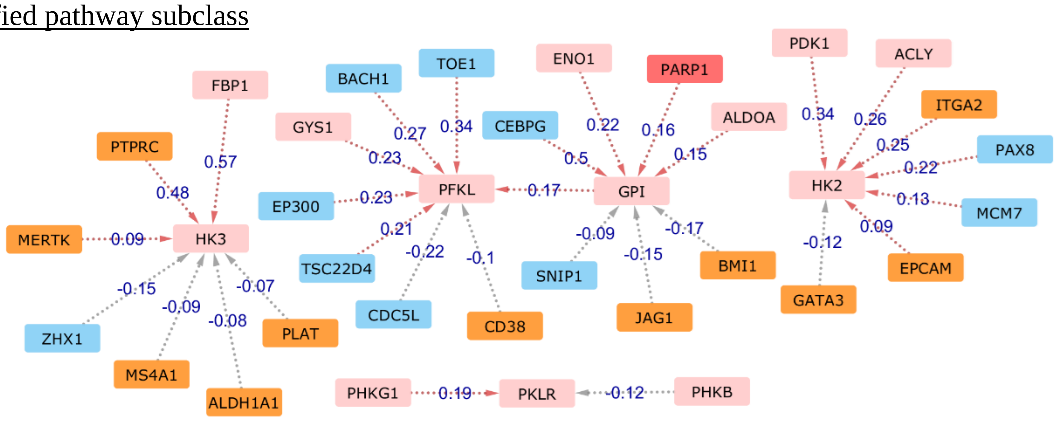

S4. Alternative feature selection methods

S4.1 Feature selection

Our library *genereg* implements 5 different feature selection procedures, offering the user the possibility to select which one to use for the data analysis. The default one is the forward feature selection (FFS) combined with the 5-fold cross-validation process that we propose and describe in the main paper. The first two alternative methods follow the same principles of the default one, but they differ in the way the features selected in previous steps are treated; instead, the last two methods are based on the Lasso algorithm (one with re-evaluation of the features, the other one applied on all features together), combined with the 5-fold cross-validation process. The following subsections present a brief description of how these methods work in detail.

S4.1.1 Incremental forward feature selection with feature re-evaluation

This is the multistep incremental method we propose: in each step (i.e., for each data matrix used), it considers only the features extracted as most relevant in the previous step (if any) and it re-evaluates them with all the new features in the current data matrix during a new feature selection process. In particular, for each target gene and for each input data matrix, the feature selection process is performed five times in each step: in order to reduce possible biases, the set of TCGA data samples is randomly split into five, possibly equal, groups of samples that are used to create five different testing sets. Therefore, for each target gene the feature selection is performed five times, one for each generated testing set (using the remaining samples as training set), according to a k-fold cross-validation process, setting k = 5. The intersection of the five sets of extracted features is computed to obtain the final selected features for the target gene in the considered data matrix.

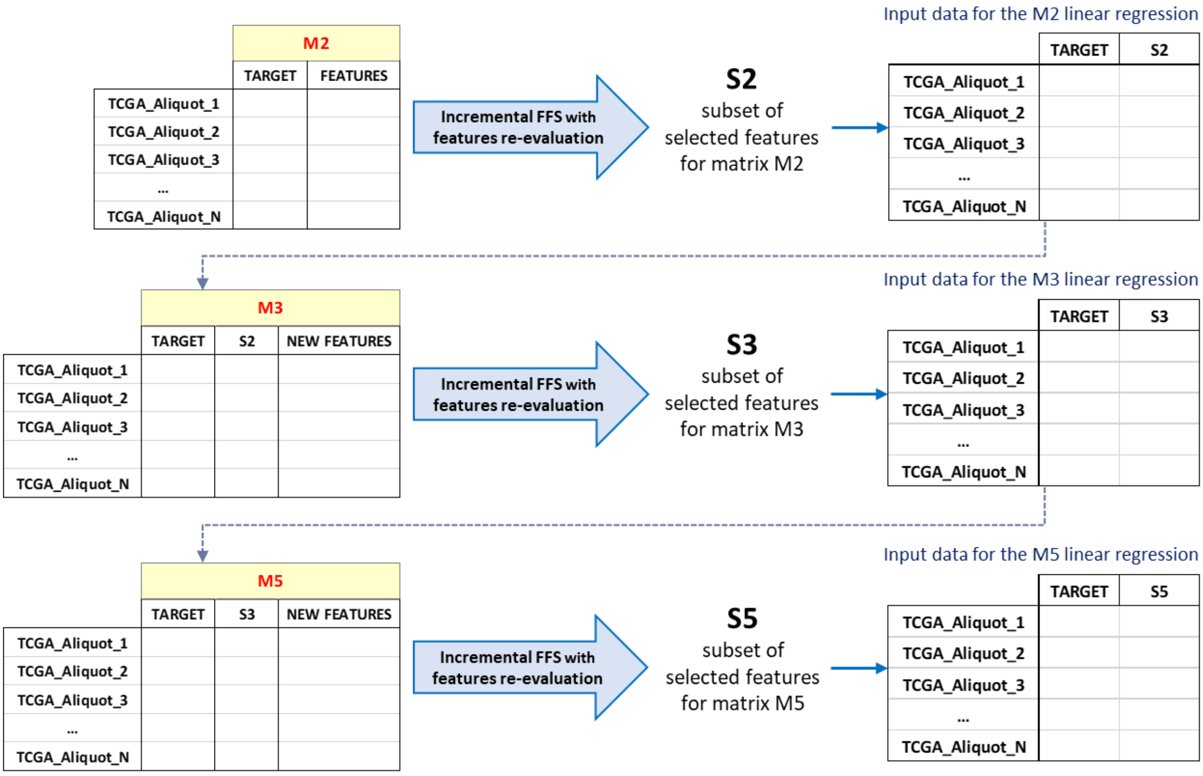

Figure S4.1: Incremental forward feature selection with feature re-evaluation.

S4.1.2 Incremental forward feature selection with no feature re-evaluation

This incremental method is quite similar to the default procedure: it is a FFS with 5-fold cross-validation using the same training and test sets of the default method, but it does not implement the feature re-evaluation process. In each step (i.e., for each data matrix) it considers only the new features added to the current matrix with respect to the previous one, maintaining the features selected in the previous step (if any) as fixed and without re-evaluating them during the new feature selection process. Thus, the set of features selected for each data matrix surely contains all the features selected for the previous data matrix, if any, in case along with new relevant features.

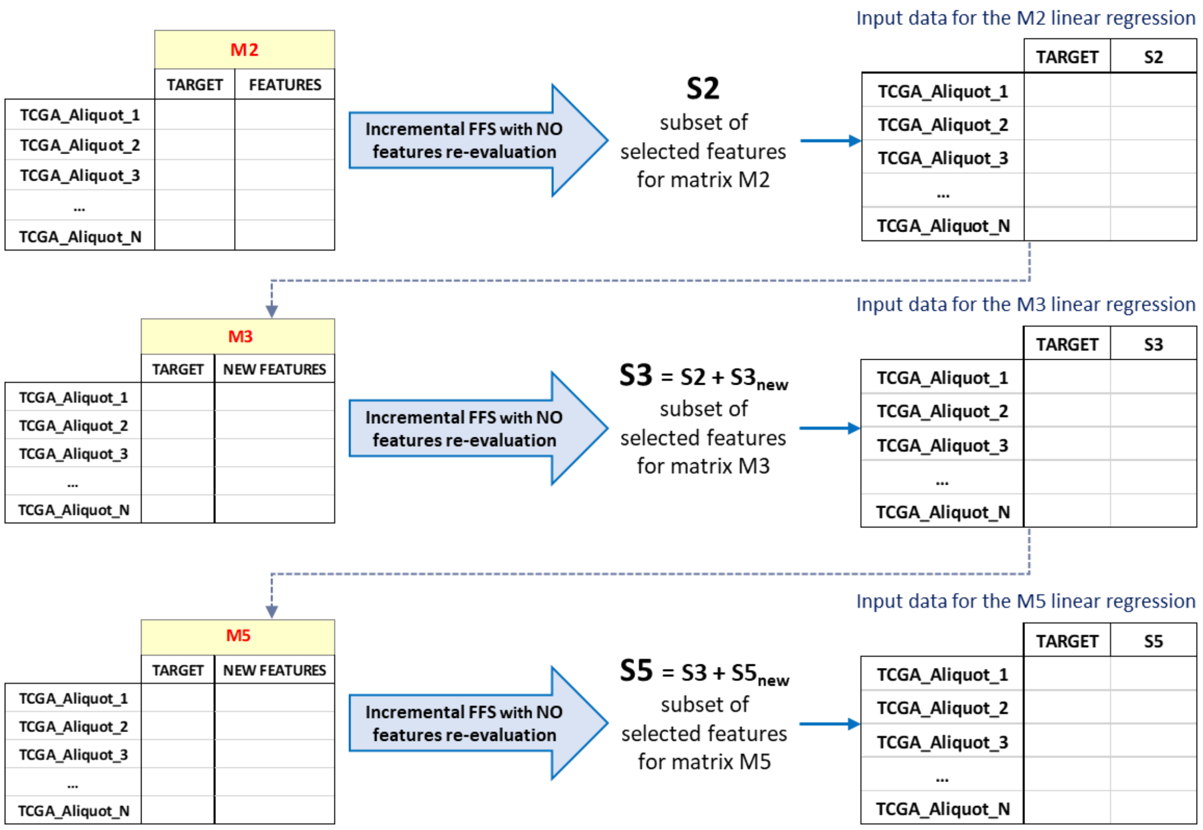

Figure S4.2: Incremental forward feature selection with no feature re-evaluation.

S4.1.3 Forward feature selection on all features

This method is a complete forward feature selection with 5-fold cross-validation, using the same training and test sets of the default method, considering all the features contained in the current data matrix regardless of the ones selected in the previous step. It allows analyzing all the potential regulatory features together, without any previous pre-selection process. Thus, it is particularly useful when applied to the matrix M5, which contains the whole set of candidate regulatory features. This allows building one single regression model for each target gene, considering all its features at once and all together. However, the user needs to pay attention to this type of selection process because the computational complexity (thus the execution time) inevitably increases, in case greatly, due to the high number of input features.

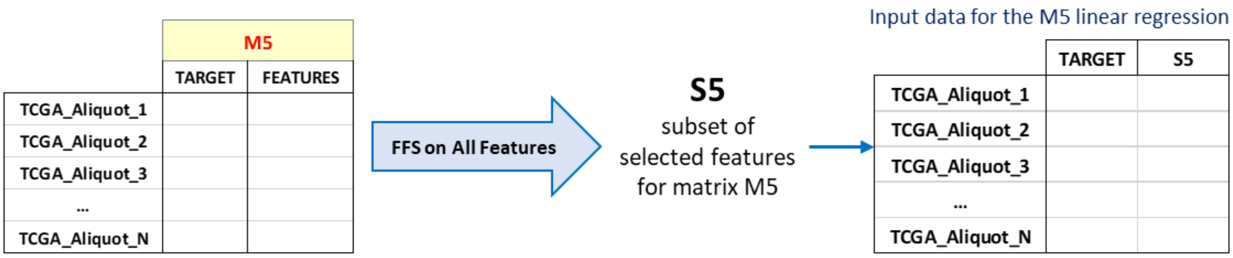

Figure S4.3: Forward feature selection on all features together.

S4.1.4 Incremental Lasso feature selection with feature re-evaluation

This is an incremental method with re-evaluation of the features, very similar to the default one; however, it differs in the algorithm used to identify the relevant features: Lasso instead of forward feature selection, combined with 5-fold cross-validation. Here, the features selected are the ones with a coefficient different from zero, according to the Lasso algorithm, selecting the best tuning parameter (*alpha*). A Lasso regression with multiple possible alpha values is executed and, as a result, the best alpha value is identified; finally, the Lasso regression is performed according to the selected alpha value, in order to extract the most relevant features.
More in details: for each target gene, 5 Lasso feature selection processes are performed, using the same training and test sets of the default method, and the intersection of the five sets of extracted features is computed to obtain the final selected features for the target gene in the considered data matrix. In each of the 5 processes, the best tuning parameter alpha is identified among 10 different candidate values (0.01, 0.02, 0.05, 0.1, 0.2, 0.5, 1, 2, 5, 10) and then that same alpha value is used to perform a Lasso feature selection for linear regression, in order to select relevant features, i.e., the ones with a non-zero regression coefficient.

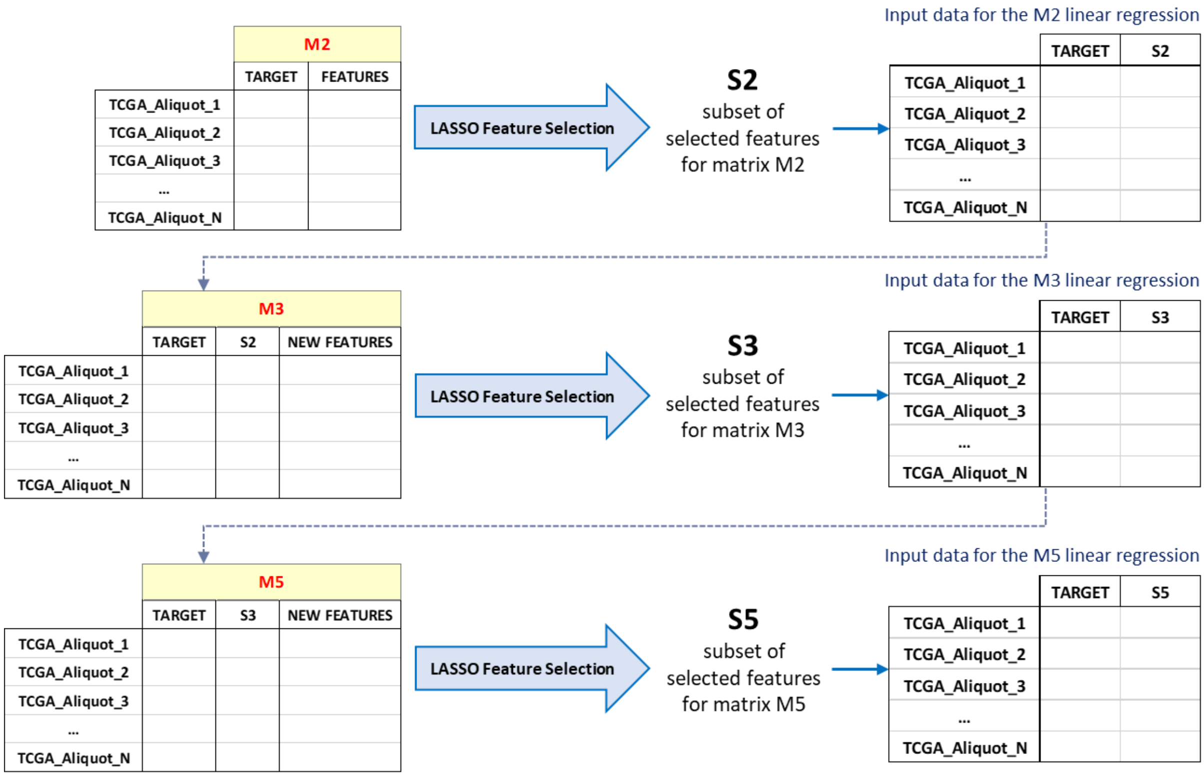

Figure S4.4: Lasso feature selection with feature re-evaluation.

S4.1.5 Lasso feature selection on all features

This method is a complete Lasso feature selection with 5-fold cross-validation using the same training and test sets of the default method, considering all the features contained in the current matrix regardless of the ones selected in the previous step. It allows to analyze all the potential regulatory features together, without any previous pre-selection process. Thus, this method is extremely useful if applied to matrix M5, which contains the whole set of candidate regulatory features. This allows building one single regression model for each target gene, considering all its features at once and all together. Differently from the FFS, Lasso guarantees a very low computational complexity, even if analyzing the whole set of features; thus, it is preferable to FFS when applied to large input matrices.

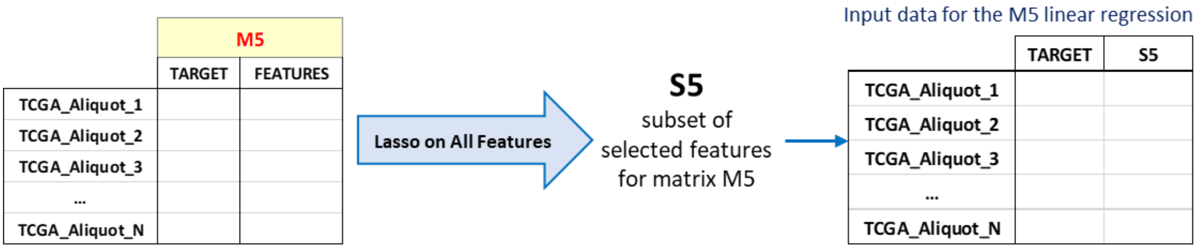

Figure S4.5: Lasso feature selection on all features together.

S4.2 Results and comparisons

In this Section, we report brief comparisons between the results obtained through our default method and using the alternative methods on Ovarian Cancer data.
Pairwise comparison details comprise the following plots:

- a detailed comparison summarizing the results of model M5 for a set of 6 restricted target genes, identified as most relevant and interesting during the evaluation of our default method results for the OV tumor (BRCA1, CDK12, ERCC1, CHEK1, DNMT1, PTPRC), highlighting similarities and differences in the final regression model built in the two compared methods;
- line charts summarizing the number of relevant features selected by the two compared methods and the common ones, for each OV genomic pathway;
- mean and confidence interval plots for each OV genomic pathway in the two compared methods;
- Fisher's exact test summary, considering the number of features extracted by the two compared methods, in order to assess the significance of the distribution of these values and whether this number is significantly different between the two methods, building a contingency table and computing the *p-value* as follows:

	Method 1	
Method 2	*a*	*b*
	c	*d*

Method 1 = our default method
Method 2 = alternative method
a = features extracted in both method 1 and method 2
b = features extracted in method 2, but not in method 1
c = features extracted in method 1, but not in method 2
d = features extracted neither in method 1 nor in method 2
(always considering only features extracted from method 1 and/or 2, so this always equals 0)

Resulting *p-value*: $\boldsymbol{p} = [(a + b)!\ (c + d)!\ (a + c)!\ (b + d)!] / [a!\ b!\ c!\ d!\ (a + b + c + d)!]$

Overall, all performed comparisons show that a relevant number of the features that our method selects are selected also by the other methods, which generally select more features but not in a statistically significant higher amount. The forward feature selection method applied on all features together was the only exception, resulting selecting statistically significantly less features than our approach; furthermore, it was the only one that did not identify the relationship between the DNMT1 and CHEK1 genes, although it has been confirmed in OV cancer PDX models and in a model of conditional CHECK1 knock-out in HCT116 cultured ovarian carcinoma cells; all other relationships experimentally confirmed that we tested (see main paper *Results* subsection *Experimental validation of found biological correlations in independent Ovarian and Breast cancer samples*) were identified by all considered methods.

S4.2.1 Our incremental FFS method vs. FFS on all features

- BRCA1 (Adjusted R^2_{M5} = 0.59 vs. 0.62): all the relevant features identified by our default method, except two of them (CBX5 and POLQ), are confirmed by this first alternative method, including gene methylation, with regression coefficients that are highly similar in both methods (the most relevant feature is the same in both methods, FANCC):

**Common Features**	**Coefficient (Our Method)**	**Coefficient (FFS on All Features)**
FANCC	0.2336	0.2405
SUZ12	0.2317	0.2100
ZBTB2	0.1160	0.1868
METHYLATION (BRCA1)	-0.3575	-0.3370

  The alternative method identifies 6 new relevant features, excluded by the default method: FANCD2 (0.2724), HDAC1 (-0.0847), HDAC2 (0.1074), OGG1 (-0.1029), SKIL (-0.0743) and SMARCE1 (0.1092).

- CDK12 (Adjusted R^2_{M5} = 0.43 vs. 0.03): there are no common relevant features in this case. The default method identifies 4 relevant features, ATM (0.1953), NCOA3 (0.2160), STAT3 (0.2293) and SUZ12 (0.3026), while the alternative one only one single feature, ZMIZ1 (0.1890).

- ERCC1 (Adjusted R^2_{M5} = 0.60 vs. 0.52): 3 relevant features identified by our default method (10 total features) are confirmed by this first alternative method (7 total features), with similar regression coefficients (the most relevant feature is the same in both methods, ERCC2):

**Common Features**	**Coefficient (Our Method)**	**Coefficient (FFS on All Features)**
ERCC2	0.5420	0.4665
HNRNPUL1	0.2743	0.3395
SKIL	-0.1874	-0.1814

  These results confirm the strong relationship between ERCC1 and ERCC2 (experimentally confirmed in OV and BRCA PDX models), identifying ERCC2 as the most relevant feature in both methods, playing a key role in the expression regulation of gene ERCC1.

- CHEK1 (Adjusted R^2_{M5} = 0.66 vs. 0.64): 5 relevant features identified by our default method (12 total features) are confirmed by this first alternative method (8 total features), with the following regression coefficients (the most relevant feature is the same in both methods, NFRKB):

Common Features	Coefficient (Our Method)	Coefficient (FFS on All Features)
NFRKB	0.4343	0.5139
RAD51	0.2605	0.3062
MAFK	-0.1611	-0.2237
RB1	-0.1680	-0.2861
ZKSCAN1	-0.1872	-0.2104

However, the alternative method does not select the methylation of CHEK1 as one of its relevant features, differently from what happens by applying the default method (methylation is selected with a regression coefficient of -0.1144).

- DNMT1 (Adjusted R^2_{M5} = 0.70 vs. 0.62): 3 relevant features identified by our default method (8 total features) are confirmed by this first alternative method (4 total features), with the following regression coefficients (the most relevant feature is the same in both methods, SMARCA4):

Common Features	Coefficient (Our Method)	Coefficient (FFS on All Features)
SMARCA4	0.5505	0.6402
POLE	0.1738	0.3492
TEAD2	-0.1282	-0.1151

However, CHEK1 is not identified by the alternative method, despite the relationship between DNMT1 and CHEK1 has been confirmed in OV cancer PDX models and in a model of conditional CHECK1 knock-out in HCT116 cultured ovarian carcinoma cells (see main paper *Results* subsection *Experimental validation of found biological correlations in independent Ovarian and Breast cancer samples*).

- PTPRC (Adjusted R^2_{M5} = 0.82 vs. 0.82): all the relevant features identified by our default method, except two of them (AXL and ERCC2), are confirmed by this first alternative method, with similar regression coefficients (the most relevant feature is the same in both methods, IKZF1):

Common Features	Coefficient (Our Method)	Coefficient (FFS on All Features)
IKZF1	0.5857	0.6340
ITGA4	0.2392	0.2328
CD38	0.1394	0.1075
CD44	0.1217	0.0922
ELF4	-0.1252	-0.1578

The alternative method identifies only 1 new relevant feature, excluded by the default method: IRF1 (0.1114).

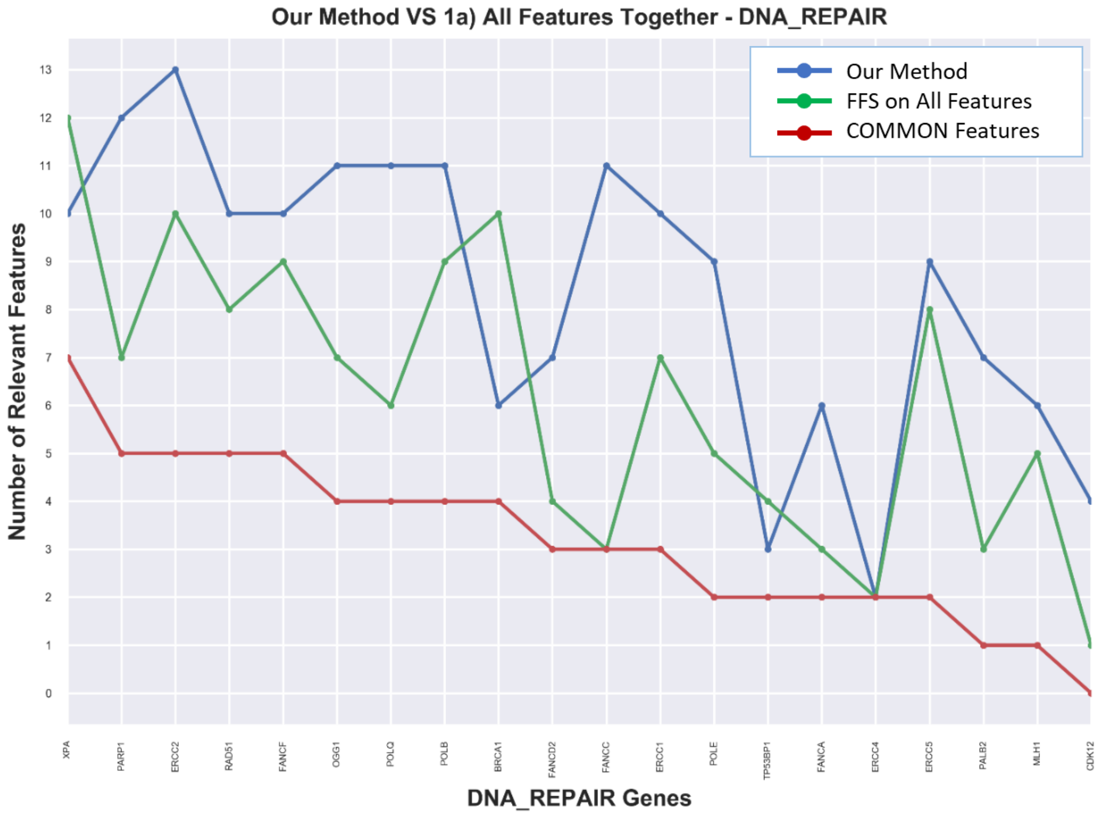

Figure S4.6: Number of relevant features selected by the two methods in the DNA_REPAIR pathway (Our Default Method vs. FFS All Features).

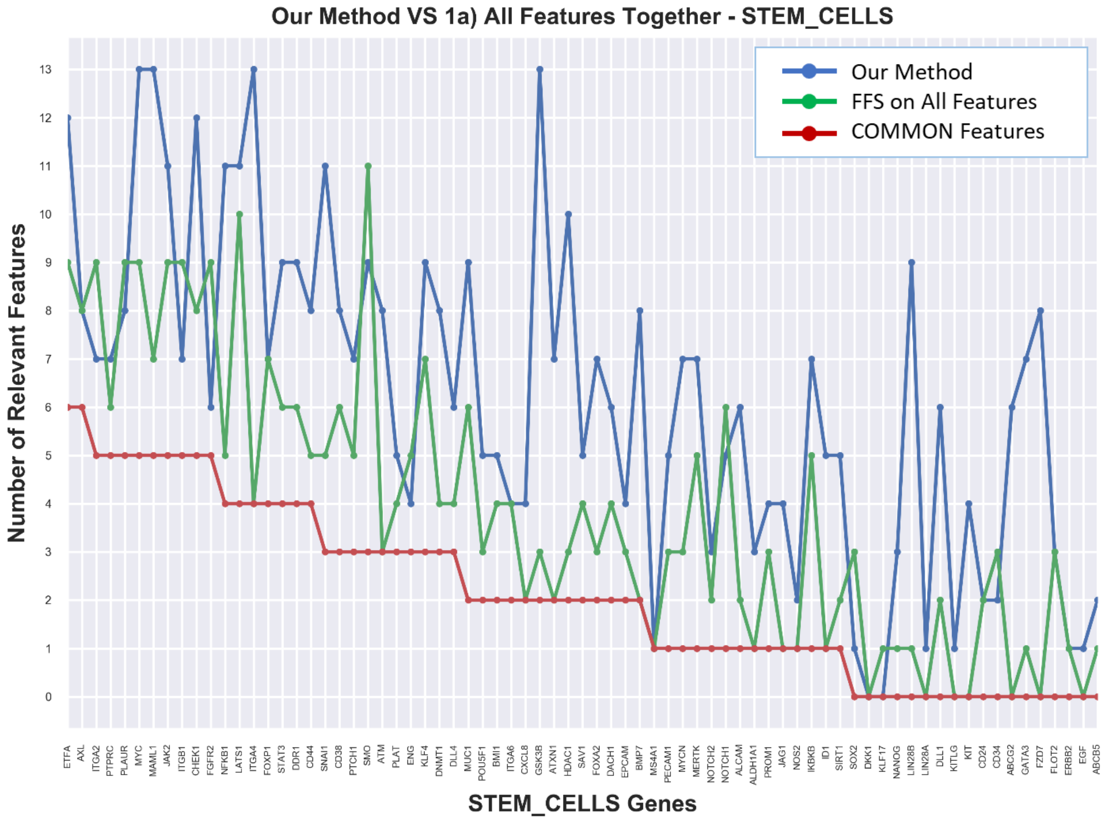

Figure S4.7: Number of relevant features selected by the two methods in the STEM_CELLS pathway (Our Default Method vs. FFS All Features).

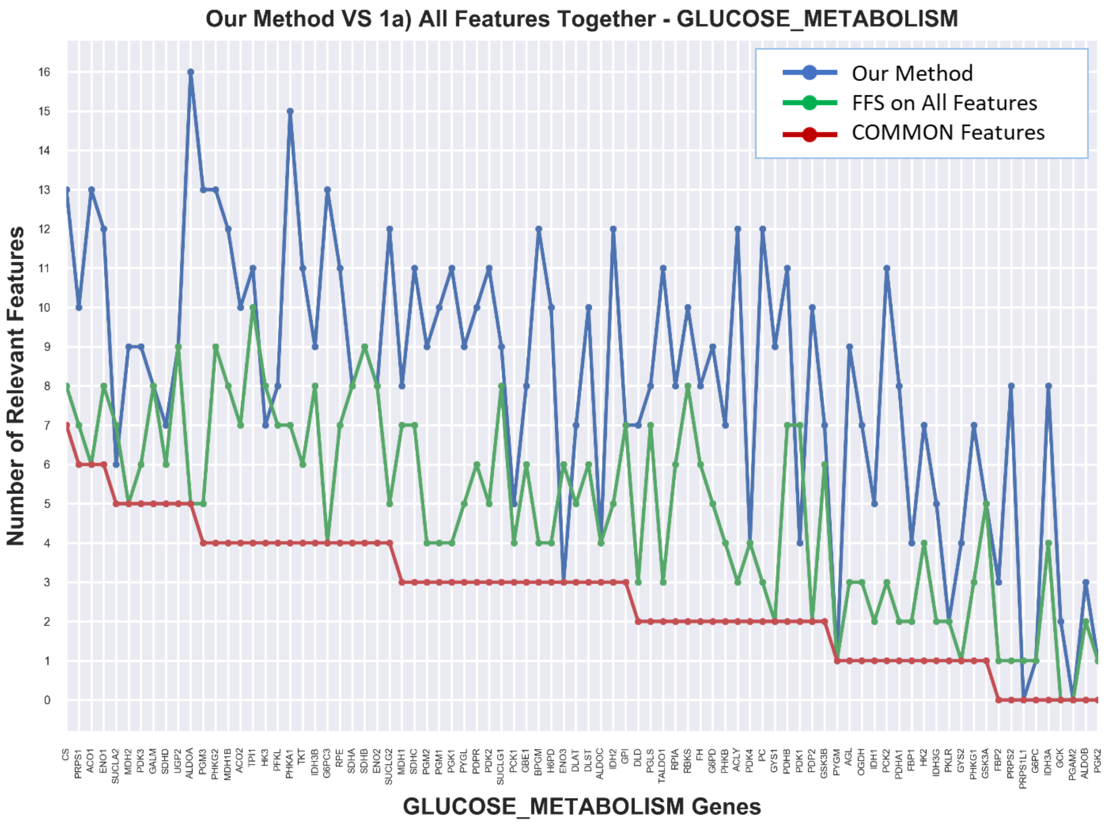

Figure S4.8: Number of relevant features selected by the two methods in the GLUCOSE_METABOLISM pathway (Our Default Method vs. FFS All Features).

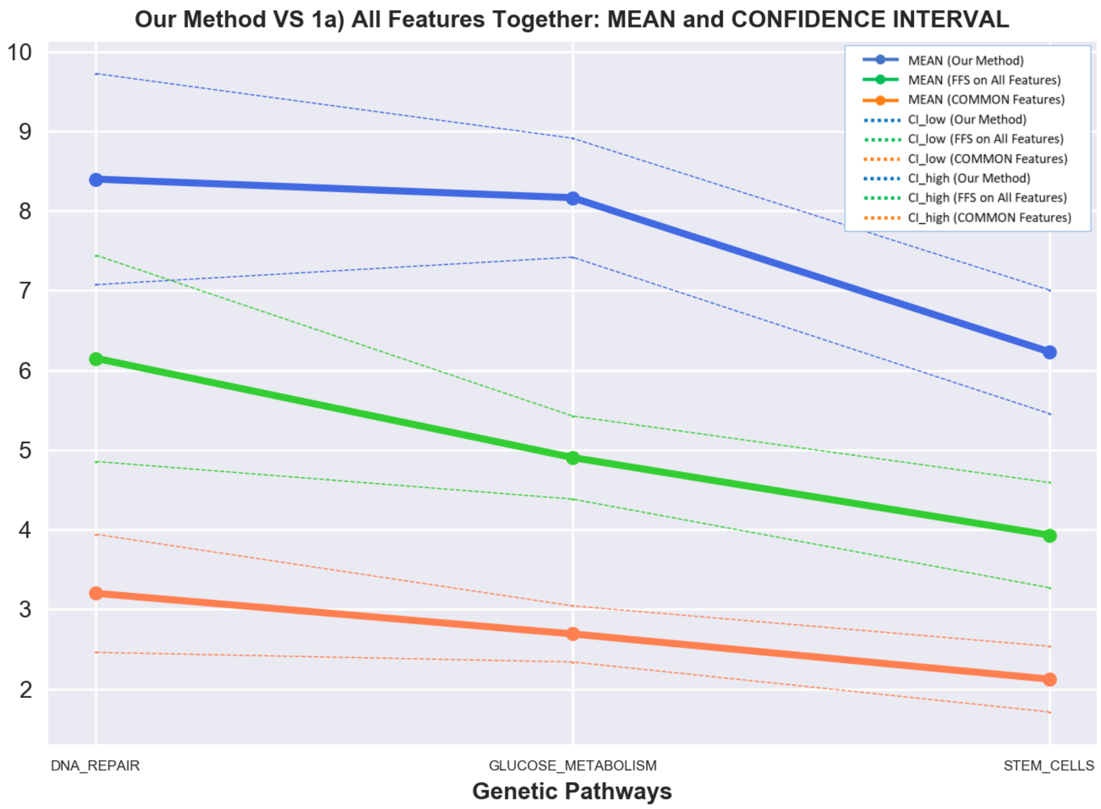

Figure S4.9: Mean and confidence interval plots of the number of relevant features selected by the two methods in the three pathways (Our Default Method vs. FFS All Features).

	Our Default Method	
FFS All Features	319	25
	64	0

$p \approx 0.01$

Figure S4.10: Fisher's exact test summary for all features extracted by the two methods (Our Default Method vs. FFS All Features).

S4.2.2 Our incremental FFS method vs. incremental FFS with no feature re-evaluation

- BRCA1 (Adjusted R^2_{M5} = 0.59 vs. 0.64): all the relevant features identified by our default method, except one (CBX5), are confirmed by this second method, including gene methylation, with the following regression coefficients (the most relevant feature is the same in both methods, FANCC):

Common Features	Coefficient (Our Method)	Coefficient (Incr. FFS with NO re-eval)
FANCC	0.2336	0.2375
SUZ12	0.2317	0.1753
POLQ	0.2288	0.1798
ZBTB2	0.1160	0.1308
METHYLATION (BRCA1)	-0.3575	-0.3388

The alternative method identifies 4 new relevant features, excluded by the default method: DDR1 (-0.1023), SRSF1 (0.1573), STAT3 (0.1579), and ZNF354B (-0.0940).

- CDK12 (Adjusted R^2_{M5} = 0.43 vs. 0.53): all the relevant features identified by our default method are confirmed by this alternative method, with the following regression coefficients:

Common Features	Coefficient (Our Method)	Coefficient (Incr. FFS with NO re-eval)
SUZ12	0.3026	0.1588
STAT3	0.2293	0.1613
NCOA3	0.2160	0.2279
ATM	0.1953	0.1235

A point to stress in this case is the different behavior of these two methods with respect to the gene methylation, which is not identified as relevant by the default method, but it is selected in the alternative one (with coefficient -0.1380).

- ERCC1 (Adjusted R^2_{M5} = 0.60 vs. 0.61): all the relevant features identified by our default method, except three of them (E2F4, NCO3 and PTTG1), are confirmed by this method, with the following regression coefficients (the most relevant feature is the same in both methods, ERCC2):

Common Features	Coefficient (Our Method)	Coefficient (Incr. FFS with NO re-eval)
ERCC2	0.5420	0.4225
HNRNPUL1	0.2743	0.2373
ZHX1	-0.1278	-0.0983
POLR2A	-0.1605	-0.1345
RCOR1	-0.1712	-0.1574
SKIL	-0.1874	-0.1266
PTBP1	-0.2344	-0.2085

Also in this case, this confirms the strong relationship between ERCC1 and ERCC2 (experimentally confirmed in OV and BRCA PDX models), identifying ERCC2 as the most relevant feature in both methods, playing a key role in the expression regulation of gene ERCC1. However, there is a different behavior with respect to the gene methylation, which is not identified as relevant by the default method, but it is selected in the alternative one (with coefficient -0.0926).

- CHEK1 (Adjusted R^2_{M5} = 0.66 vs. 0.67): 6 relevant features identified by our default method (12 total features) are confirmed by this alternative method (13 total features), including gene methylation, with the following regression coefficients (the most relevant feature is the same in both methods, NFRKB):

Common Features	Coefficient (Our Method)	Coefficient (Incr. FFS with NO re-eval)
NFRKB	0.4343	0.3640
RAD51	0.2605	0.2721
FANCA	0.2058	0.0992
CBX5	0.1290	0.1209
RB1	-0.1680	-0.2004
METHYLATION (CHEK1)	-0.1144	-0.1127

- DNMT1 (Adjusted R^2_{M5} = 0.70 vs. 0.66): 5 relevant features identified by our default method (8 total features) are confirmed by this alternative method (8 total features), with the following regression coefficients (the most relevant feature is the same in both methods, SMARCA4):

Common Features	Coefficient (Our Method)	Coefficient (Incr. FFS with NO re-eval)
SMARCA4	0.5505	0.5431
CHEK1	0.1565	0.2242
MCM3	0.1049	0.1340
DEAF1	-0.1112	-0.0907
TEAD2	-0.1282	-0.0921

Both methods identify CHEK1 as one of the most relevant features, confirming the relevance of the DNMT1-CHEK1 biological relationship, which has been confirmed in OV cancer PDX models and in a model of conditional CHECK1 knock-out in HCT116 cultured ovarian carcinoma cells (see main paper Results subsection *Experimental validation of found biological correlations in independent Ovarian and Breast cancer samples*).

- PTPRC (Adjusted R^2_{M5} = 0.82 vs. 0.85): all the relevant features identified by our default method, except one (ERCC2), are confirmed by this second alternative method, with the following regression coefficients (the most relevant feature is the same in both methods, IKZF1):

Common Features	Coefficient (Our Method)	Coefficient (Incr. FFS with NO re-eval)
IKZF1	0.5857	0.5113
ITGA4	0.2392	0.2020
CD38	0.1394	0.0900
CD44	0.1217	0.0847
AXL	0.0729	0.1116
ELF4	-0.1252	-0.1416

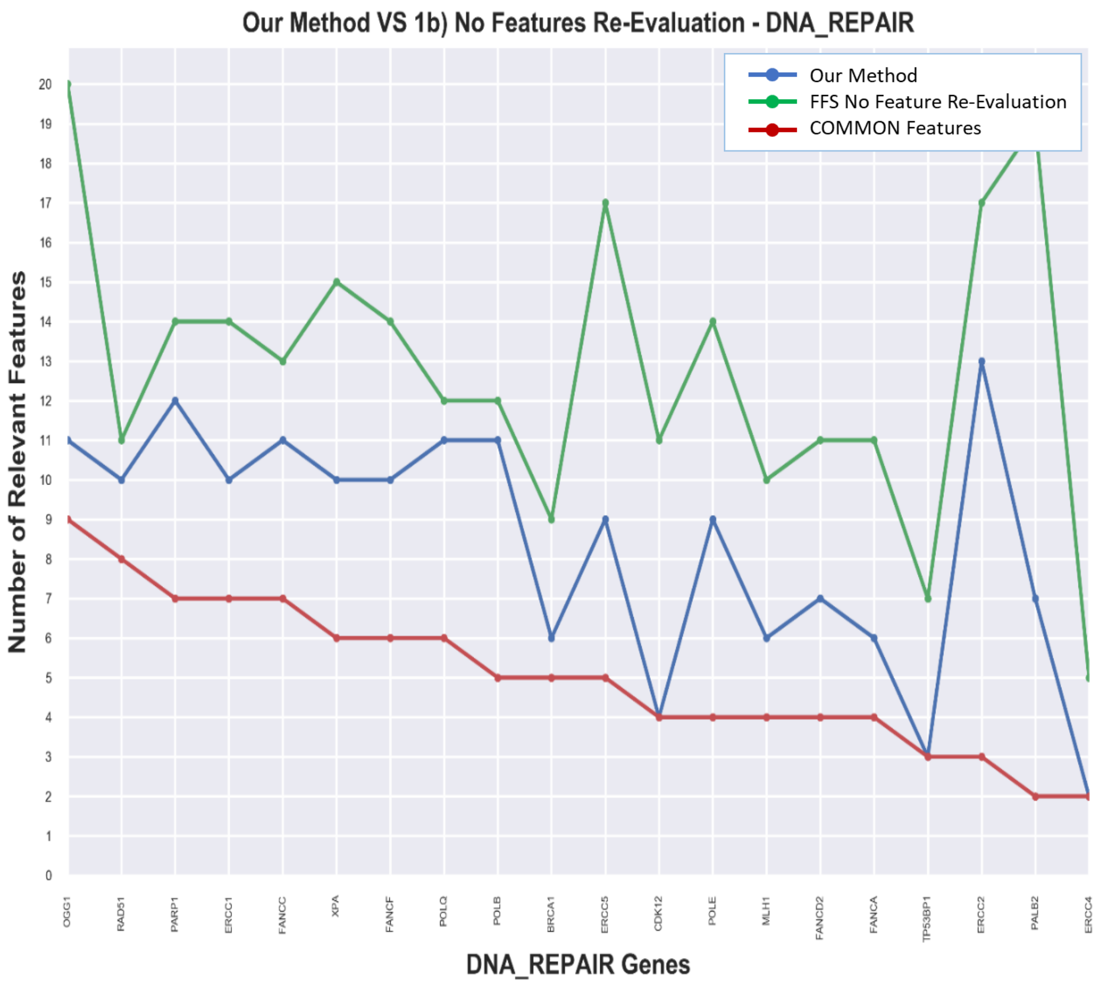

Figure S4.11: Number of relevant features selected by the two methods in the DNA_REPAIR pathway (Our Default Method vs. Incremental FFS No Feature Re-evaluation).

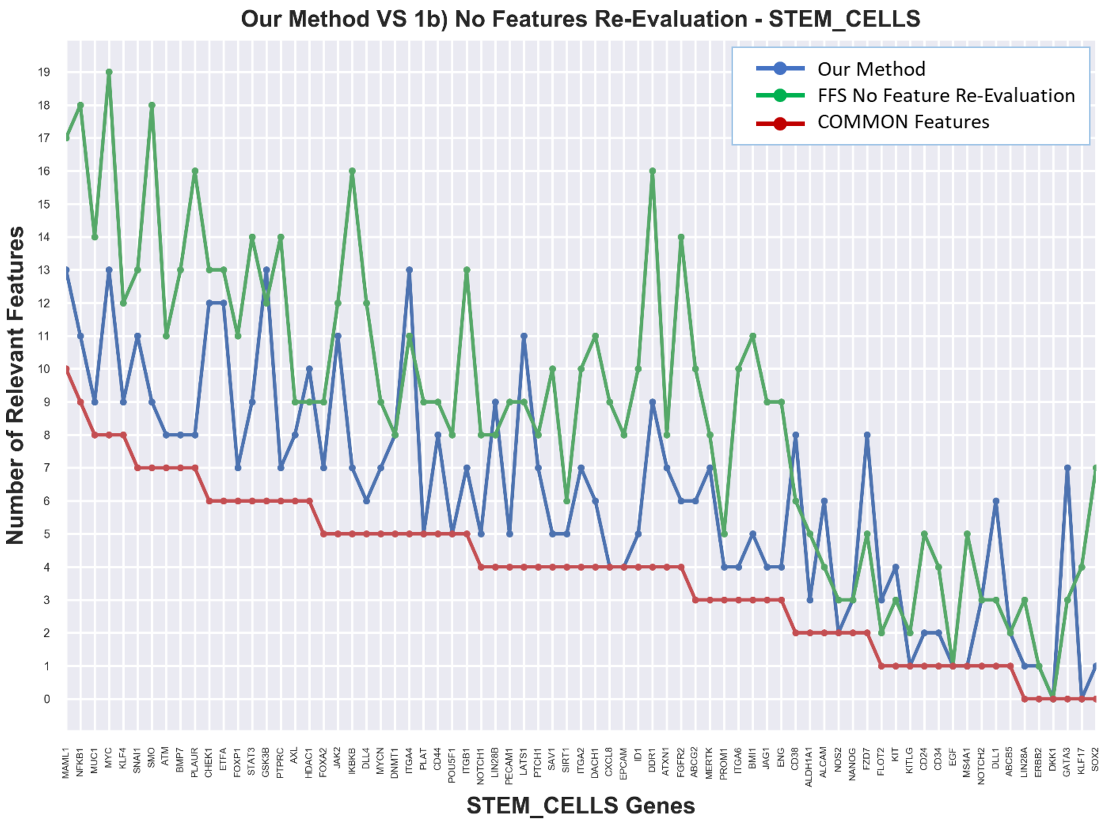

Figure S4.12: Number of relevant features selected by the two methods in the STEM_CELLS pathway (Our Default Method vs. Incremental FFS No Feature Re-evaluation).

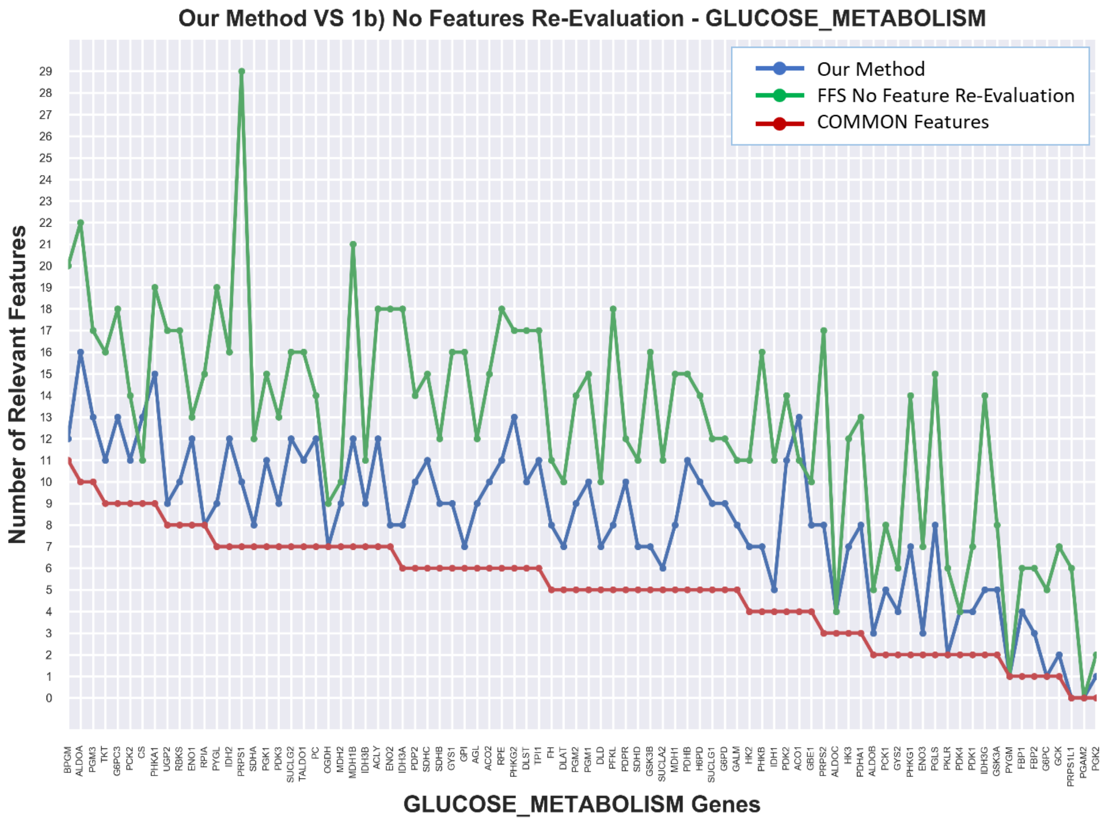

Figure S4.13: Number of relevant features selected by the two methods in the GLUCOSE_METABOLISM pathway (Our Default Method vs. Incremental FFS No Feature Re-evaluation).

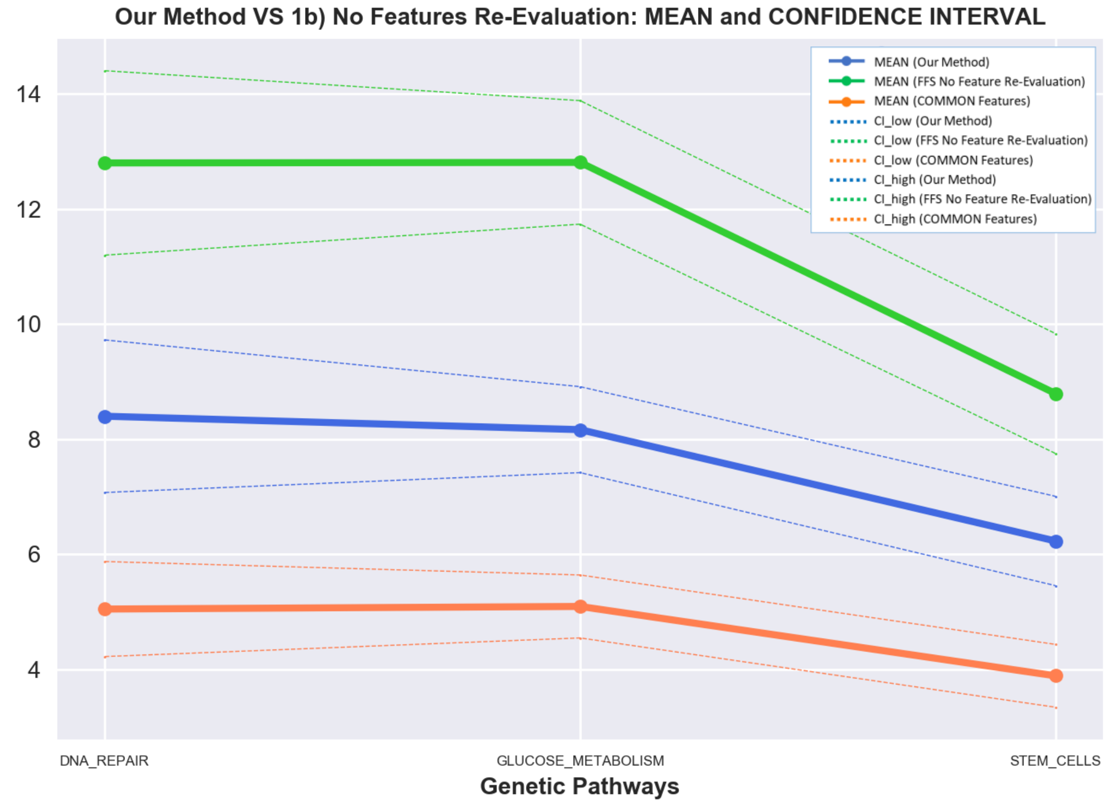

Figure S4.14: Mean and confidence interval plots of the number of relevant features selected by the two methods in the three pathways (Our Default Method vs. Incremental FFS No Feature Re-evaluation).

	Our Default Method	
Incremental FFS No Feature Re-Evaluation	376	49
	7	0

$p \approx 0.43$

Figure S4.15: Fisher's exact test summary for all features extracted by the two methods (Our Default Method vs. Incremental FFS No Feature Re-evaluation).

S4.2.3 Our incremental FFS method vs. incremental Lasso with feature re-evaluation

- BRCA1 (Adjusted R^2_{M5} = 0.59 vs. 0.64): all the relevant features identified by our default method, except two of them (CBX5 and SUZ12), are confirmed by this alternative method, including gene methylation, with the following regression coefficients:

Common Features	Coefficient (Our Method)	Coefficient (Lasso with feature re-eval)
FANCC	0.2336	0.1558
POLQ	0.2288	0.1551
ZBTB2	0.1160	0.1230
METHYLATION (BRCA1)	-0.3575	-0.3355

The alternative method identified 5 new relevant features, excluded by the default method: E2F7 (0.1354), FANCD2 (0.1097), HLTF (0.1022), SMARCE1 (0.1508) and STAT3 (0.1753).

- CDK12 (Adjusted R^2_{M5} = 0.43 vs. 0.44): all the relevant features identified by our default method, except 2 of them (ATM and NCOA3), are confirmed by this alternative method, with the following regression coefficients (the most relevant feature is the same in both methods, SUZ12):

Common Features	Coefficient (Our Method)	Coefficient (Lasso with feature re-eval)
SUZ12	0.3026	0.3245
STAT3	0.2293	0.2190

Differently from the other alternative methods, this one does not identify the gene methylation as a relevant feature, just like the default method.

- ERCC1 (Adjusted R^2_{M5} = 0.60 vs. 0.66): 5 relevant features identified by our default method (10 total features) are confirmed by this alternative method (17 total features), with the following regression coefficients (the most relevant feature is the same in both methods, ERCC2):

Common Features	Coefficient (Default Method)	Coefficient (Lasso with feature re-eval)
ERCC2	0.5420	0.4570
HNRNPUL1	0.2743	0.1990
NCOA3	-0.0821	-0.1053
E2F4	-0.1561	-0.1656
SKIL	-0.1874	-0.1072

This confirms the strong relationship between ERCC1 and ERCC2 (experimentally confirmed in OV and BRCA PDX models), with ERCC2 playing a key role in the expression regulation of gene ERCC1. However, there is a different behavior with respect to the gene methylation, which is not identified as relevant by the default method, but it is selected in this alternative one (with coefficient -0.1001).

- CHEK1 (Adjusted R^2_{M5} = 0.66 vs. 0.75): 7 relevant features identified by our default method (12 total features) are confirmed by this alternative method (23 total features), including gene methylation, with the following regression coefficients (the most relevant feature is the same in both methods, NFRKB):

Common Features	Coefficient (Our Method)	Coefficient (Lasso with feature re-eval)
NFRKB	0.4343	0.4790
RAD51	0.2605	0.1411
FANCA	0.2058	0.0845
DACH1	0.1073	0.1302
MAFK	-0.1611	-0.1214
RB1	-0.1680	-0.1609
METHYLATION (CHEK1)	-0.1144	-0.0937

- DNMT1 (Adjusted R^2_{M5} = 0.70 vs. 0.66): 4 relevant features identified by our default method (8 total features) are confirmed by this alternative method (6 total features), with the following regression coefficients (the most relevant feature is the same in both methods, SMARCA4):

**Common Features**	**Coefficient (Our Method)**	**Coefficient (Lasso with feature re-eval)**
SMARCA4	0.5505	0.6020
POLE	0.1738	0.2142
CHEK1	0.1565	0.1865
DEAF1	-0.1112	-0.0982

Both methods identify CHEK1 as one of the most relevant features, confirming the relevance of the experimentally confirmed DNMT1-CHEK1 biological relationship (main paper Results subsection *Experimental validation of found biological correlations in independent Ovarian and Breast cancer samples*).

- PTPRC (Adjusted R^2_{M5} = 0.82 vs. 0.84): all the relevant features identified by our default method, except one (ELF4), are confirmed by this method, with the following regression coefficients (the most relevant feature is the same in both methods, IKZF1):

**Common Features**	**Coefficient (Our Method)**	**Coefficient (Lasso with feature re-eval)**
IKZF1	0.5857	0.4559
ITGA4	0.2392	0.2398
CD38	0.1394	0.1615
CD44	0.1217	0.0980
AXL	0.0729	0.1118
ERCC2	-0.1169	-0.0586

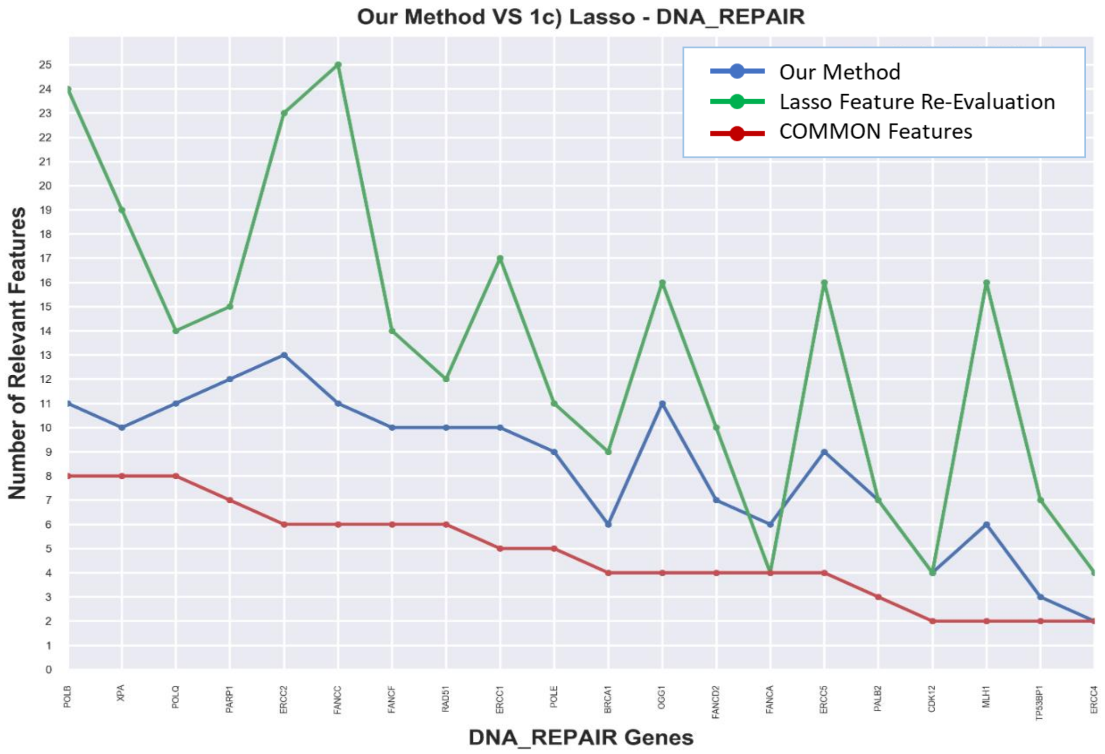

Figure S4.16: Number of relevant features selected by the two methods in the DNA_REPAIR pathway (Our Default Method vs. Lasso with Feature Re-evaluation).

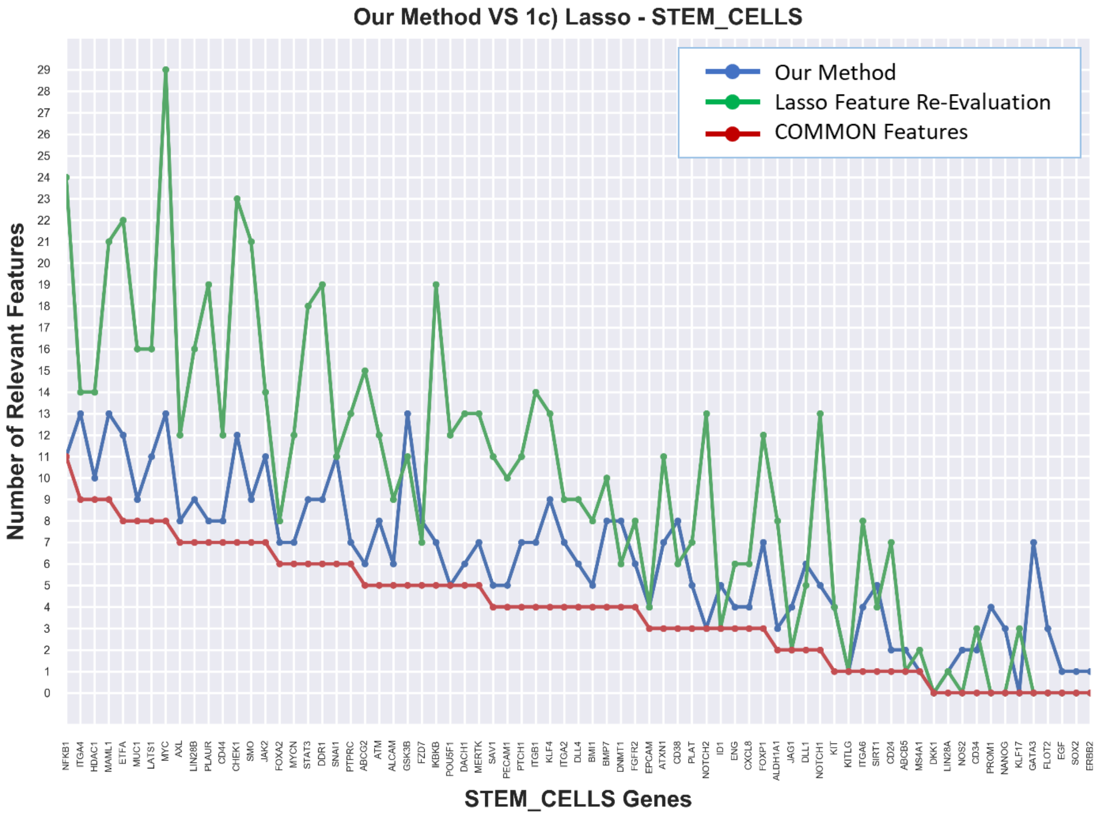

Figure S4.17: Number of relevant features selected by the two methods in the STEM_CELLS pathway (Our Default Method vs. Lasso with Feature Re-evaluation).

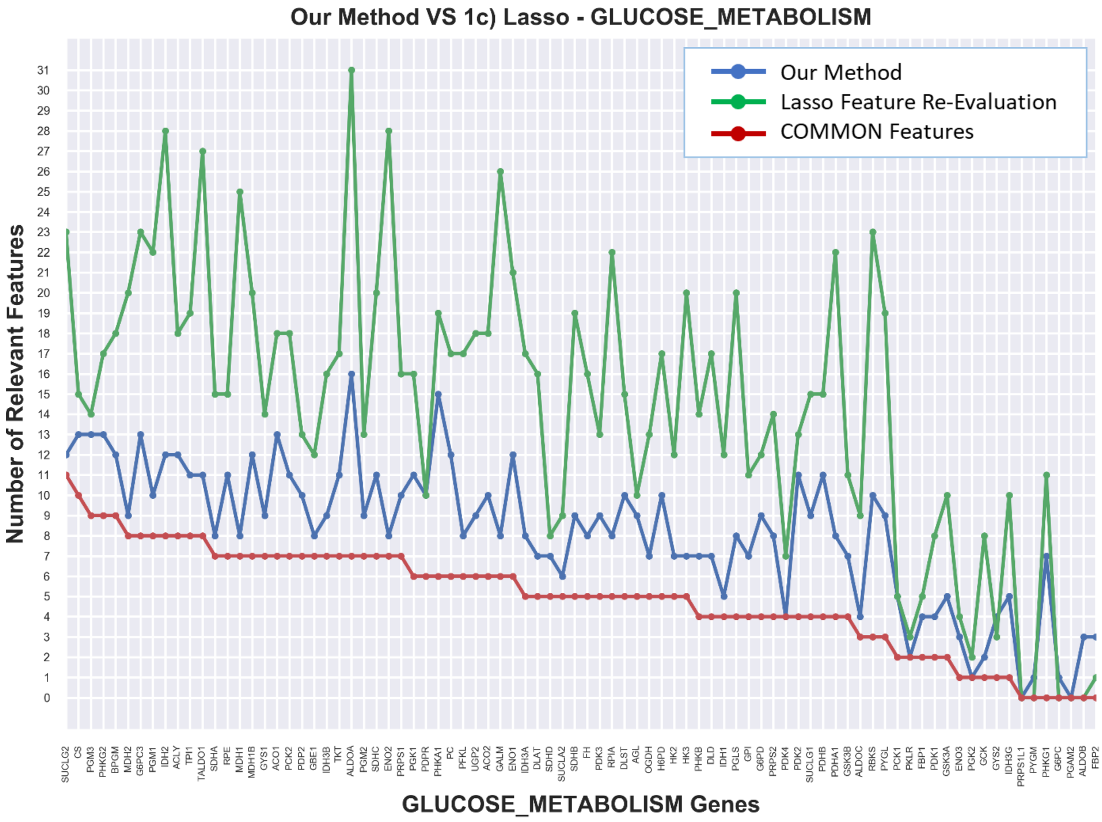

Figure S4.18: Number of relevant features selected by the two methods in the GLUCOSE_METABOLISM pathway (Our Default Method vs. Lasso with Feature Re-evaluation).

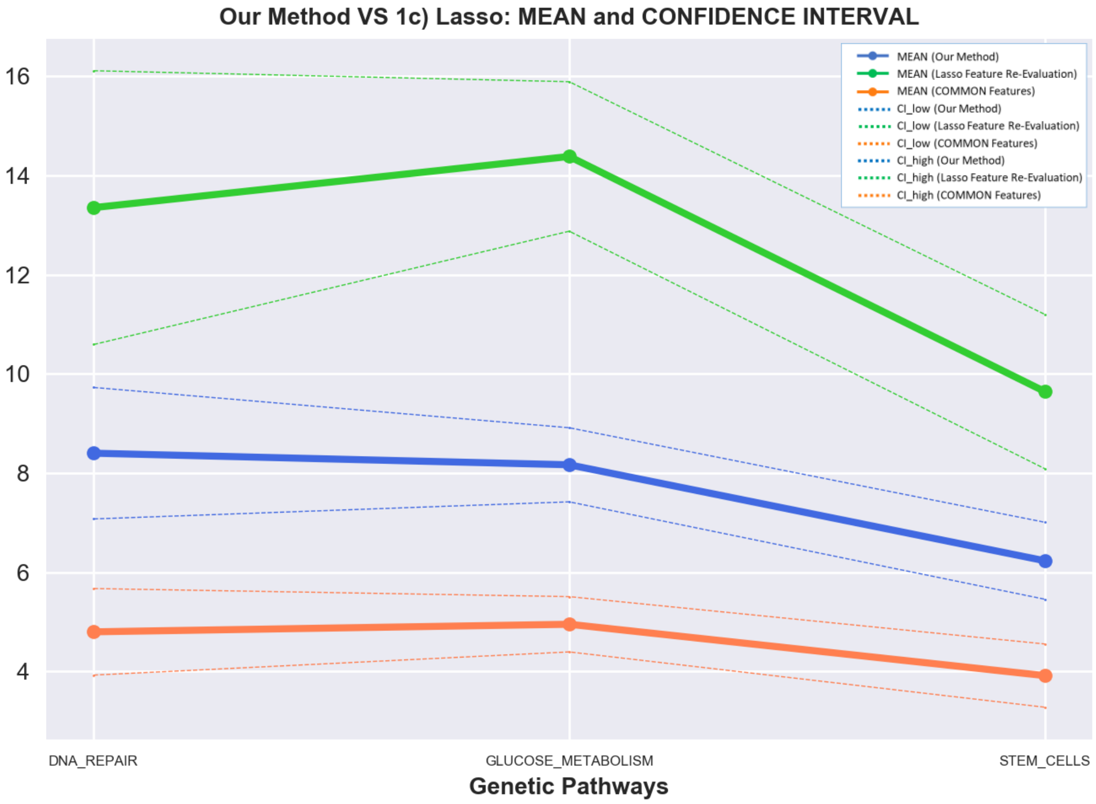

Figure S4.19: Mean and confidence interval plots of the number of relevant features selected by the two methods in the three pathways (Our Default Method vs. Lasso with Feature Re-evaluation).

	Our Default Method	
Lasso Feature Re-Evaluation	369	56
	14	0

$p \approx 0.14$

Figure S4.20: Fisher's exact test summary for all features extracted by the two methods (Our Default Method vs. Lasso with Feature Re-evaluation).

S4.2.4 Our incremental FFS method vs. Lasso on all features

- BRCA1 (Adjusted R^2_{M5} = 0.59 vs. 0.63): all the relevant features identified by our default method, except two of them (CBX5 and SUZ12), are confirmed by this alternative method, including gene methylation, with the following regression coefficients:

Common Features	Coefficient (Our Method)	Coefficient (Lasso on All Features)
FANCC	0.2336	0.1931
POLQ	0.2288	0.2177
ZBTB2	0.1160	0.1306
METHYLATION (BRCA1)	-0.3575	-0.3409

The alternative method identified 4 new relevant features, excluded by the default method: E2F7 (0.1431), HLTF (0.1134), SMARCE1 (0.1514) and STAT3 (0.1856).

- CDK12 (Adjusted R^2_{M5} = 0.43 vs. 0.41): all the relevant features identified by our default method, except 2 of them (ATM and NCOA3), are confirmed by this alternative method, with the following regression coefficients (the most relevant feature is the same in both methods, SUZ12):

**Common Features**	**Coefficient (Our Method)**	**Coefficient (Lasso on All Feature)**
SUZ12	0.3026	0.3481
STAT3	0.2293	0.2364

The alternative method identifies one new relevant feature, excluded by the default method: TAF1 (0.2696).

- ERCC1 (Adjusted R^2_{M5} = 0.60 vs. 0.56): 4 relevant features identified by our default method (10 total features) are confirmed by this method (10 total features), with the following regression coefficients (the most relevant feature is the same in both methods, ERCC2):

**Common Features**	**Coefficient (Our Method)**	**Coefficient (Lasso on All Features)**
ERCC2	0.5420	0.3601
HNRNPUL1	0.2743	0.2596
POLR2A	-0.1605	-0.2486
SKIL	-0.1874	-0.1594

These results confirm, even in this case, the strong relationship between ERCC1 and ERCC2 (experimentally confirmed in OV and BRCA PDX models). However, there is a different behavior with respect to the gene methylation, which is not identified as relevant by the default method, but it is selected in this alternative one (with coefficient -0.0898).

- CHEK1 (Adjusted R^2_{M5} = 0.66 vs. 0.67): 7 relevant features identified by our default method (12 total features) are confirmed by this alternative method (13 total features), including gene methylation, with the following regression coefficients (the most relevant feature is the same in both methods, NFRKB):

**Common Features**	**Coefficient (Our Method)**	**Coefficient (Lasso on All Features)**
NFRKB	0.4343	0.4588
RAD51	0.2605	0.2792
CBX5	0.1290	0.1871
DACH1	0.1073	0.0762
MAFK	-0.1611	-0.0898
RB1	-0.1680	-0.1562
METHYLATION (CHEK1)	-0.1144	0.0922

- DNMT1 (Adjusted R^2_{M5} = 0.70 vs. 0.60): only 2 relevant features identified by our default method (8 total features) are confirmed by this alternative method (2 total features), with the following regression coefficients (the most relevant feature is the same in both methods, SMARCA4):

Common Features	Coefficient (Our Method)	Coefficient (Lasso on All Features)
SMARCA4	0.5505	0.6908
CHEK1	0.1565	0.3066

Both methods identify CHEK1 as one of the most relevant features, confirming the relevance of the DNMT1-CHEK1 biological relationship, which has been confirmed in OV cancer PDX models and in a model of conditional CHECK1 knock-out in HCT116 cultured ovarian carcinoma cells (see main paper Results subsection *Experimental validation of found biological correlations in independent Ovarian and Breast cancer samples*).

- PTPRC (Adjusted R^2_{M5} = 0.82 vs. 0.83): all the relevant features identified by our default method, except 2 of them (ELF4 and ERCC2), are confirmed by this alternative method, with the following regression coefficients (the most relevant feature is the same in both methods, IKZF1):

Common Features	Coefficient (Our Method)	Coefficient (Lasso on All Features)
IKZF1	0.5857	0.4670
ITGA4	0.2392	0.2369
CD38	0.1394	0.1870
CD44	0.1217	0.0892
AXL	0.0729	0.1181

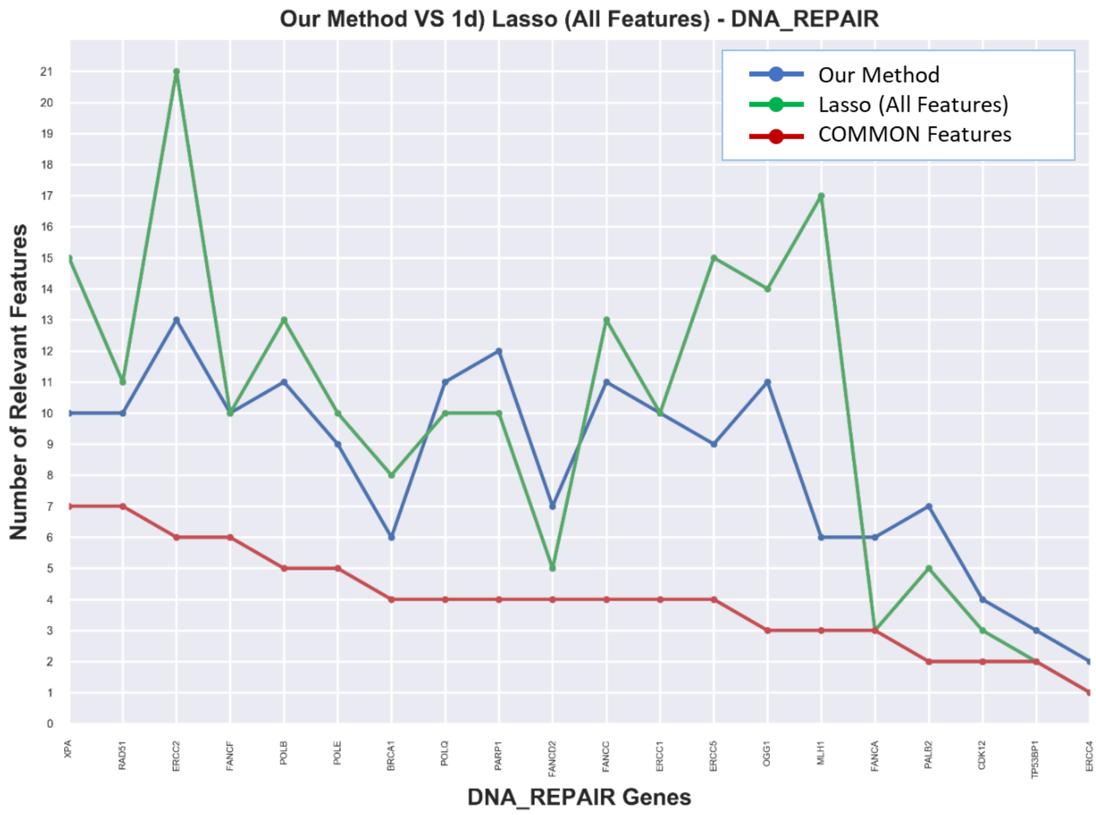

Figure S4.21: Number of relevant features selected by the two methods in the DNA_REPAIR pathway (Our Default Method vs. Lasso All Features).

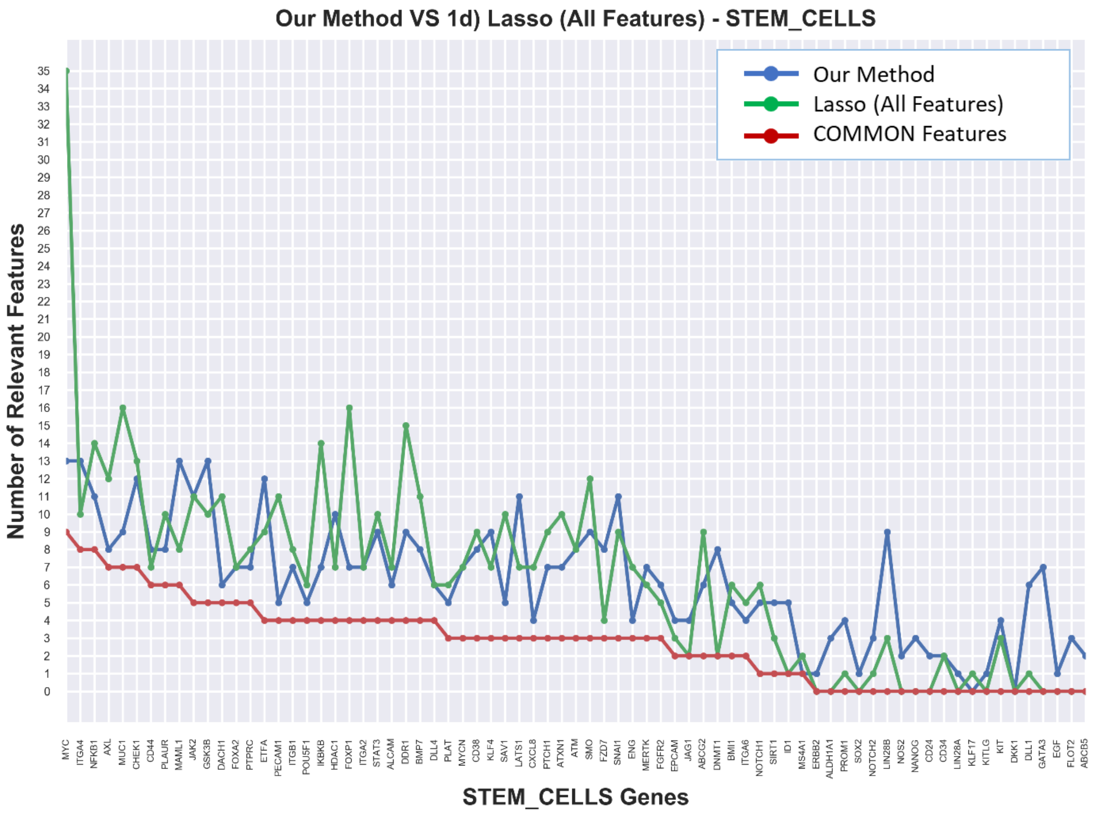

Figure S4.22: Number of relevant features selected by the two methods in the STEM_CELLS pathway (Our Default Method vs. Lasso All Features).

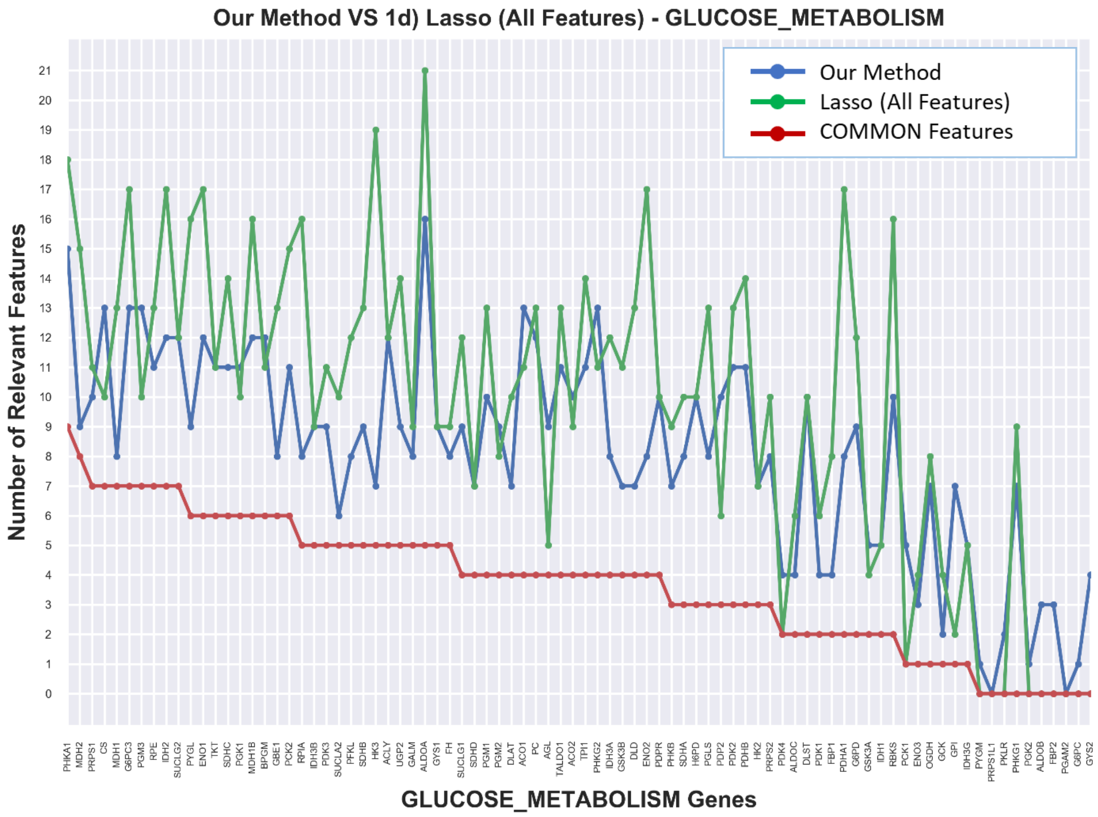

Figure S4.23: Number of relevant features selected by the two methods in the GLUCOSE_METABOLISM pathway (Our Default Method vs. Lasso All Features).

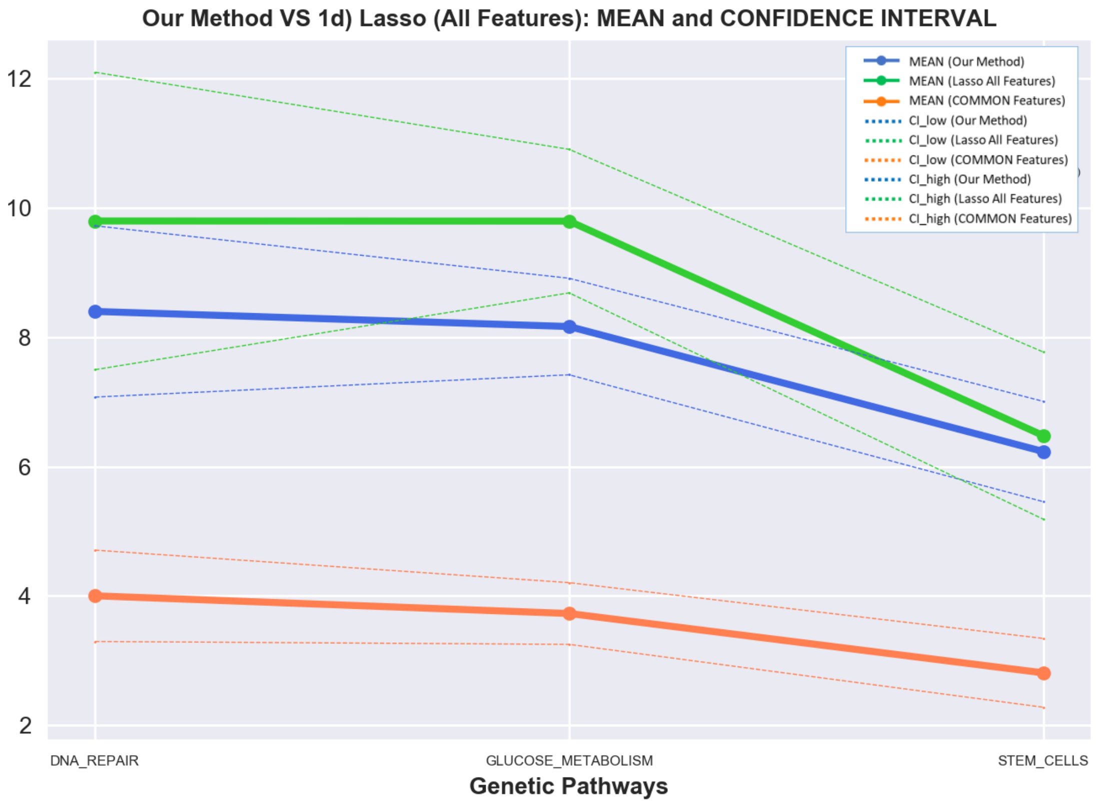

Figure S4.24: Mean and confidence interval plots of the number of relevant features selected by the two methods in the three pathways (Our Default Method vs. Lasso All Features).

	Our Default Method	
**Lasso (All Features)**	365	45
	18	0

$p \approx 0.13$

Figure S4.25: Fisher's exact test summary for all features extracted by the two methods (Our Default Method vs. Lasso All Features).

S4.2.5 Lasso on all features vs. FFS on all features

- BRCA1 (Adjusted R^2_{M5} = 0.63 vs. 0.62): only 4 relevant features, including gene methylation, are common to the two compared methods, out of 8 identified by Lasso and 10 identified by FFS:

Common Features	Coefficient (Lasso on All Features)	Coefficient (FFS on All Features)
FANCC	0.1931	0.2405
ZBTB2	0.1306	0.1868
SMARCE1	0.1514	0.1092
METHYLATION (BRCA1)	-0.3409	-0.3370

Here are the other features:

- Lasso (All Features): E2F7 (0.1431), HLTF (0.1134), POLQ (0.2177) and STAT3 (0.1856);
- FFS All Features: FANCD2 (0.2724), HDAC1 (-0.0847), HDAC2 (0.1074), OGG1 (-0.1029), SKIL (-0.0743) and SUZ12 (0.2100).

- CDK12 (Adjusted R^2_{M5} = 0.41 vs. 0.03): there are no common relevant features in this case. The Lasso method identifies 3 relevant features, STAT3 (0.2364), SUZ12 (0.3481) and TAF1 (0.2696), while the FFS method only one single feature, ZMIZ1 (0.1890).

- ERCC1 (Adjusted R^2_{M5} = 0.56 vs. 0.52): 4 relevant features identified by Lasso (7 total features) are confirmed by the FFS (10 total features), with the following regression coefficients (the most relevant feature is the same in both methods, ERCC2):

**Common Features**	**Coefficient (Lasso on All Features)**	**Coefficient (FFS on All Features)**
ERCC2	0.3601	0.4665
HNRNPUL1	0.2596	0.3395
OGG1	0.1404	0.1047
SKIL	-0.1594	-0.1814

These results confirm, even in this case, the strong relationship between ERCC1 and ERCC2 (experimentally confirmed in OV and BRCA PDX models), identifying ERCC2 as the most relevant feature in both methods. However, there is a different behavior with respect to the gene methylation, which is not identified as relevant by the FFS method, but it is selected in the Lasso one (with coefficient -0.0898).

- CHEK1 (Adjusted R^2_{M5} = 0.67 vs. 0.64): 4 relevant features identified by Lasso (13 total features) are confirmed by the FFS (8 total features), with the following regression coefficients (the most relevant feature is the same in both methods, NFRKB):

**Common Features**	**Coefficient (Lasso on All Features)**	**Coefficient (FFS on All Features)**
NFRKB	0.4588	0.5139
RAD51	0.2792	0.3062
MAFK	-0.2237	-0.0898
RB1	-0.2861	-0.1562

However, there is a different behavior with respect to the gene methylation, which is not identified as relevant by the FFS method, but it is selected in the Lasso one (with coefficient -0.0922).

- DNMT1 (Adjusted R^2_{M5} = 0.60 vs. 0.62): only 1 relevant feature identified by Lasso (out of the 2 total features) is confirmed by the FFS (4 total features), with the following regression coefficient (the single common feature is the most relevant feature in both methods, SMARCA4):

**Common Features**	**Coefficient (Lasso on All Features)**	**Coefficient (FFS on All Features)**
SMARCA4	0.6908	0.6402

However, only Lasso identifies CHEK1 as one of the most relevant features, despite the relationship between DNMT1 and CHEK1 has been confirmed in OV cancer PDX models and in a model of

conditional CHECK1 knock-out in HCT116 cultured ovarian carcinoma cells (see main paper Results subsection *Experimental validation of found biological correlations in independent Ovarian and Breast cancer samples*).

- PTPRC (Adjusted R^2_{M5} = 0.83 vs. 0.82): only 4 relevant features are common to the two compared methods, out of 8 identified by Lasso and 6 identified by FFS (the most relevant feature is the same in both methods, IKZF1):

**Common Features**	**Coefficient (Lasso on All Features)**	**Coefficient (FFS on All Features)**
IKZF1	0.4670	0.6340
ITGA4	0.2369	0.2328
CD38	0.1870	0.1075
CD44	0.0892	0.0922

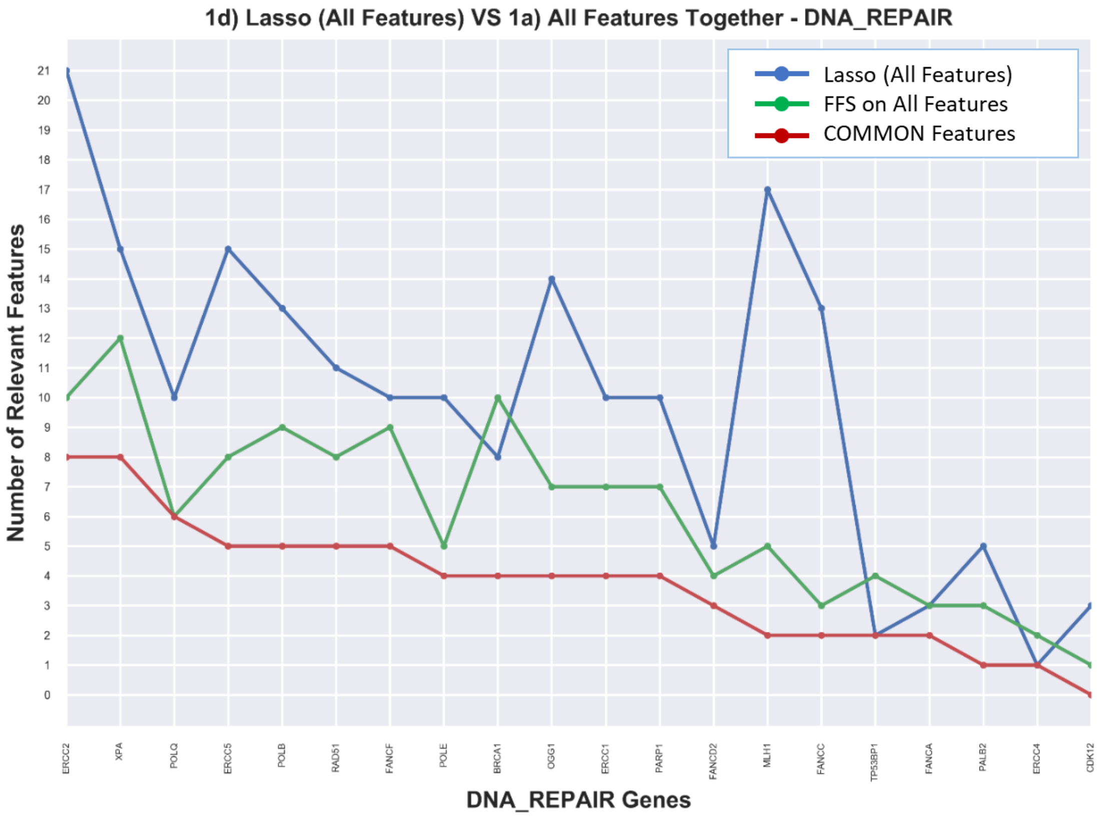

Figure S4.26: Number of relevant features selected by the two methods in the DNA_REPAIR pathway (Lasso All Features vs. FFS All Features).

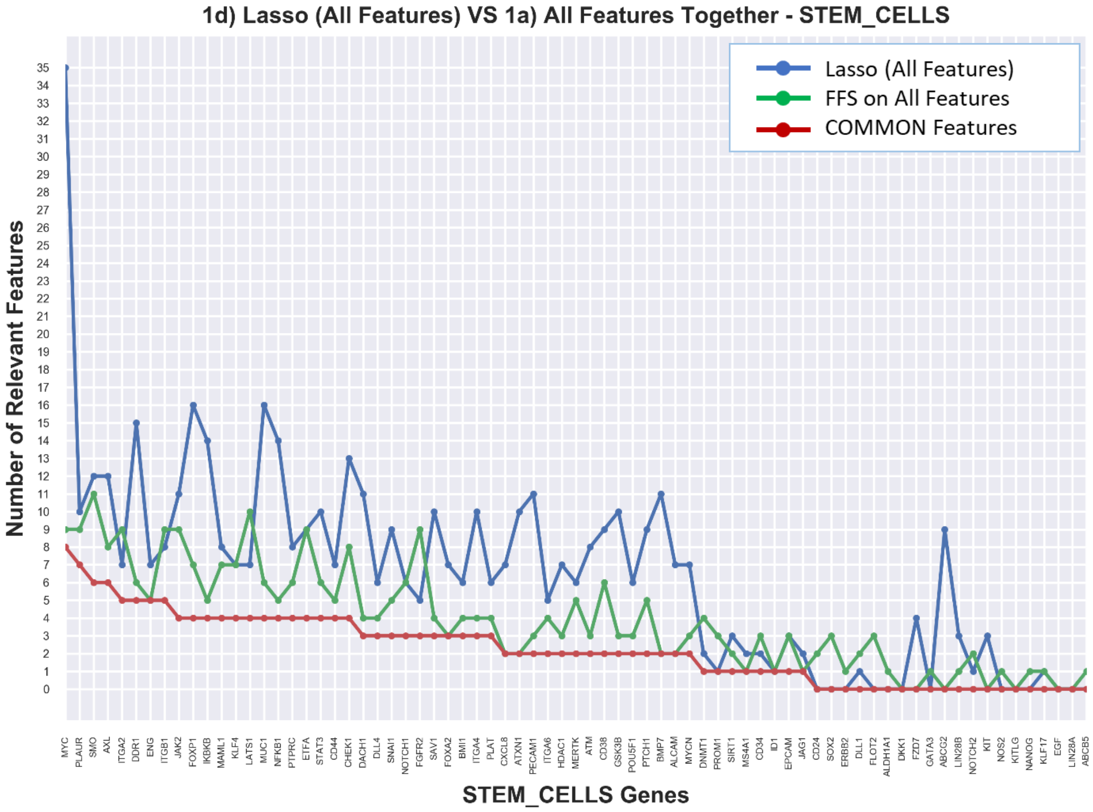

Figure S4.27: Number of relevant features selected by the two methods in the STEM_CELLS pathway (Lasso All Features vs. FFS All Features).

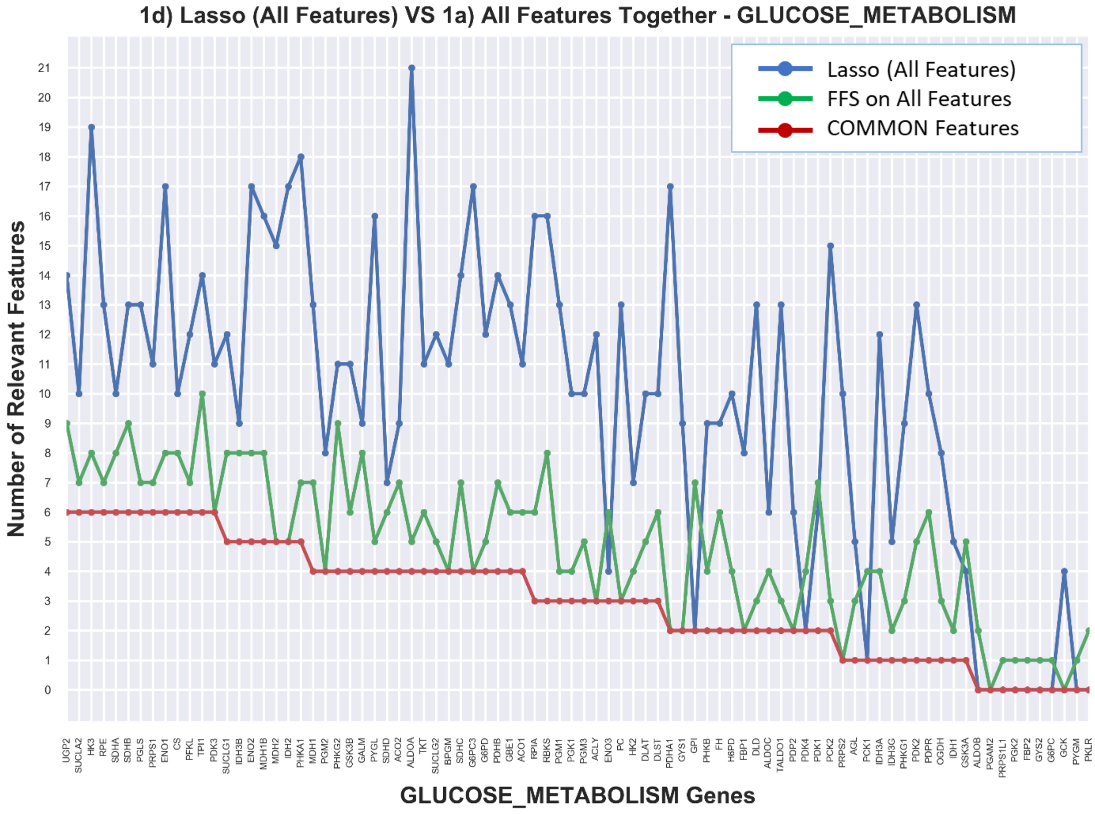

Figure S4.28: Number of relevant features selected by the two methods in the GLUCOSE_METABOLISM pathway (Lasso All Features vs. FFS All Features).

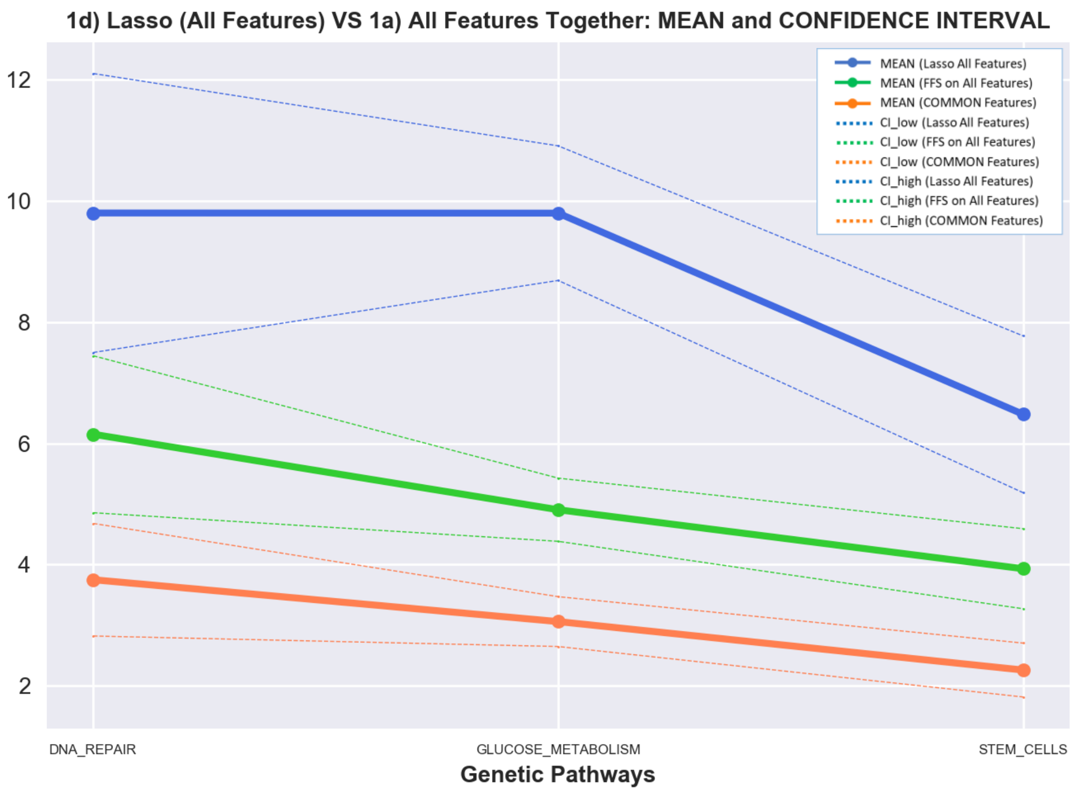

Figure S4.29: Mean and confidence interval plots of the number of relevant features selected by the two methods in the three pathways (Lasso All Features vs. FFS All Features).

	Lasso (All Features)	
FFS All Features	328	16
	82	0

$p \approx 0.03$

Figure S4.30: Fisher's exact test summary for all features extracted by the two methods (Lasso All Features vs. FFS All Features).

S4.2.6 Overall summary comparison of the considered methods

Here, we report the overall comparison among the five feature selection methods considered, based on the number of features that each method extracts and their consensus with the other methods, or on the number of target genes for which a method could extract significant features and the goodness of these features in modelling the target gene expression according to the Bayesian Information Criterion (BIC), or on the method execution time.

For each method, the following table shows the total number of features extracted and those of them selected also by other methods (all four or less), or by no other method, while Figure S4.31 illustrates the percentage of the features extracted by a method that were selected also by all other 4 methods, and the percentage of the features not selected by all other methods that were selected by 3, 2, 1 other methods or by none of them:

Method	Total Extracted	Found in All 4 Other Methods	Found in 3 Other Methods	Found in 2 Other Methods	Found in 1 Other Method	Found in NO Other Method
Incremental FFS with Feature Re-Evaluation (Our Method)	383	307	55	16	4	1
Incremental FFS with NO Feature Re-Evaluation	425	307	66	31	15	6
FFS on All Features	344	307	20	11	6	0
Lasso with Feature Re-Evaluation	425	307	64	33	16	5
Lasso on All Features	410	307	63	29	11	0

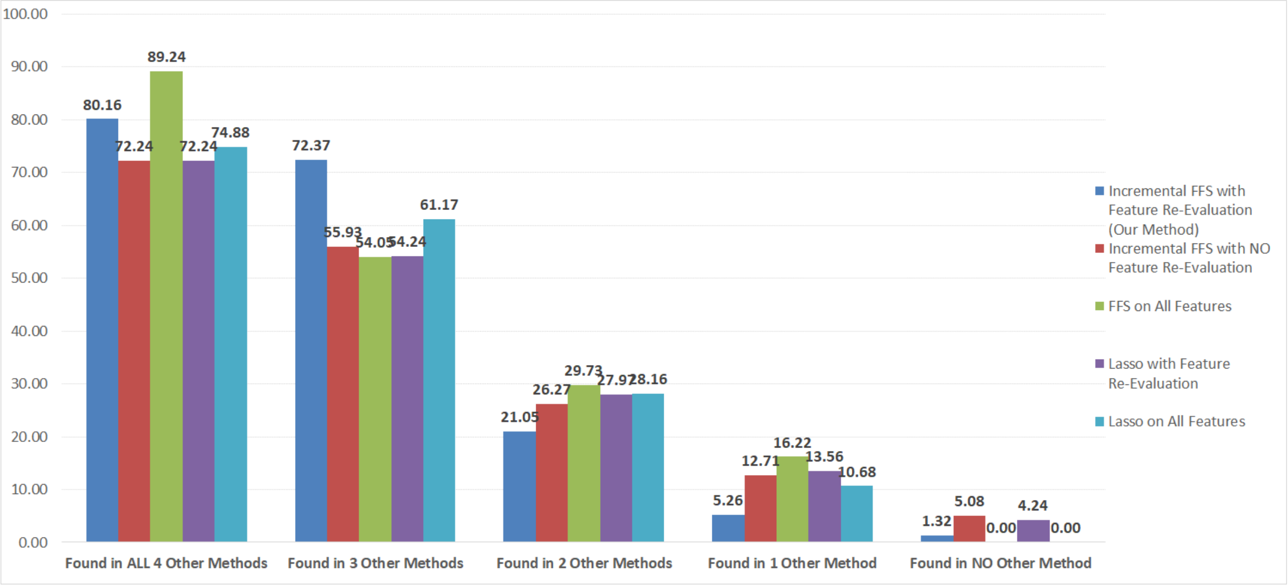

Figure S4.31: Percentage of the features selected by a method that were selected also by other methods or not.

For each feature selection method considered, the following table shows the number of target genes (Models) out of the 177 total evaluated ones for which the method could extract significant features, and the average and standard deviation of the Bayesian Information Criterion (BIC) for these models, computed on the M5 data matrix features of the target gene or on all its features (BIC is a well-known criterion for model selection among a finite set of models, whose lowest value indicates the preferred model):

Method	Models	BIC Average	BIC Standard Deviation
Incremental FFS with Feature Re-Evaluation (Our Method)	175	881.999	120.167
Incremental FFS with NO Feature Re-Evaluation	176	894.394	115.206
FFS on All Features	171	909.464	115.063
Lasso with Feature Re-Evaluation	163	834.509	115.968
Lasso on All Features	155	841.545	106.681

For each feature selection method considered, the following table shows the execution times (in minutes, on a 4 core CPU, 2.80 GHz processor, 16 GB RAM computer) for each considered gene set (pathway), feature set (M2, M3 and M5 data matrix), and overall:

**Pathway**	**M2**	**M3**	**M5**	**All**
Incremental FFS with Feature Re-Evaluation (Our Method)				
DNA_REPAIR	150	120	50	320
STEM_CELLS	750	1,050	20	1,820
GLUCOSE_METABOLISM	1,300	1,020	390	2,710
All three together				4,850
Incremental FFS with NO Feature Re-Evaluation				
DNA_REPAIR	150	130	40	320
STEM_CELLS	750	990	10	1,750
GLUCOSE_METABOLISM	1,300	840	210	2,350
All three together				4,420
FFS on All Feature				
DNA_REPAIR				1,380
STEM_CELLS				5,100
GLUCOSE_METABOLISM				13,020
All three together				19,500
Lasso with Feature Re-Evaluation				
DNA_REPAIR	1.5	2.5	2.5	6.5
STEM_CELLS	2.5	3.5	4.25	10.25
GLUCOSE_METABOLISM	3.0	4.25	6.0	13.25
All three together				23.5
Lasso on All Feature				
DNA_REPAIR				2.5
STEM_CELLS				5.0
GLUCOSE_METABOLISM				8.5
All three together				13.5

S4.2.7 Common features in all 5 feature selection methods

Here, we list the common features identified in all 5 methods considered, for each target gene in OV tumor. These features can be considered as most reliable since they were unveiled by all the different methods:

GENE_SYMBOL	PATHWAY	COMMON FEATURES
BRCA1	DNA_REPAIR	FANCC, METHYLATION (BRCA1), ZBTB2
CDK12	DNA_REPAIR	-
ERCC1	DNA_REPAIR	ERCC2, HNRNPUL1, SKIL
ERCC2	DNA_REPAIR	ERCC1, TEAD2
ERCC4	DNA_REPAIR	CREB3
ERCC5	DNA_REPAIR	CHAMP1, METHYLATION (ERCC5)
FANCA	DNA_REPAIR	MYBL2, POLE
FANCC	DNA_REPAIR	POLE, XPA
FANCD2	DNA_REPAIR	MLH1, OGG1, POLQ
FANCF	DNA_REPAIR	METHYLATION (FANCF), SOX6, ZNF143
MLH1	DNA_REPAIR	-

OGG1	DNA_REPAIR	E2F1, FANCD2, NR2C2
PALB2	DNA_REPAIR	MAZ
PARP1	DNA_REPAIR	BRCA1, TRIM25, ZC3H11A
POLB	DNA_REPAIR	IKBKB, THAP1
POLE	DNA_REPAIR	FANCA, FANCF
POLQ	DNA_REPAIR	FANCD2, GSK3B, OGG1, ZBTB11
RAD51	DNA_REPAIR	CHEK1, FOXM1, NFE2, YBX3
TP53BP1	DNA_REPAIR	ZSCAN29
XPA	DNA_REPAIR	ARID1B, FANCC, ZHX1
ABCB5	STEM_CELLS	-
ABCG2	STEM_CELLS	-
ALCAM	STEM_CELLS	ZBTB11
ALDH1A1	STEM_CELLS	-
ATM	STEM_CELLS	METHYLATION (ATM), NFRKB
ATXN1	STEM_CELLS	TFDP1, ZNF592
AXL	STEM_CELLS	ETFA, ITGA4, PTPRC, PTRF
BMI1	STEM_CELLS	MAML1, TBP
BMP7	STEM_CELLS	PAX8, ZNF83
CD24	STEM_CELLS	-
CD34	STEM_CELLS	-
CD38	STEM_CELLS	PTPRC, TRIM22
CD44	STEM_CELLS	NFIC, PAX8, PTPRC
CHEK1	STEM_CELLS	NFRKB, RAD51, RB1
DACH1	STEM_CELLS	PTCH1, TRIM22
DDR1	STEM_CELLS	PBX2, ZNF184
DKK1	STEM_CELLS	-
DLL1	STEM_CELLS	-
DLL4	STEM_CELLS	ENG, IKZF1, PECAM1
DNMT1	STEM_CELLS	SMARCA4
EGF	STEM_CELLS	-
ENG	STEM_CELLS	DLL4, MEF2D, ZEB2
EPCAM	STEM_CELLS	MTA3
ERBB2	STEM_CELLS	-
ETFA	STEM_CELLS	GTF2A2, PML, SIN3A
FGFR2	STEM_CELLS	EP300, LEF1, SOX6
FLOT2	STEM_CELLS	-
FOXA2	STEM_CELLS	ABCB5, DLL1
FOXP1	STEM_CELLS	ATF7, LARP7
FZD7	STEM_CELLS	-
GATA3	STEM_CELLS	-
GSK3B	STEM_CELLS	HLTF
HDAC1	STEM_CELLS	TOE1, ZHX1
ID1	STEM_CELLS	ID3
IKBKB	STEM_CELLS	POLB
CXCL8	STEM_CELLS	METHYLATION (CXCL8), PLAUR

ITGA2	STEM_CELLS	E2F1, ELF1, GTF2F1
ITGA4	STEM_CELLS	AXL, MEF2A
ITGA6	STEM_CELLS	-
ITGB1	STEM_CELLS	CREM, GSK3B, PTRF, ZBTB5
JAG1	STEM_CELLS	HES1
JAK2	STEM_CELLS	DDX20, IRF1, NFIB, TRIM22
KIT	STEM_CELLS	-
KITLG	STEM_CELLS	-
KLF17	STEM_CELLS	-
KLF4	STEM_CELLS	NOTCH1
LATS1	STEM_CELLS	ARID1B, BCLAF1, ELF4
LIN28A	STEM_CELLS	-
LIN28B	STEM_CELLS	-
MAML1	STEM_CELLS	ELF1, HCFC1, ZNF354B
MERTK	STEM_CELLS	ITGA4
MS4A1	STEM_CELLS	CD38
MUC1	STEM_CELLS	SMO
MYC	STEM_CELLS	MAX, MXI1, NKRF, YBX3
MYCN	STEM_CELLS	MTA3
NANOG	STEM_CELLS	-
NFKB1	STEM_CELLS	ELF1, LARP7, RELA
NOS2	STEM_CELLS	-
NOTCH1	STEM_CELLS	JAG1
NOTCH2	STEM_CELLS	-
PECAM1	STEM_CELLS	-
PLAT	STEM_CELLS	LEF1, METHYLATION (PLAT)
PLAUR	STEM_CELLS	BHLHE40, CREB3L1, CXCL8, KLF16, ZHX1
POU5F1	STEM_CELLS	METHYLATION (POU5F1), U2AF1
PROM1	STEM_CELLS	-
PTCH1	STEM_CELLS	FANCC
PTPRC	STEM_CELLS	CD38, CD44, IKZF1, ITGA4
SAV1	STEM_CELLS	METHYLATION (SAV1), ZBTB1
SIRT1	STEM_CELLS	CREB1
SMO	STEM_CELLS	MUC1, NRF1, TRIM24
SNAI1	STEM_CELLS	BHLHE40, NRF1
SOX2	STEM_CELLS	-
STAT3	STEM_CELLS	DACH1, SMARCE1, STAT5A, ZNF579
ACLY	GLUCOSE_METABOLISM	MEF2D, SMARCE1
ACO1	GLUCOSE_METABOLISM	GYS1, NFIB, PGM3, ZBTB5
ACO2	GLUCOSE_METABOLISM	ESRRA, IDH3A, L3MBTL2
AGL	GLUCOSE_METABOLISM	ZNF644
ALDOA	GLUCOSE_METABOLISM	ENO3, MAZ, MDH2, PHKG2
ALDOB	GLUCOSE_METABOLISM	-
ALDOC	GLUCOSE_METABOLISM	FLOT2, METHYLATION (ALDOC)
BPGM	GLUCOSE_METABOLISM	DLD, MYC, PROM1

CS	GLUCOSE_METABOLISM	ACO2, CBX5, KAT8, SP1, YBX3, ZHX1
DLAT	GLUCOSE_METABOLISM	ATM, SDHD
DLD	GLUCOSE_METABOLISM	METHYLATION (DLD), ZKSCAN1
DLST	GLUCOSE_METABOLISM	MAX
ENO1	GLUCOSE_METABOLISM	GBE1, H6PD, SDHB, SDHC
ENO2	GLUCOSE_METABOLISM	MTA2, SUCLG1, TPI1
ENO3	GLUCOSE_METABOLISM	PHKG2
FBP1	GLUCOSE_METABOLISM	HK3
FBP2	GLUCOSE_METABOLISM	-
FH	GLUCOSE_METABOLISM	PARP1
G6PC	GLUCOSE_METABOLISM	-
G6PC3	GLUCOSE_METABOLISM	ACLY, CREB1, PDK2, UBTF
G6PD	GLUCOSE_METABOLISM	IDH3G
GALM	GLUCOSE_METABOLISM	CD38, MTA3, PDHA1, PGM2
GBE1	GLUCOSE_METABOLISM	HK2, MITF, PGK1
GCK	GLUCOSE_METABOLISM	-
GPI	GLUCOSE_METABOLISM	CEBPG
GSK3A	GLUCOSE_METABOLISM	HNRNPUL1
GSK3B	GLUCOSE_METABOLISM	HLTF, ZBTB11
GYS1	GLUCOSE_METABOLISM	ERCC2, PFKL
GYS2	GLUCOSE_METABOLISM	-
H6PD	GLUCOSE_METABOLISM	ENO1, ETV6
HK2	GLUCOSE_METABOLISM	PDK1
HK3	GLUCOSE_METABOLISM	FBP1, PTPRC
IDH1	GLUCOSE_METABOLISM	RPE
IDH2	GLUCOSE_METABOLISM	MEF2A, NR2C2, ZNF592
IDH3A	GLUCOSE_METABOLISM	-
IDH3B	GLUCOSE_METABOLISM	METHYLATION (IDH3B), SDHB, SMARCB1, ZFX
IDH3G	GLUCOSE_METABOLISM	G6PD
MDH1	GLUCOSE_METABOLISM	SRSF7, TAF7, UGP2
MDH1B	GLUCOSE_METABOLISM	E2F7, MTA2, TCF7
MDH2	GLUCOSE_METABOLISM	ALDOA, IDH2, MCM7, METHYLATION (MDH2), SDHB
OGDH	GLUCOSE_METABOLISM	ZNF207
PC	GLUCOSE_METABOLISM	DPF2, ELF4
PCK1	GLUCOSE_METABOLISM	METHYLATION (PCK1)
PCK2	GLUCOSE_METABOLISM	IRF9
PDHA1	GLUCOSE_METABOLISM	PRPS2
PDHB	GLUCOSE_METABOLISM	SDHB, SUCLG2
PDK1	GLUCOSE_METABOLISM	HK2
PDK2	GLUCOSE_METABOLISM	RUNX1
PDK3	GLUCOSE_METABOLISM	CD44, MYC, PRPS1, ZFX
PDK4	GLUCOSE_METABOLISM	ABCG2
PDP2	GLUCOSE_METABOLISM	E2F4, NFATC3
PDPR	GLUCOSE_METABOLISM	ZNF143
PFKL	GLUCOSE_METABOLISM	BACH1, GYS1, TOE1

PGAM2	GLUCOSE_METABOLISM	-
PGK1	GLUCOSE_METABOLISM	ALDOA, PRPS1, UGP2
PGK2	GLUCOSE_METABOLISM	-
PGLS	GLUCOSE_METABOLISM	NR2F6, SIN3B
PGM1	GLUCOSE_METABOLISM	ENO1, MIER1, PYGL
PGM2	GLUCOSE_METABOLISM	MYC, NFKB1, PGM3
PGM3	GLUCOSE_METABOLISM	METHYLATION (PGM3), ZNF184
PHKA1	GLUCOSE_METABOLISM	SMO, TAF1, TCF7L2
PHKB	GLUCOSE_METABOLISM	NFATC3, POLR2H
PHKG1	GLUCOSE_METABOLISM	-
PHKG2	GLUCOSE_METABOLISM	ALDOA, KAT8
PKLR	GLUCOSE_METABOLISM	-
PRPS1	GLUCOSE_METABOLISM	NKRF, NONO, PCK2, PDK3, PGK1
PRPS1L1	GLUCOSE_METABOLISM	-
PRPS2	GLUCOSE_METABOLISM	-
PYGL	GLUCOSE_METABOLISM	SAV1
PYGM	GLUCOSE_METABOLISM	-
RBKS	GLUCOSE_METABOLISM	FOSL2
RPE	GLUCOSE_METABOLISM	CREB1, IDH1, METHYLATION (RPE), PPP1R10
RPIA	GLUCOSE_METABOLISM	MERTK, SUCLG1
SDHA	GLUCOSE_METABOLISM	ACO2, CREB3, METHYLATION (SDHA)
SDHB	GLUCOSE_METABOLISM	ENO1, MDH2, RBKS, SUCLG1
SDHC	GLUCOSE_METABOLISM	PYGO2, SUCLG1
SDHD	GLUCOSE_METABOLISM	ACLY, DLAT, SUCLG1
SUCLA2	GLUCOSE_METABOLISM	ELF1, PDHB, RB1, RFX5, STAT5A
SUCLG1	GLUCOSE_METABOLISM	MDH1, RPIA
SUCLG2	GLUCOSE_METABOLISM	PC, PDHB, PGM2
TALDO1	GLUCOSE_METABOLISM	DEAF1
TKT	GLUCOSE_METABOLISM	MYC, REST, SREBF1
TPI1	GLUCOSE_METABOLISM	ENO2, PGM1, PHB2, YBX3
UGP2	GLUCOSE_METABOLISM	MDH1, NCOA1, PDK2, PYGO2
